# Reduced Radial Electric Quadrupole Moment Function for Diatomic Molecules

**DOI:** 10.1021/acs.jctc.4c01410

**Published:** 2024-12-11

**Authors:** Vladimír Špirko

**Affiliations:** Institute of Organic Chemistry and Biochemistry, p.r.i., Czech Academy of Sciences, Flemingovo nám. 2 Prague 6, Praha 166 10, Czechia

## Abstract

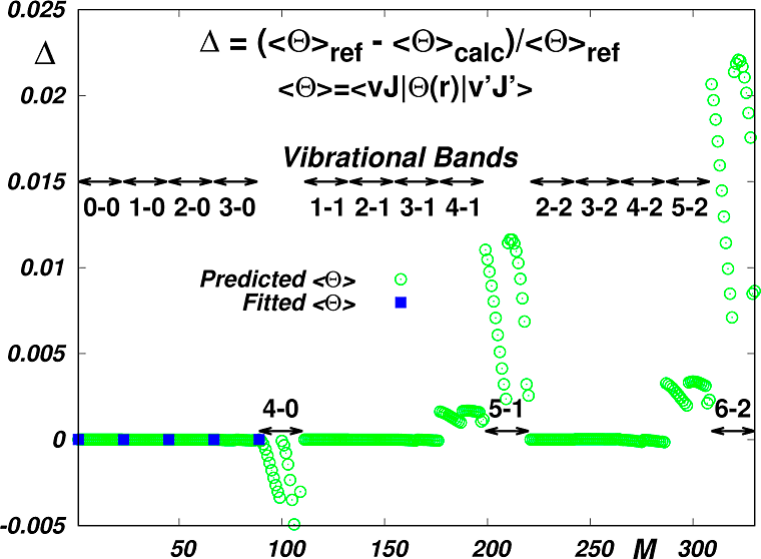

The prospect of constructing global electric quadrupole moment functions (EQMFs) of diatomic molecules by morphing their theoretical approximants within the framework of the reduced radial curve (RRC) approach is explored by performing model calculations for the ground electronic states of H_2_ and HF. The reduced quadrupole moment curves probed, constructed for a set of differently accurate theoretical EQMFs, coincide with their best many-parameter analytic counterparts so closely that they can be used as their accurate few-parameter representations. No other such functional representation is available in the literature.

## 1 Introduction

As the first possible nonzero electric moment of a nonpolar molecule, the molecular electric quadrupole moment (EQM) is a property of special importance. For instance, the quadrupole moment allows for molecular absorption of infrared radiation. Although the corresponding transitions are typically a million times weaker than electric dipole transitions, they are important for accurate atmospheric and astronomical remote sensing (see e.g. refs (1 and 2)). This applies in particular to the hydrogen molecule H_2_, which is the most abundant molecule in the Universe. Importantly, due to its simplicity, H_2_ also serves as an ideal test system for high-level calculations in quantum mechanics (see e.g. ref (3)) and investigations in fundamental physics (see e.g. ref (4)).

Obviously, molecular hydrogen is not the only molecule for which it is desirable to know the quadrupole moment. As pointed out in refs (2 and 5), other such molecules are, for instance, N_2_, S_2_ and O_2_, which are expected to play an important role in analyses of atmospheric spectra of hot exoplanets. To account for the role of these molecules, adequately accurate radial electric quadrupole moment functions (Θ(*r*)) are needed. In practice, such functions can be evaluated using either the first-principles quantum-chemical approaches (see e.g. ref (6)) or by fitting them to appropriate experimental data (see e.g. ref (7)). However, both of these approaches suffer from serious drawbacks. First-principles-based functions only rarely achieve “spectroscopic” accuracy, and their fitting is severely limited by a notorious scarcity of experimental data, confounded by the fact that the generic shape of any fitted function is a priori unknown (note also that unlike in the case of dipole moment functions, separated and united atom limits may have nonvanishing moments). Therefore, unsurprisingly, no reliable experimental EQMF has yet been devised, and the accuracy and coverage of available theoretical functions is more or less questionable. Of course, the previous statement does not pertain to the hydrogen molecule, the theoretical functions for which are much more accurate than the current limits of experimentation (see ref (6) and references therein).

To evaluate measurable quantities and their accuracy, pointwise defined theoretical functions need to be interpolated using some sort of functional form. As shown recently,^7^ one way of constructing such analytical EQMF interpolants can be based on fitting suitable analytic functions to available data while imposing certain mathematical restrictions.^8,9^ Actually, two such functions are presented for the ground electronic state of H_2_ in ref (7). One has branching points in the complex plane but no poles and, conversely, the other has poles but no branching points. Importantly, despite possessing different singularities, the functions provide closely coinciding intensities even for higher overtones, which can be strongly affected by these singularities. The functions therefore appear to be a suitable basis for high-precision interpolation of piecewise defined ab initio data. However, given the fact that one includes 15 and the other 16 fitting parameters, and considering the usual scarcity of accurate experimental data, the functions are highly impractical for fitting. Moreover, their analogue obtained by the separated and united atom limits may have nonvanishing momentssimultaneous fitting of both theoretical and experimental data evidence a fairly strong disharmony in describing theory and experiment.^7^ As recently proposed in ref (10) and successfully applied in ref (11), one way how to overcome this problem may rely on the homotopic morphing of approximate, but topologically correct, functions by fitting to accurate reference data within the framework of Jenč’s basically ’three-parameter’ reduced potential energy curve scheme.^12,14^

## 2 Modeling

To test the prospects of the RRC approach, let us first consider model calculations for H_2_: (a) The basic reference data used in this modeling, the rovibrational quadrupole transition moments ⟨*vJ*|Θ(*r*)|*v*^′^*J*^′^⟩ (*v* and *J* being the vibrational and rotational quantum numbers, respectively), are evaluated based on experimental line intensities, on the one hand, and using the H_2_ potential energy function from Komasa at al.^15^ and quadrupole moment function from Wolniewicz et al.,^16^ on the other. The experimental reference data file includes the data summarized in ref (6) and data taken from refs (17–21) (see Table S1 in the Supporting Information). The first part of the theoretical reference data set was obtained from its experimental counterpart by replacing the experimental transition moments with their theoretical reference values, and its second part consists of theoretical data representing higher spectral overtones (see Table S2 in the Supporting Information).

(b) To gain insight into the role of the accuracy of the morphed functions, four additional EQMFs from the literature are probed, namely two less accurate Wolniewicz-type EQMFs, obtained using explicitly correlated Gaussian functions (see refs (22–24)), and the “standard” CISD/d-aug-cc-pV6Z and CCSD/d-aug-cc-pV6Z EQMFs from refs (25 and 26) respectively. The first three of these pointwise defined functions were fitted using exponential polynomials (see Tables S3–S5), and the last one (see Table S6) was interpolated using cubic splines (note that interpolating using standard global polynomials is badly plagued by the Runge phenomenon, see e.g. refs (27)).

(c) The RRC approach adopted consists of two steps (for an excellent and detailed introduction to the theory of reduced radial functions see ref (13)). First, the reduced forms θ(ρ) of the probed Θ^ref^(*r*) functions are generated using the following definition
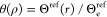
1where Θ_e_^ref^ is the depth of Θ^ref^(*r*) and the reduced variable ρ is related to *r* via the expression
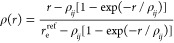
2in which r_e_^ref^ is the distance for which Θ^ref^(*r*) attains its minimum and ρ_*ij*_ satisfies the transcendental equation
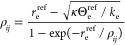
3where
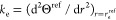
4where κ is a “universal-shape” constant (κ can be any constant allowing a numerically stable solution of the transcendental eq 2 for ρ_*ij*_).

In the second step, the reducing procedure is inverted by expressing Θ(*r*) as a function of θ(ρ)
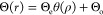
5with ρ defined by
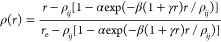
6where the a priori unknown parameters Θ_o_, Θ_e_, *r*_e_, ρ_*ij*_, α, β and γ are to be determined by fitting the reference ro-vibrational transition moments  given in Tables S1, S2, S7 and S8 (note that in the standard RRC scheme α = β = 1 and γ = 0). Reproduction of the reference data by their counterparts provided by the fitted functions is measured using the following quantity
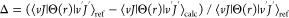
7

**Figure 1:**
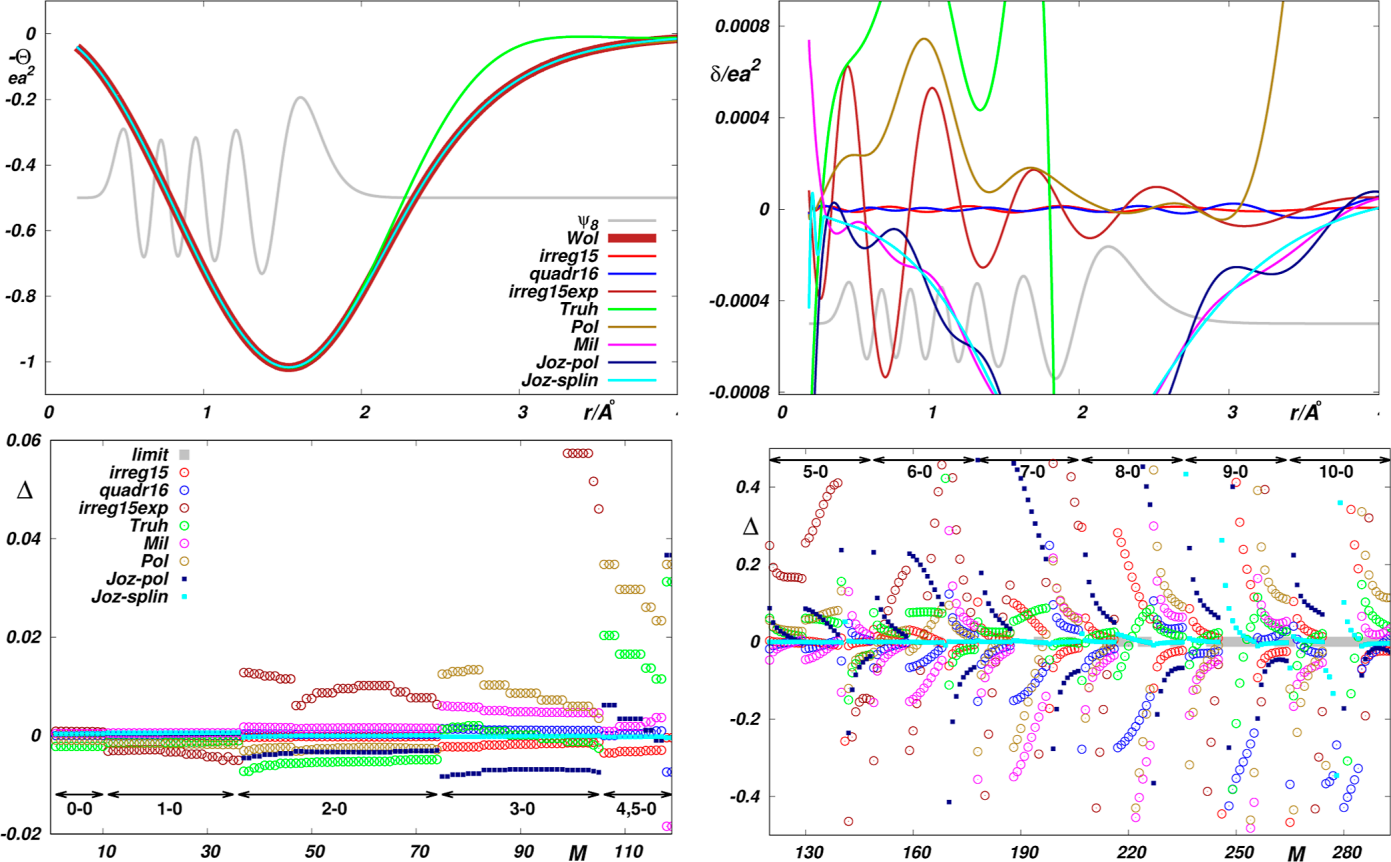
Upper panels: Empirical (analytic) EQM Θ(*r*) functions *irreg*15, *quadr*16 and *irreq*15*exp*^7^ and theoretical EQM Θ(*r*) functions *Wol*,^16^*Truh*,^23^*Pol*,^24^*Mil*^25^ and *Joz*(26) for H_2_ (left panel) and the deviations δ of these functions form the “reference” function *Wol* (right panel). Functions ψ_8_ and ψ_12_ are the wave functions of the vibrational states *v* = 8 and *v* = 12, respectively. Lower panels: Reproduction of the *Wol* reference data by the EQM functions probed in this study. Δ is defined by eq 7. *M* is the sequential number of the reference data (see Table S2 in the Supporting Information).

**Figure 2:**
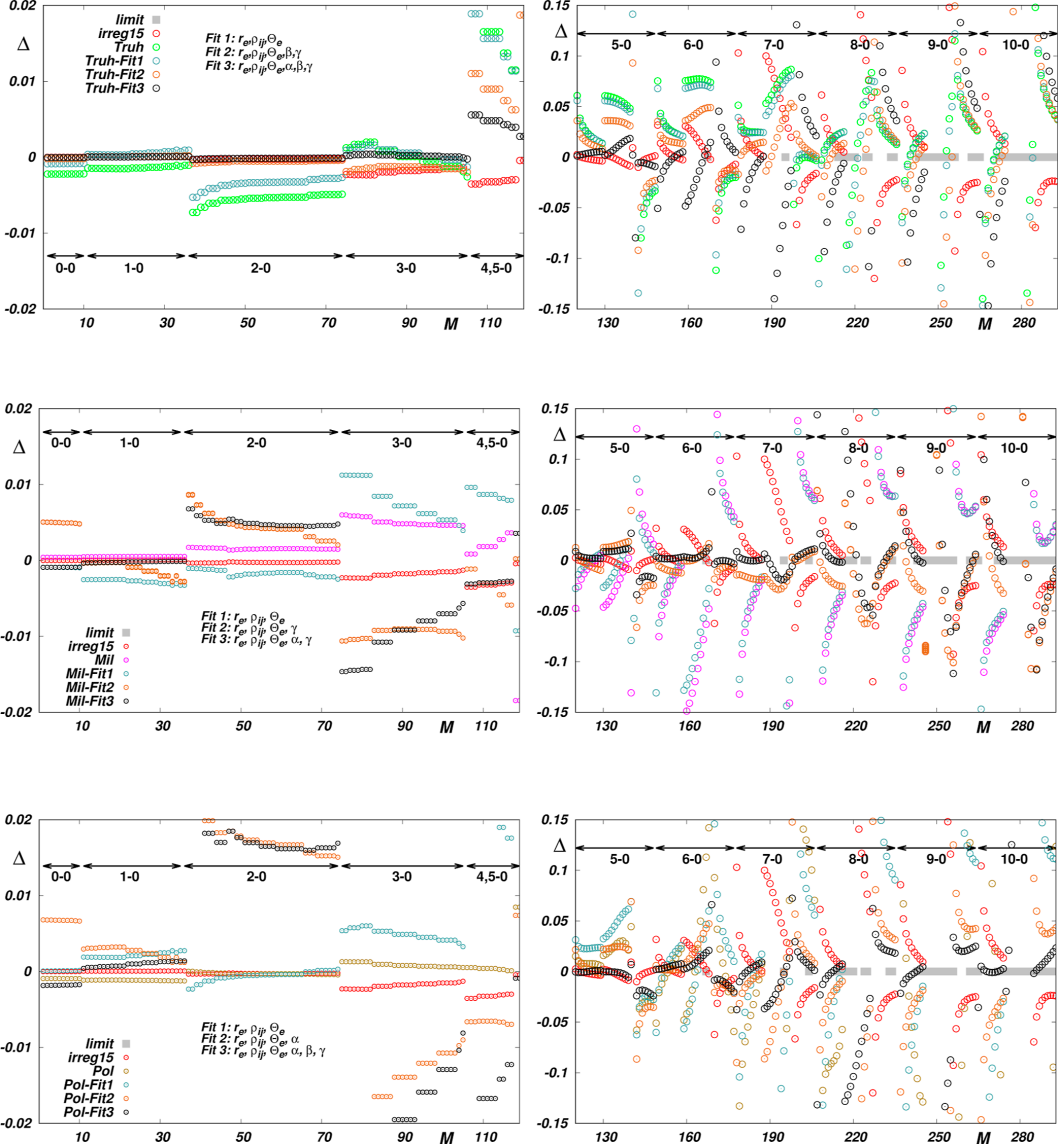
Reproduction of *Wol* reference data by morphed “less accurate” EQM functions *Truh*, *Mil* and *Pol*. The *limit* absolute value of ⟨*vJ*|Θ(*r*)|*v*^′^*J*^′^⟩ is less than 10^–5^. Δ is defined by eq 7. *M* is the sequential number of the reference data (see Table S2 in the Supporting Information).

**Figure 3:**
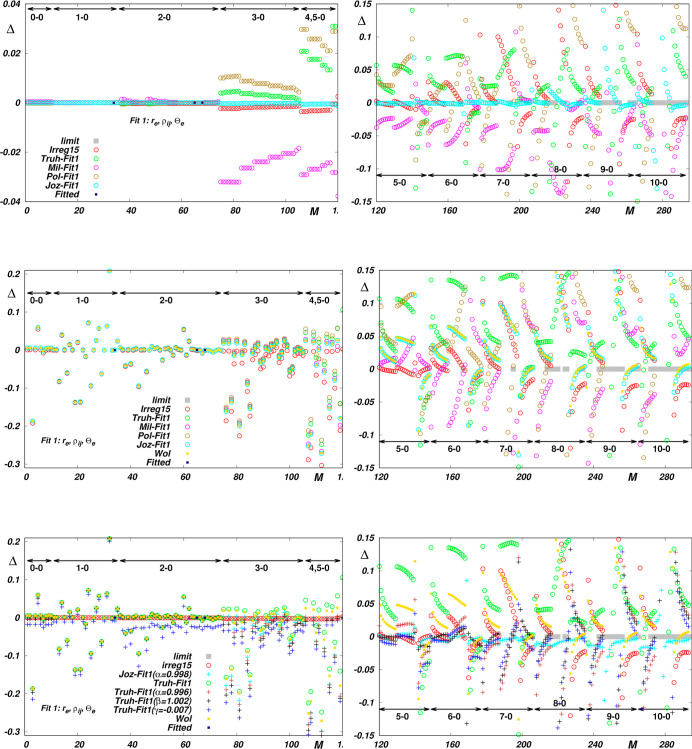
Reproduction of *Wol* reference data by morphed EQM functions obtained by fitting basic RRC parameters to theoretical reference values^16^ (top panels) and experimental reference values^1,20^ (lower panels) of ⟨*v* = 0,*J* = 2|Θ|*v*^′^ = 1,*J*^′^ = 4⟩, ⟨*v* = 0,*J* = 1|Θ|*v*^′^ = 2,*J*^′^ = 1⟩ and ⟨*v* = 0,*J* = 0|Θ|*v*^′^ = 2,*J*^′^ = 2⟩ only. The *limit* absolute value of ⟨*v*,*J*|Θ(*r*)|*v*^′^,*J*^′^⟩ is less than 10^–5^. Δ*i*s defined by eq 7. *M* is the sequential number of the reference data (see Tables S2 and S7 in the Supporting Information).

As seen in the top left panel of Figure 1, except for the dissociation asymptote of the EQM curve *Truh*, all the EQM curves visually coincide in the whole interval of vibrational distortions, where all the so far adequately studied states are fully localized (0.4 < *r* < 2 Å). Nevertheless, as seen in the top right panel of this figure, the dispersion of the probed curves exceeds the precision limit of the reference data (estimated to be 1 × 10^–5^ ea^2^ in ref (16)), and, although relatively small, these deviations give rise to significant discrepancies between corresponding transition moments for high overtones (see the bottom panels of Figure 1). In the bottom panels of Figure 1 it is also seen that the quality of reproduction of the fitted data depends not only on the accuracy of the fitted data, but also on the mathematical smoothness of the approximants used: The less the δ deviations oscillate, the better the reproduction is. Interpolating the EQM functions using splines appears to be more suitable than interpolating them using commonly used analytic functions. However, it has to be stressed that this only applies if the interpolation is done on a very dense grid of fitted data. In our case, as a matter of fact, only the *Wol* and *Joz* functions are adequately documented, and it is only the spline-interpolated *Joz* EQM function which reproduces the reference *Wol* data^16^ within the limits given by their relative precision of 10^–5^. As also seen in the bottom panels of Figure 1, the rest of the functions tested, including the best empirical *irreg*15 function, reproduce the reference data with visibly lower accuracy. However, as the functions exhibit correct topology, their morphing within the framework of Jenč’s RRC scheme appears to be worth attempting. As shown in Figure 2, the morphing of functions being probed can indeed improve the reproduction of reference data; however, this is mostly at the cost of including correction parameters. Although the number of parameters of morphed functions needed for a comparatively accurate description of the reference data, as provided by the best empirical function, is significantly lower than the number of parameters of this empirical function, it is possible that increasing the number of parameters strongly reduces the plausibility of the corresponding predictions. To achieve deeper insight and to assess the physical robustness of the morphing performed, two additional sets of fits of the three basic RRC parameters (*Fit*1: *r*_*e*_, ρ_*ij*_, *D*_*e*_) were made while considering only the three transition moments ⟨*v* = 0, *J* = 2|Θ|*v*^′^ = 1,*J*^′^ = 4⟩, ⟨*v* = 0,*J* = 1|Θ|*v*^′^ = 2,*J*^′^ = 1⟩ and ⟨*v* = 0,*J* = 0|Θ|*v*^′^ = 2,*J*^′^ = 2⟩: (i) fits to the transition moments fixed at their theoretical reference values (ref (16)), and (ii) fits to the transition moments fixed at their “best” experimental values (refs (20 and 21)). As seen in the top panels of Figure 3, the first fits predict the remaining reference data in close agreement with their original (see bottom panels of Figure 1) and “global” *Fit 1* (see Figure 2) counterparts, evidencing the excellent predictive robustness of the RRC approach taken. It is obvious that this approach allows the morphing of available EQM functions using as little as a single parameter, so it appears to be a suitable alternative where the amount of fitted data is strongly limited. However, one should take into account the fact that the fitted data depend on the fitted parameters nonlinearly (see e.g. Figure 4 of ref (11)) and that the predicted results may thus be strongly affected by a nonlinear spreading of experimental data errors. An illustration of the role of this fact in the present case can be obtained, for example, by comparing the top and middle panels of Figure 3. As one can see, a small, visually indistinguishable difference between the theoretical and experimental values of the fitted transition moments of the (1–0) and (2–0) vibrational bands (left panels) causes quite a marked change in the reproduction of transition moments of higher vibrational overtones (right panels). Fortunately, as can be seen in the lower panels of Figure 3, significant suppression of this problem can be achieved by considering even just one of the correction parameters (α, β, γ). This means, of course, that knowledge of at least of one additional data point is required. Apparently, from the point of view of the number of needed fitting parameters of the empirical EMG approximants based on the use of standard mathematical functions, it does not seem to be a limiting precondition.

**Figure 4:**
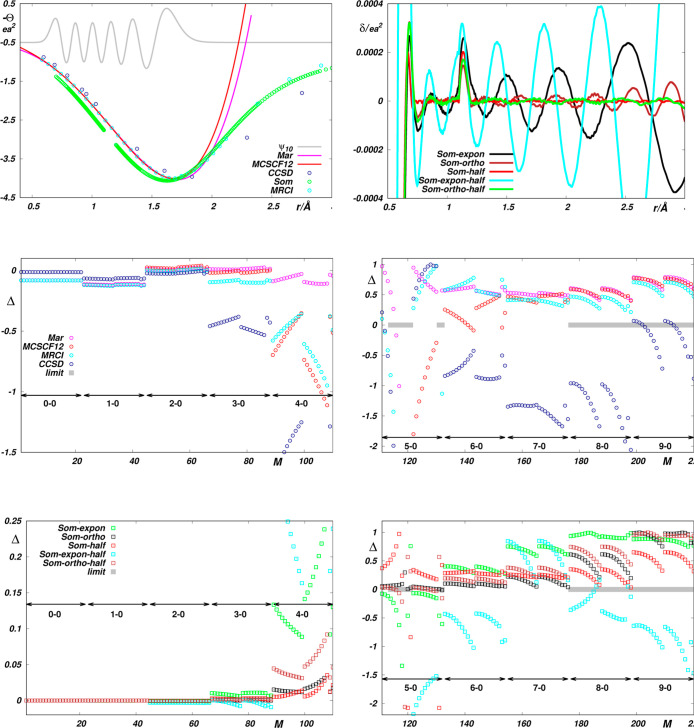
Top panels: Theoretical EQM Θ(*r*) functions *Som*,^2^*Mar*,^29^*MCSCF*12,^30^*MRCI*^31^ and *CCSD*(32) for HF taken from the literature; *Som-expon* and *Som-ortho* obtained by smoothing the original *Som* ab initio points using exponential and orthogonal polynomials; *Som-expon-half* and *Som-ortho-half* obtained by smoothing the *Som* ab initio points using exponential and orthogonal polynomials while discarding every second *Som* ab initio point; deviations δ of the functions *Som-expon*, *Som-ortho*, *Som-expon-half* and *Som-ortho-half* from the “reference” *Som* function. Function ψ_10_ is the wave function of the vibrational state *v* = 10. Middle panels: Reproduction of *Som* reference data by the functions probed. Δ*i*s defined by eq 7. *M* is the sequential number of the reference data (see Table S8 in the Supporting Information). The *limit* absolute value of ⟨*vJ*|Θ(*r*)|*v*^′^*J*^′^⟩ is less than 10^–4^. Bottom panels: Reproduction of *Som* reference data by functions obtained using exponential and orthogonal polynomials.

**Figure 5:**
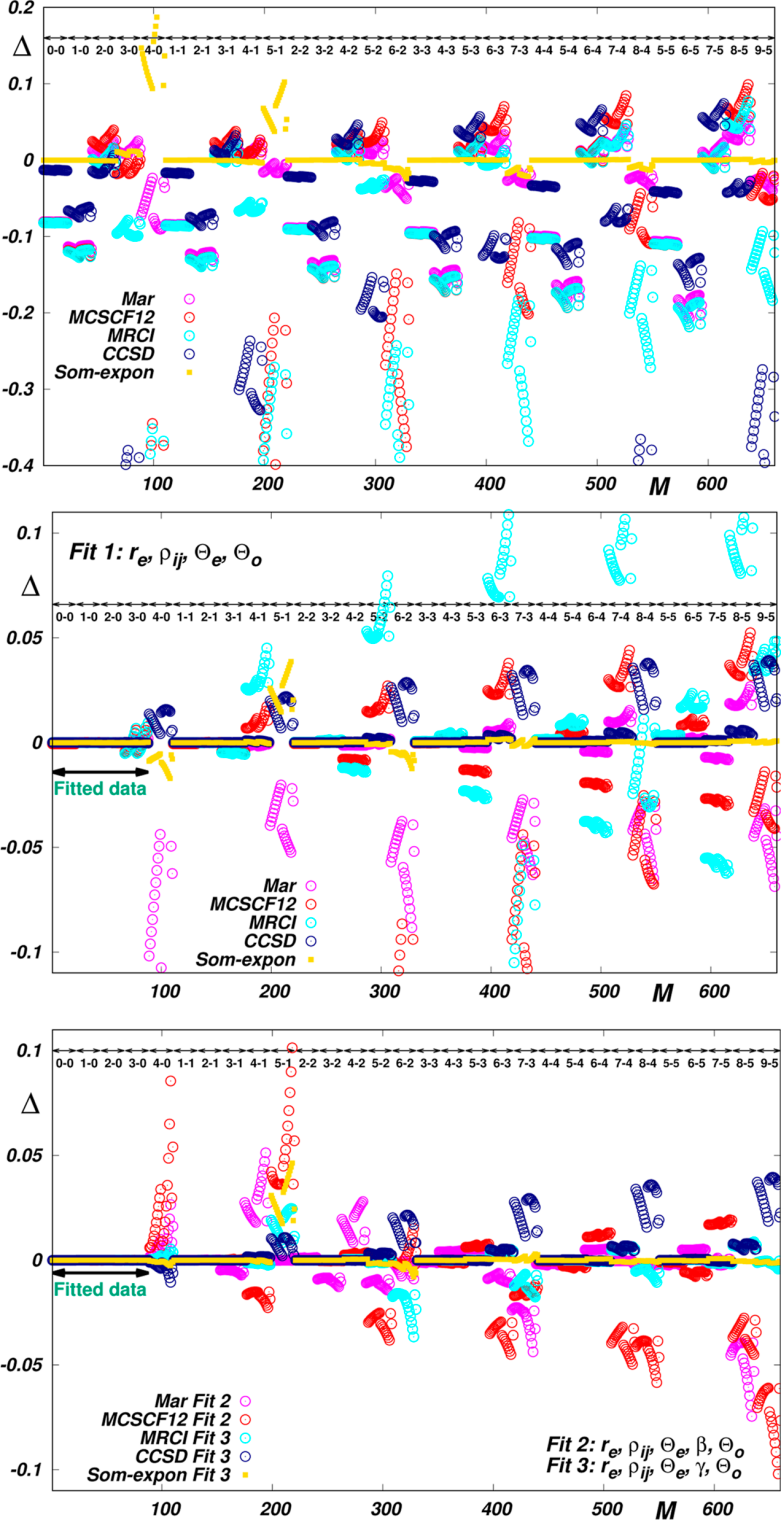
Reproduction of *Som* reference data by morphed EQM functions *Mar*, MCSCF12, *MRCI*, *CCSD* and *Som-expon*, obtained by fitting the ⟨*vJ*|Θ(*r*)|*v*^′^*J*^′^⟩ transition moments pertaining to the lower overtones [*v* – 0, *v* < 4]. Δ*i*s defined by eq 7. *M* is the sequential number of the reference data (see Table S9 in the Supporting Information).

In light of the results obtained for H_2_, the RRC approach appears to be fairly promising. It should be stressed, however, that they were acquired using strongly coinciding and very accurate EQM functions. Therefore, it seems worthwhile to explore the approach using less friendly models like, for example, HF, which proves to be highly suitable. It possesses a strong permanent electric quadrupole moment,^28^ and there are several theoretical EQM functions available for it in the literature (see refs (2, 29–32)). As is seen in the left top panel of Figure 4, though being of the same general shape, the functions do not coincide so closely as their H_2_ counterparts (see Figure 1), and only one of them, that is the function *Som*, is evaluated with a density of points which allows for a highly accurate spline interpolation. The spline-interpolated form of this function can therefore serve as a reference (similarly as the *Wol* function in the case of H_2_) for probing other functions at hand. In principle, vide infra, this probing confirms the previous findings obtained for H_2_. Most importantly, it evidence the insufficient flexibility of commonly used polynomial approximants for the accurate interpolation of theoretical EQM functions and, consequently, a need for much denser grids of theoretical points than those usually provided in the literature (see the right-hand top panel of Figure 4). Although the probed functions coincide with deviations which are much smaller than their actual values, the deviations give rise to fairly large discrepancies between the corresponding transition moments even for not very high overtones (see the middle and bottom panels of Figure 4). Nevertheless, as seen in Figure 5, a much more quantitative reproduction of the reference data can be achieved through morphing these functions by fitting such data within the framework of the RRC approach. The morphed approximants of the probed functions reproduce the reference data fairly quantitatively when using the “basic” RRC parameters [*r*_e_, ρ_*ij*_, Θ_e_, Θ_o_] only (see the middle panel of Figure 4), and the consideration of “correcting” parameters seems to improve the accuracy of predictions for Δ*v* > 3 data.

## 3 Conclusions

The concept of a universal reduced radial curve (RRC) enables the construction of few-parameter, physically correct electric quadrupole moment functions of diatomic molecules over a large range of vibrational distortions by employing standard quantum-chemical methods.

The probed RRC approach can be directly applied only to radial curves (and indeed to all their images obtained by translations, rigid rotations and linear scalings) with a single curvature extremum (like the usual potential energy curve). Unfortunately, the generalization of the approach to the case of curves with multiple curvature extrema and inflections does not appear to be straightforward.

## Data Availability

The data presented in this article are available upon request from the author.

## Supporting Information Available

Reference values of the line positions, line intensities, quadrupole transition moments, and the source of the H_2_ and HF reference data. Ab initio, reduced, and morphed EQM curves (PDF)
