# Supplementary material for: Reduced Radial Electric Quadrupole Moment Function for Diatomic Molecules
Source: J Chem Theory Comput. 2024 Dec 11;20(24):11005–12. doi: 10.1021/acs.jctc.4c01410 (PMC11672666; doi:10.1021/acs.jctc.4c01410)
Supplement: Supplementary file 1 — ct4c01410_si_001.pdf [file ct4c01410_si_001.pdf]

Supporting Information.
Reduced radial electric quadrupole moment function for diatomic molecules

Vladimir Špirko*

Institute of Organic Chemistry and Biochemistry, p.r.i.
Czech Academy of Sciences, Flemingovo nam.~2,
166 10 Prague 6, Czechia

E-mail: spirko@uochb.cas.cz

Phone: +420 220183 571

Table S1. Values of the line positions LP (in cm^{-1}, line intensities LI (in 10^{-28 cm/molecule), quadrupole transition moments <v"J"|Θ|v',J'> (in a.u.) and the source of the these H_2 reference data.

N	v"	J"	v'	J'	LP	LI	$\langle v''J'' \vert \Theta \vert v'J' \rangle$	Source^a
1	0	0	0	2	354.20617	2.02400	0.484805D+00	CKPK
2	0	1	0	3	586.75753	28.18000	0.486961D+00	CKPK
3	0	1	0	3	586.75753	19.80000	0.408184D+00	CKPK
4	0	2	0	4	814.04776	11.53000	0.490047D+00	CKPK
5	0	2	0	4	814.04776	12.90000	0.518344D+00	CKPK
6	0	3	0	5	1034.19941	16.98000	0.494402D+00	CKPK
7	0	3	0	5	1034.19941	16.70000	0.490309D+00	CKPK
8	0	3	0	5	1034.19941	16.94000	0.493819D+00	CKPK
9	0	4	0	6	1245.54240	1.30330	0.499552D+00	CKPK
10	0	5	0	7	1446.64796	0.46270	0.505771D+00	CKPK
11	0	4	1	4	4102.40256	0.83450	0.884649D-01	CKPK
12	0	3	1	3	4125.67843	19.20500	0.881836D-01	CKPK
13	0	3	1	3	4125.67843	16.37240	0.814211D-01	CKPK
14	0	3	1	3	4125.67843	20.80000	0.917725D-01	CKPK
15	0	2	1	2	4143.25993	27.21700	0.879714D-01	CKPK
16	0	2	1	2	4143.25993	24.18650	0.829292D-01	CKPK
17	0	2	1	2	4143.25993	27.98000	0.891959D-01	CKPK
18	0	1	1	1	4155.04087	217.00000	0.878263D-01	CKPK
19	0	1	1	1	4155.04087	167.44500	0.771491D-01	CKPK
20	0	1	1	1	4155.04087	225.90000	0.896092D-01	CKPK
21	0	1	1	1	4155.04087	167.80000	0.772308D-01	CKPK
22	0	0	1	2	4497.46609	107.81700	0.782057D-01	CKPK
23	0	0	1	2	4497.46609	104.18800	0.768782D-01	CKPK
24	0	0	1	2	4497.46609	125.03000	0.842174D-01	CKPK
25	0	0	1	2	4497.46609	90.00000	0.714522D-01	CKPK
26	0	1	1	3	4712.43596	318.50800	0.719290D-01	CKPK
27	0	1	1	3	4712.43596	334.89000	0.737556D-01	CKPK
28	0	1	1	3	4712.43596	358.00000	0.762580D-01	CKPK
29	0	1	1	3	4712.43596	282.00000	0.676812D-01	CKPK
30	0	2	1	4	4916.45031	45.71300	0.657416D-01	CKPK
31	0	2	1	4	4916.45031	52.09400	0.701802D-01	CKPK

32	0	2	1	4	4916.45031	72.90000	0.830203D-01	CKPK
33	0	3	1	5	5107.77031	29.76900	0.596421D-01	CKPK
34	0	2	1	4	4916.45031	45.72000	0.657467D-01	MBFKC
35	0	3	1	5	5107.77031	31.25640	0.611139D-01	CKPK
36	0	3	1	5	5107.77031	30.18000	0.600524D-01	CKPK
37	0	5	2	3	6982.64076	0.01098	0.885888D-02	CKPK
38	0	5	2	3	6982.64076	0.01010	0.849647D-02	CKPK
39	0	4	2	2	7237.57475	0.05797	0.948704D-02	CKPK
40	0	4	2	2	7237.57475	0.05390	0.914795D-02	CKPK
41	0	3	2	1	7488.16977	1.44140	0.100612D-01	CKPK
42	0	3	2	1	7488.16977	1.45000	0.100912D-01	CKPK
43	0	3	2	1	7488.16977	1.20000	0.918011D-02	CKPK
44	0	2	2	0	7732.40875	1.78790	0.105702D-01	CKPK
45	0	2	2	0	7732.40875	1.79000	0.105764D-01	CKPK
46	0	2	2	0	7732.40875	1.60000	0.999938D-02	CKPK
47	0	5	2	5	7913.73002	0.02364	0.117121D-01	CKPK
48	0	5	2	5	7913.73002	0.02410	0.118255D-01	CKPK
49	0	4	2	4	7970.78647	0.10491	0.115817D-01	KC
50	0	4	2	4	7970.78647	0.10534	0.116054D-01	FKKC
51	0	3	2	3	8016.84018	2.35300	0.113954D-01	CKPK
52	0	3	2	3	8016.84018	2.23300	0.111010D-01	CKPK
53	0	3	2	3	8016.84018	2.36710	0.114295D-01	KC
54	0	3	2	3	8016.84018	2.37590	0.114507D-01	FKKC
55	0	2	2	2	8051.62251	3.28900	0.112886D-01	CKPK
56	0	2	2	2	8051.62251	3.20000	0.111348D-01	CKPK
57	0	2	2	2	8051.62251	3.32740	0.113543D-01	KC
58	0	2	2	2	8051.62251	3.29820	0.113044D-01	FKKC
59	0	1	2	1	8074.92730	25.98000	0.112168D-01	CKPK
60	0	1	2	1	8074.92730	29.02380	0.118557D-01	CKPK
61	0	1	2	1	8074.92730	25.30000	0.110690D-01	CKPK
62	0	1	2	1	8074.92730	27.22000	0.114814D-01	CKPK
63	0	1	2	1	8074.92730	26.06900	0.112360D-01	KC
64	0	1	2	1	8074.92730	26.02100	0.112257D-01	FKKC
65	0	1	2	1	8074.92730	25.91500	0.112028D-01	FKKC
66	0	0	2	2	8405.82867	15.57400	0.116326D-01	CKPK
67	0	0	2	2	8405.82867	15.33000	0.115411D-01	CKPK
68	0	0	2	2	8405.82867	15.55400	0.116251D-01	FKKC
69	0	1	2	3	8603.59770	52.30100	0.118154D-01	CKPK
70	0	1	2	3	8603.59770	52.09400	0.117919D-01	CKPK
71	0	1	2	3	8603.59770	51.73000	0.117507D-01	CKPK
72	0	1	2	3	8603.59770	52.10000	0.117926D-01	CKPK
73	0	1	2	3	8603.59770	52.38000	0.118243D-01	FKKC
74	0	2	2	4	8784.83423	8.55700	0.119085D-01	CKPK
75	0	3	3	3	11678.11679	0.15110	0.164247D-02	CKPK
76	0	3	3	3	11678.11679	0.10800	0.138860D-02	CKPK
77	0	2	3	2	11729.84462	0.20930	0.161950D-02	CKPK
78	0	2	3	2	11729.84462	0.15600	0.139816D-02	CKPK
79	0	2	3	2	11729.84462	0.19600	0.156720D-02	TWCZLH
80	0	1	3	1	11764.49942	1.64300	0.160404D-02	CKPK
81	0	1	3	1	11764.49942	1.10000	0.131248D-02	CKPK
82	0	1	3	1	11764.49942	1.54400	0.155497D-02	TWCZLH
83	0	0	3	2	12084.05078	1.27600	0.193176D-02	CKPK

84	0	0	3	2	12084.05078	1.04200	0.174567D-02	CKPK
85	0	0	3	2	12084.05078	0.93000	0.164918D-02	CKPK
86	0	0	3	2	12084.05078	1.21900	0.188812D-02	TWCZLH
87	0	0	3	2	12084.05078	1.25200	0.191351D-02	HPCSLWCL
88	0	1	3	3	12264.87432	4.93600	0.213258D-02	CKPK
89	0	1	3	3	12264.87432	4.50000	0.203621D-02	CKPK
90	0	1	3	3	12264.87432	4.54000	0.204524D-02	CKPK
91	0	1	3	3	12264.87432	4.84000	0.211174D-02	CKPK
92	0	1	3	3	12264.87432	4.80700	0.210453D-02	TWCZLH
93	0	1	3	3	12264.87432	4.91100	0.212717D-02	HPCSLWCL
94	0	2	3	4	12423.66315	0.91420	0.231443D-02	CKPK
95	0	2	3	4	12423.66315	0.93000	0.233435D-02	CKPK
96	0	2	3	4	12423.66315	0.86000	0.224478D-02	CKPK
97	0	2	3	4	12423.66315	0.87900	0.226944D-02	TWCZLH
98	0	2	3	4	12423.66315	0.93900	0.234561D-02	HPCSLWCL
99	0	3	3	5	12558.92928	0.76210	0.247511D-02	CKPK
100	0	3	3	5	12558.92928	0.67000	0.232074D-02	CKPK
101	0	3	3	5	12558.92928	0.68000	0.233799D-02	CKPK
102	0	3	3	5	12558.92928	0.74400	0.244554D-02	TWCZLH
103	0	3	3	5	12558.92928	0.75100	0.245702D-02	HPCSLWCL
104	0	4	3	6	12669.47332	0.03700	0.259453D-02	TWCZLH
105	0	5	3	7	12754.40751	0.00900	0.269450D-02	TWCZLH
106	0	0	4	2	15535.02821	0.13480	0.430748D-03	CKPK
107	0	0	4	2	15535.02821	0.08186	0.335671D-03	CKPK
108	0	0	4	2	15535.02821	0.08600	0.344055D-03	CKPK
109	0	1	4	3	15699.02256	0.59550	0.511498D-03	CKPK
110	0	1	4	3	15699.02256	0.52800	0.481637D-03	CKPK
111	0	1	4	3	15699.02256	0.57300	0.501742D-03	CKPK
112	0	1	4	3	15699.02256	0.35300	0.393813D-03	CKPK
113	0	1	4	3	15699.02256	0.51000	0.473356D-03	CKPK
114	0	2	4	4	15835.50724	0.12270	0.589213D-03	CKPK
115	0	2	4	4	15835.50724	0.08900	0.501817D-03	CKPK
116	0	3	4	5	15943.10469	0.11180	0.662795D-03	CKPK
117	0	3	4	5	15943.10469	0.09300	0.604505D-03	CKPK
118	0	1	5	3	18906.73540	0.09521	0.154749D-03	CKPK
119	0	1	5	3	18906.73540	0.06500	0.127863D-03	CKPK

a

CKPK: Phys.Chem.Chem.Phys.2012,14,802-815.
FKKC: Phys.Chem.Chem.Phys.2023,25,14749-14756.
KC: J.Mol.Spectrosc.2014,300,55-59.
HPCSLWCL: ApJ2012,749:76.
TWCZLH:J.Mol.Spectrosc.2014,300,60-64.
MBFKC: Phys.Chem.Chem.Phys.2023,25,22662-22668,

Table S2. The reproduction of the quadrupole transition moments QTM-ai=<v"J"|Θ|v',J'> (in a.u.) evaluted using the ab initio quadrupole moment function Θ of Wolniewicz et al. (L.Wolniewicz,I.Subotin,A.Dalgarno:Astrophys.J.Suppl.Ser.35(1977)281-292) by its irreg15 approximant evaluated by Ushakov et al.

(Ushakov,S.A.Balashev,E.S.Medvedev:J.Mol.Spectrosc.399(2024)111863).
Δ=(QTM-ai - QTM-Irreg15)/QTM-ai.

M	v"	J"	v'	J'	QTM-ai	QTM-irreq15	ai-irreg15	Δ
1	0	0	0	2	0.484507D+00	0.484512D+00	-0.00000512	-0.0000
2	0	1	0	3	0.486629D+00	0.486634D+00	-0.00000505	-0.0000
3	0	1	0	3	0.486629D+00	0.486634D+00	-0.00000505	-0.0000
4	0	2	0	4	0.489801D+00	0.489806D+00	-0.00000493	-0.0000
5	0	2	0	4	0.489801D+00	0.489806D+00	-0.00000493	-0.0000
6	0	3	0	5	0.494013D+00	0.494018D+00	-0.00000475	-0.0000
7	0	3	0	5	0.494013D+00	0.494018D+00	-0.00000475	-0.0000
8	0	3	0	5	0.494013D+00	0.494018D+00	-0.00000475	-0.0000
9	0	4	0	6	0.499248D+00	0.499252D+00	-0.00000452	-0.0000
10	0	5	0	7	0.505488D+00	0.505492D+00	-0.00000420	-0.0000
11	0	4	1	4	0.884787D-01	0.884744D-01	0.00000425	0.0000
12	0	3	1	3	0.881988D-01	0.881949D-01	0.00000394	0.0000
13	0	3	1	3	0.881988D-01	0.881949D-01	0.00000394	0.0000
14	0	3	1	3	0.881988D-01	0.881949D-01	0.00000394	0.0000
15	0	2	1	2	0.879843D-01	0.879806D-01	0.00000370	0.0000
16	0	2	1	2	0.879843D-01	0.879806D-01	0.00000370	0.0000
17	0	2	1	2	0.879843D-01	0.879806D-01	0.00000370	0.0000
18	0	1	1	1	0.878390D-01	0.878355D-01	0.00000353	0.0000
19	0	1	1	1	0.878390D-01	0.878355D-01	0.00000353	0.0000
20	0	1	1	1	0.878390D-01	0.878355D-01	0.00000353	0.0000
21	0	1	1	1	0.878390D-01	0.878355D-01	0.00000353	0.0000
22	0	0	1	2	0.782162D-01	0.782125D-01	0.00000366	0.0000
23	0	0	1	2	0.782162D-01	0.782125D-01	0.00000366	0.0000
24	0	0	1	2	0.782162D-01	0.782125D-01	0.00000366	0.0000
25	0	0	1	2	0.782162D-01	0.782125D-01	0.00000366	0.0000
26	0	1	1	3	0.719404D-01	0.719365D-01	0.00000387	0.0001
27	0	1	1	3	0.719404D-01	0.719365D-01	0.00000387	0.0001
28	0	1	1	3	0.719404D-01	0.719365D-01	0.00000387	0.0001
29	0	1	1	3	0.719404D-01	0.719365D-01	0.00000387	0.0001
30	0	2	1	4	0.657543D-01	0.657501D-01	0.00000415	0.0001
31	0	2	1	4	0.657543D-01	0.657501D-01	0.00000415	0.0001
32	0	2	1	4	0.657543D-01	0.657501D-01	0.00000415	0.0001
33	0	3	1	5	0.596573D-01	0.596528D-01	0.00000449	0.0001
34	0	2	1	4	0.657543D-01	0.657501D-01	0.00000415	0.0001
35	0	3	1	5	0.596573D-01	0.596528D-01	0.00000449	0.0001
36	0	3	1	5	0.596573D-01	0.596528D-01	0.00000449	0.0001
37	0	5	2	3	0.885985D-02	0.886288D-02	-0.00000303	-0.0003
38	0	5	2	3	0.885985D-02	0.886288D-02	-0.00000303	-0.0003
39	0	4	2	2	0.948781D-02	0.949101D-02	-0.00000320	-0.0003
40	0	4	2	2	0.948781D-02	0.949101D-02	-0.00000320	-0.0003
41	0	3	2	1	0.100612D-01	0.100645D-01	-0.00000330	-0.0003
42	0	3	2	1	0.100612D-01	0.100645D-01	-0.00000330	-0.0003
43	0	3	2	1	0.100612D-01	0.100645D-01	-0.00000330	-0.0003
44	0	2	2	0	0.105701D-01	0.105734D-01	-0.00000334	-0.0003
45	0	2	2	0	0.105701D-01	0.105734D-01	-0.00000334	-0.0003
46	0	2	2	0	0.105701D-01	0.105734D-01	-0.00000334	-0.0003
47	0	5	2	5	0.117140D-01	0.117162D-01	-0.00000226	-0.0002

48	0	5	2	5	0.117140D-01	0.117162D-01	-0.00000226	-0.0002
49	0	4	2	4	0.115363D-01	0.115389D-01	-0.00000262	-0.0002
50	0	4	2	4	0.115363D-01	0.115389D-01	-0.00000262	-0.0002
51	0	3	2	3	0.113941D-01	0.113970D-01	-0.00000289	-0.0003
52	0	3	2	3	0.113941D-01	0.113970D-01	-0.00000289	-0.0003
53	0	3	2	3	0.113941D-01	0.113970D-01	-0.00000289	-0.0003
54	0	3	2	3	0.113941D-01	0.113970D-01	-0.00000289	-0.0003
55	0	2	2	2	0.112874D-01	0.112905D-01	-0.00000310	-0.0003
56	0	2	2	2	0.112874D-01	0.112905D-01	-0.00000310	-0.0003
57	0	2	2	2	0.112874D-01	0.112905D-01	-0.00000310	-0.0003
58	0	2	2	2	0.112874D-01	0.112905D-01	-0.00000310	-0.0003
59	0	1	2	1	0.112163D-01	0.112195D-01	-0.00000323	-0.0003
60	0	1	2	1	0.112163D-01	0.112195D-01	-0.00000323	-0.0003
61	0	1	2	1	0.112163D-01	0.112195D-01	-0.00000323	-0.0003
62	0	1	2	1	0.112163D-01	0.112195D-01	-0.00000323	-0.0003
63	0	1	2	1	0.112163D-01	0.112195D-01	-0.00000323	-0.0003
64	0	1	2	1	0.112163D-01	0.112195D-01	-0.00000323	-0.0003
65	0	1	2	1	0.112163D-01	0.112195D-01	-0.00000323	-0.0003
66	0	0	2	2	0.116321D-01	0.116352D-01	-0.00000305	-0.0003
67	0	0	2	2	0.116321D-01	0.116352D-01	-0.00000305	-0.0003
68	0	0	2	2	0.116321D-01	0.116352D-01	-0.00000305	-0.0003
69	0	1	2	3	0.118147D-01	0.118176D-01	-0.00000282	-0.0002
70	0	1	2	3	0.118147D-01	0.118176D-01	-0.00000282	-0.0002
71	0	1	2	3	0.118147D-01	0.118176D-01	-0.00000282	-0.0002
72	0	1	2	3	0.118147D-01	0.118176D-01	-0.00000282	-0.0002
73	0	1	2	3	0.118147D-01	0.118176D-01	-0.00000282	-0.0002
74	0	2	2	4	0.119082D-01	0.119107D-01	-0.00000251	-0.0002
75	0	3	3	3	0.164318D-02	0.164693D-02	-0.00000376	-0.0023
76	0	3	3	3	0.164318D-02	0.164693D-02	-0.00000376	-0.0023
77	0	2	3	2	0.162005D-02	0.162379D-02	-0.00000375	-0.0023
78	0	2	3	2	0.162005D-02	0.162379D-02	-0.00000375	-0.0023
79	0	2	3	2	0.162005D-02	0.162379D-02	-0.00000375	-0.0023
80	0	1	3	1	0.160464D-02	0.160837D-02	-0.00000373	-0.0023
81	0	1	3	1	0.160464D-02	0.160837D-02	-0.00000373	-0.0023
82	0	1	3	1	0.160464D-02	0.160837D-02	-0.00000373	-0.0023
83	0	0	3	2	0.193189D-02	0.193564D-02	-0.00000376	-0.0019
84	0	0	3	2	0.193189D-02	0.193564D-02	-0.00000376	-0.0019
85	0	0	3	2	0.193189D-02	0.193564D-02	-0.00000376	-0.0019
86	0	0	3	2	0.193189D-02	0.193564D-02	-0.00000376	-0.0019
87	0	0	3	2	0.193189D-02	0.193564D-02	-0.00000376	-0.0019
88	0	1	3	3	0.213291D-02	0.213668D-02	-0.00000377	-0.0018
89	0	1	3	3	0.213291D-02	0.213668D-02	-0.00000377	-0.0018
90	0	1	3	3	0.213291D-02	0.213668D-02	-0.00000377	-0.0018
91	0	1	3	3	0.213291D-02	0.213668D-02	-0.00000377	-0.0018
92	0	1	3	3	0.213291D-02	0.213668D-02	-0.00000377	-0.0018
93	0	1	3	3	0.213291D-02	0.213668D-02	-0.00000377	-0.0018
94	0	2	3	4	0.231477D-02	0.231853D-02	-0.00000376	-0.0016
95	0	2	3	4	0.231477D-02	0.231853D-02	-0.00000376	-0.0016
96	0	2	3	4	0.231477D-02	0.231853D-02	-0.00000376	-0.0016
97	0	2	3	4	0.231477D-02	0.231853D-02	-0.00000376	-0.0016
98	0	2	3	4	0.231477D-02	0.231853D-02	-0.00000376	-0.0016
99	0	3	3	5	0.247554D-02	0.247926D-02	-0.00000372	-0.0015

100	0	3	3	5	0.247554D-02	0.247926D-02	-0.00000372	-0.0015
101	0	3	3	5	0.247554D-02	0.247926D-02	-0.00000372	-0.0015
102	0	3	3	5	0.247554D-02	0.247926D-02	-0.00000372	-0.0015
103	0	3	3	5	0.247554D-02	0.247926D-02	-0.00000372	-0.0015
104	0	4	3	6	0.261382D-02	0.261746D-02	-0.00000364	-0.0014
105	0	5	3	7	0.272875D-02	0.273226D-02	-0.00000351	-0.0013
106	0	0	4	2	0.430959D-03	0.432484D-03	-0.00000152	-0.0035
107	0	0	4	2	0.430959D-03	0.432484D-03	-0.00000152	-0.0035
108	0	0	4	2	0.430959D-03	0.432484D-03	-0.00000152	-0.0035
109	0	1	4	3	0.511661D-03	0.513322D-03	-0.00000166	-0.0032
110	0	1	4	3	0.511661D-03	0.513322D-03	-0.00000166	-0.0032
111	0	1	4	3	0.511661D-03	0.513322D-03	-0.00000166	-0.0032
112	0	1	4	3	0.511661D-03	0.513322D-03	-0.00000166	-0.0032
113	0	1	4	3	0.511661D-03	0.513322D-03	-0.00000166	-0.0032
114	0	2	4	4	0.589397D-03	0.591211D-03	-0.00000181	-0.0031
115	0	2	4	4	0.589397D-03	0.591211D-03	-0.00000181	-0.0031
116	0	3	4	5	0.663104D-03	0.665081D-03	-0.00000198	-0.0030
117	0	3	4	5	0.663104D-03	0.665081D-03	-0.00000198	-0.0030
118	0	1	5	3	0.154807D-03	0.154875D-03	-0.00000007	-0.0004
119	0	1	5	3	0.154807D-03	0.154875D-03	-0.00000007	-0.0004
120	0	0	5	0	-0.732214D-04	-0.730333D-04	-0.00000019	0.0026
121	0	0	5	2	-0.121891D-03	-0.121842D-03	-0.00000005	0.0004
122	0	1	5	3	-0.154807D-03	-0.154875D-03	0.00000007	-0.0004
123	0	2	5	4	-0.187680D-03	-0.187889D-03	0.00000021	-0.0011
124	0	3	5	5	-0.220033D-03	-0.220403D-03	0.00000037	-0.0017
125	0	4	5	6	-0.251419D-03	-0.251969D-03	0.00000055	-0.0022
126	0	5	5	7	-0.281444D-03	-0.282188D-03	0.00000074	-0.0026
127	0	6	5	8	-0.309782D-03	-0.310728D-03	0.00000095	-0.0031
128	0	7	5	9	-0.336183D-03	-0.337331D-03	0.00000115	-0.0034
129	0	8	5	10	-0.360473D-03	-0.361811D-03	0.00000134	-0.0037
130	0	1	5	1	-0.739248D-04	-0.737596D-04	-0.00000017	0.0022
131	0	2	5	2	-0.753295D-04	-0.752102D-04	-0.00000012	0.0016
132	0	3	5	3	-0.774320D-04	-0.773815D-04	-0.00000005	0.0007
133	0	4	5	4	-0.802289D-04	-0.802698D-04	0.00000004	-0.0005
134	0	5	5	5	-0.837185D-04	-0.838728D-04	0.00000015	-0.0018
135	0	6	5	6	-0.879033D-04	-0.881920D-04	0.00000029	-0.0033
136	0	7	5	7	-0.927926D-04	-0.932344D-04	0.00000044	-0.0048
137	0	8	5	8	-0.984056D-04	-0.990162D-04	0.00000061	-0.0062
138	0	9	5	9	-0.104774D-03	-0.105565D-03	0.00000079	-0.0075
139	0	10	5	10	-0.111947D-03	-0.112922D-03	0.00000098	-0.0087
140	0	2	5	0	-0.275580D-04	-0.273015D-04	-0.00000026	0.0093
141	0	3	5	1	0.108451D-05	0.136321D-05	-0.00000028	-0.2570
142	0	4	5	2	0.278518D-04	0.281300D-04	-0.00000028	-0.0100
143	0	5	5	3	0.526001D-04	0.528552D-04	-0.00000026	-0.0049
144	0	6	5	4	0.752813D-04	0.754912D-04	-0.00000021	-0.0028
145	0	7	5	5	0.959357D-04	0.960781D-04	-0.00000014	-0.0015
146	0	8	5	6	0.114679D-03	0.114732D-03	-0.00000005	-0.0005
147	0	9	5	7	0.131687D-03	0.131628D-03	0.00000006	0.0004
148	0	10	5	8	0.147183D-03	0.146991D-03	0.00000019	0.0013
149	0	0	6	0	-0.197802D-04	-0.191528D-04	-0.00000063	0.0317
150	0	0	6	2	-0.408125D-04	-0.402500D-04	-0.00000056	0.0138
151	0	1	6	3	-0.555132D-04	-0.550100D-04	-0.00000050	0.0091

152	0	2	6	4	-0.705797D-04	-0.701530D-04	-0.00000043	0.0060
153	0	3	6	5	-0.857980D-04	-0.854662D-04	-0.00000033	0.0039
154	0	4	6	6	-0.100958D-03	-0.100740D-03	-0.00000022	0.0022
155	0	5	6	7	-0.115864D-03	-0.115779D-03	-0.00000008	0.0007
156	0	6	6	8	-0.130350D-03	-0.130416D-03	0.00000007	-0.0005
157	0	7	6	9	-0.144280D-03	-0.144511D-03	0.00000023	-0.0016
158	0	8	6	10	-0.157554D-03	-0.157961D-03	0.00000041	-0.0026
159	0	1	6	1	-0.200682D-04	-0.194514D-04	-0.00000062	0.0307
160	0	2	6	2	-0.206430D-04	-0.200479D-04	-0.00000060	0.0288
161	0	3	6	3	-0.215031D-04	-0.209413D-04	-0.00000056	0.0261
162	0	4	6	4	-0.226465D-04	-0.221308D-04	-0.00000052	0.0228
163	0	5	6	5	-0.240725D-04	-0.236166D-04	-0.00000046	0.0189
164	0	6	6	6	-0.257818D-04	-0.254008D-04	-0.00000038	0.0148
165	0	7	6	7	-0.277788D-04	-0.274885D-04	-0.00000029	0.0104
166	0	8	6	8	-0.300720D-04	-0.298887D-04	-0.00000018	0.0061
167	0	9	6	9	-0.326764D-04	-0.326163D-04	-0.00000006	0.0018
168	0	10	6	10	-0.356145D-04	-0.356929D-04	0.00000008	-0.0022
169	0	2	6	0	-0.820220D-06	-0.164618D-06	-0.00000066	0.7993
170	0	3	6	1	0.106609D-04	0.113244D-04	-0.00000066	-0.0622
171	0	4	6	2	0.210834D-04	0.217453D-04	-0.00000066	-0.0314
172	0	5	6	3	0.304357D-04	0.310869D-04	-0.00000065	-0.0214
173	0	6	6	4	0.387480D-04	0.393793D-04	-0.00000063	-0.0163
174	0	7	6	5	0.460857D-04	0.466873D-04	-0.00000060	-0.0131
175	0	8	6	6	0.525413D-04	0.531029D-04	-0.00000056	-0.0107
176	0	9	6	7	0.582270D-04	0.587367D-04	-0.00000051	-0.0088
177	0	10	6	8	0.632674D-04	0.637118D-04	-0.00000044	-0.0070
178	0	0	7	0	-0.548270D-05	-0.491831D-05	-0.00000056	0.1029
179	0	0	7	2	-0.155098D-04	-0.149574D-04	-0.00000055	0.0356
180	0	1	7	3	-0.227027D-04	-0.221666D-04	-0.00000054	0.0236
181	0	2	7	4	-0.302260D-04	-0.297159D-04	-0.00000051	0.0169
182	0	3	7	5	-0.379802D-04	-0.375079D-04	-0.00000047	0.0124
183	0	4	7	6	-0.458634D-04	-0.454430D-04	-0.00000042	0.0092
184	0	5	7	7	-0.537788D-04	-0.534258D-04	-0.00000035	0.0066
185	0	6	7	8	-0.616401D-04	-0.613714D-04	-0.00000027	0.0044
186	0	7	7	9	-0.693768D-04	-0.692093D-04	-0.00000017	0.0024
187	0	8	7	10	-0.769366D-04	-0.768867D-04	-0.00000005	0.0006
188	0	1	7	1	-0.561635D-05	-0.505474D-05	-0.00000056	0.1000
189	0	2	7	2	-0.588318D-05	-0.532751D-05	-0.00000056	0.0945
190	0	3	7	3	-0.628239D-05	-0.573658D-05	-0.00000055	0.0869
191	0	4	7	4	-0.681321D-05	-0.628226D-05	-0.00000053	0.0779
192	0	5	7	5	-0.747536D-05	-0.696562D-05	-0.00000051	0.0682
193	0	6	7	6	-0.826961D-05	-0.778899D-05	-0.00000048	0.0581
194	0	7	7	7	-0.919848D-05	-0.875653D-05	-0.00000044	0.0480
195	0	8	7	8	-0.102670D-04	-0.987489D-05	-0.00000039	0.0382
196	0	9	7	9	-0.114836D-04	-0.111539D-04	-0.00000033	0.0287
197	0	10	7	10	-0.128610D-04	-0.126076D-04	-0.00000025	0.0197
198	0	2	7	0	0.326985D-05	0.383361D-05	-0.00000056	-0.1724
199	0	3	7	1	0.842121D-05	0.898172D-05	-0.00000056	-0.0666
200	0	4	7	2	0.129883D-04	0.135439D-04	-0.00000056	-0.0428
201	0	5	7	3	0.169874D-04	0.175367D-04	-0.00000055	-0.0323
202	0	6	7	4	0.204537D-04	0.209953D-04	-0.00000054	-0.0265
203	0	7	7	5	0.234371D-04	0.239694D-04	-0.00000053	-0.0227

204	0	8	7	6	0.259981D-04	0.265184D-04	-0.00000052	-0.0200
205	0	9	7	7	0.282031D-04	0.287077D-04	-0.00000050	-0.0179
206	0	10	7	8	0.301216D-04	0.306052D-04	-0.00000048	-0.0161
207	0	0	8	0	-0.130813D-05	-0.920770D-06	-0.00000039	0.2961
208	0	0	8	2	-0.652083D-05	-0.612060D-05	-0.00000040	0.0614
209	0	1	8	3	-0.103413D-04	-0.993558D-05	-0.00000041	0.0392
210	0	2	8	4	-0.144050D-04	-0.139983D-04	-0.00000041	0.0282
211	0	3	8	5	-0.186641D-04	-0.182627D-04	-0.00000040	0.0215
212	0	4	8	6	-0.230674D-04	-0.226799D-04	-0.00000039	0.0168
213	0	5	8	7	-0.275652D-04	-0.272022D-04	-0.00000036	0.0132
214	0	6	8	8	-0.321125D-04	-0.317867D-04	-0.00000033	0.0101
215	0	7	8	9	-0.366724D-04	-0.363978D-04	-0.00000027	0.0075
216	0	8	8	10	-0.412179D-04	-0.410098D-04	-0.00000021	0.0050
217	0	1	8	1	-0.137660D-05	-0.988428D-06	-0.00000039	0.2820
218	0	2	8	2	-0.151333D-05	-0.112381D-05	-0.00000039	0.2574
219	0	3	8	3	-0.171797D-05	-0.132713D-05	-0.00000039	0.2275
220	0	4	8	4	-0.199025D-05	-0.159893D-05	-0.00000039	0.1966
221	0	5	8	5	-0.233021D-05	-0.194035D-05	-0.00000039	0.1673
222	0	6	8	6	-0.273857D-05	-0.235339D-05	-0.00000039	0.1407
223	0	7	8	7	-0.321708D-05	-0.284130D-05	-0.00000038	0.1168
224	0	8	8	8	-0.376904D-05	-0.340902D-05	-0.00000036	0.0955
225	0	9	8	9	-0.439981D-05	-0.406359D-05	-0.00000034	0.0764
226	0	10	8	10	-0.511747D-05	-0.481476D-05	-0.00000030	0.0592
227	0	2	8	0	0.312131D-05	0.349547D-05	-0.00000037	-0.1199
228	0	3	8	1	0.566722D-05	0.603352D-05	-0.00000037	-0.0646
229	0	4	8	2	0.788035D-05	0.824044D-05	-0.00000036	-0.0457
230	0	5	8	3	0.977924D-05	0.101350D-04	-0.00000036	-0.0364
231	0	6	8	4	0.113915D-04	0.117448D-04	-0.00000035	-0.0310
232	0	7	8	5	0.127512D-04	0.131036D-04	-0.00000035	-0.0276
233	0	8	8	6	0.138965D-04	0.142489D-04	-0.00000035	-0.0254
234	0	9	8	7	0.148674D-04	0.152200D-04	-0.00000035	-0.0237
235	0	10	8	8	0.157041D-04	0.160559D-04	-0.00000035	-0.0224
236	0	0	9	0	-0.589779D-07	-0.176285D-06	0.00000012	-1.9890
237	0	0	9	2	-0.298232D-05	-0.272667D-05	-0.00000026	0.0857
238	0	1	9	3	-0.516420D-05	-0.489528D-05	-0.00000027	0.0521
239	0	2	9	4	-0.751855D-05	-0.723766D-05	-0.00000028	0.0374
240	0	3	9	5	-0.100214D-04	-0.973143D-05	-0.00000029	0.0289
241	0	4	9	6	-0.126464D-04	-0.123519D-04	-0.00000029	0.0233
242	0	5	9	7	-0.153671D-04	-0.150746D-04	-0.00000029	0.0190
243	0	6	9	8	-0.181592D-04	-0.178772D-04	-0.00000028	0.0155
244	0	7	9	9	-0.210026D-04	-0.207415D-04	-0.00000026	0.0124
245	0	8	9	10	-0.238828D-04	-0.236545D-04	-0.00000023	0.0096
246	0	1	9	1	-0.970439D-07	-0.140234D-06	0.00000004	-0.4451
247	0	2	9	2	-0.173089D-06	-0.680388D-07	-0.00000011	0.6069
248	0	3	9	3	-0.286997D-06	-0.405410D-07	-0.00000025	0.8587
249	0	4	9	4	-0.438729D-06	-0.186013D-06	-0.00000025	0.5760
250	0	5	9	5	-0.628485D-06	-0.369314D-06	-0.00000026	0.4124
251	0	6	9	6	-0.856910D-06	-0.592021D-06	-0.00000026	0.3091
252	0	7	9	7	-0.112534D-05	-0.856596D-06	-0.00000027	0.2388
253	0	8	9	8	-0.143611D-05	-0.116668D-05	-0.00000027	0.1876
254	0	9	9	9	-0.179289D-05	-0.152742D-05	-0.00000027	0.1481
255	0	10	9	10	-0.220114D-05	-0.194586D-05	-0.00000026	0.1160

256	0	2	9	0	0.236917D-05	0.258871D-05	-0.00000022	-0.0927
257	0	3	9	1	0.373723D-05	0.394853D-05	-0.00000021	-0.0565
258	0	4	9	2	0.490706D-05	0.511272D-05	-0.00000021	-0.0419
259	0	5	9	3	0.589406D-05	0.609678D-05	-0.00000020	-0.0344
260	0	6	9	4	0.671821D-05	0.692062D-05	-0.00000020	-0.0301
261	0	7	9	5	0.740242D-05	0.760693D-05	-0.00000020	-0.0276
262	0	8	9	6	0.797114D-05	0.817979D-05	-0.00000021	-0.0262
263	0	9	9	7	0.844917D-05	0.866348D-05	-0.00000021	-0.0254
264	0	10	9	8	0.886084D-05	0.908164D-05	-0.00000022	-0.0249
265	0	0	10	0	0.284932D-06	0.417965D-06	-0.00000013	-0.4669
266	0	0	10	2	-0.146685D-05	-0.131387D-05	-0.00000015	0.1043
267	0	1	10	3	-0.279466D-05	-0.262741D-05	-0.00000017	0.0598
268	0	2	10	4	-0.424498D-05	-0.406321D-05	-0.00000018	0.0428
269	0	3	10	5	-0.580546D-05	-0.561001D-05	-0.00000020	0.0337
270	0	4	10	6	-0.746171D-05	-0.725475D-05	-0.00000021	0.0277
271	0	5	10	7	-0.919870D-05	-0.898394D-05	-0.00000021	0.0233
272	0	6	10	8	-0.110019D-04	-0.107848D-04	-0.00000022	0.0197
273	0	7	10	9	-0.128584D-04	-0.126462D-04	-0.00000021	0.0165
274	0	8	10	10	-0.147563D-04	-0.145581D-04	-0.00000020	0.0134
275	0	1	10	1	0.262238D-06	0.397415D-06	-0.00000014	-0.5155
276	0	2	10	2	0.216868D-06	0.356232D-06	-0.00000014	-0.6426
277	0	3	10	3	0.148830D-06	0.294207D-06	-0.00000015	-0.9768
278	0	4	10	4	0.580426D-07	0.210929D-06	-0.00000015	-2.6340
279	0	5	10	5	-0.557542D-07	-0.105666D-06	0.00000005	-0.8952
280	0	6	10	6	-0.193134D-06	-0.227816D-07	-0.00000017	0.8820
281	0	7	10	7	-0.355140D-06	-0.176256D-06	-0.00000018	0.5037
282	0	8	10	8	-0.543474D-06	-0.357445D-06	-0.00000019	0.3423
283	0	9	10	9	-0.760705D-06	-0.570108D-06	-0.00000019	0.2506
284	0	10	10	10	-0.101051D-05	-0.819299D-06	-0.00000019	0.1892
285	0	2	10	0	0.171216D-05	0.183122D-05	-0.00000012	-0.0695
286	0	3	10	1	0.250291D-05	0.261508D-05	-0.00000011	-0.0448
287	0	4	10	2	0.316992D-05	0.327772D-05	-0.00000011	-0.0340
288	0	5	10	3	0.372503D-05	0.383100D-05	-0.00000011	-0.0284
289	0	6	10	4	0.418246D-05	0.428907D-05	-0.00000011	-0.0255
290	0	7	10	5	0.455776D-05	0.466732D-05	-0.00000011	-0.0240
291	0	8	10	6	0.486699D-05	0.498157D-05	-0.00000011	-0.0235
292	0	9	10	7	0.512599D-05	0.524734D-05	-0.00000012	-0.0237

Table S3. Smoothing the electric quadrupole moment function EQMF of [Truhlar D.G.: Int.J.Quantum Chem. 1972,6,975-988].

r/ANG	AB INITIO	CALCULATED	AI-C	WEIGHT
0.0000000	0.00000000	-0.02174221	0.02174221	0.00
0.3175063	0.19985000	0.19983877	0.00001123	1.00
0.4233418	0.34283000	0.34290585	-0.00007585	1.00
0.5291772	0.51411000	0.51399803	0.00011197	1.00
0.6350127	0.70712000	0.70708285	0.00003715	1.00
0.7143893	0.86203000	0.86203373	-0.00000373	1.00
0.7408481	0.91490000	0.91495475	-0.00005475	1.00

0.7673070	0.96822000	0.96829122	-0.00007122	1.00
0.8466836	1.12920000	1.12940998	-0.00020998	1.00
0.9525190	1.34200000	1.34142917	0.00057083	1.00
1.0583545	1.54100000	1.54139308	-0.00039308	1.00
1.1641899	1.71930000	1.71938188	-0.00008188	1.00
1.2700254	1.86610000	1.86561677	0.00048323	1.00
1.3758608	1.97040000	1.97108384	-0.00068384	1.00
1.4816963	2.02890000	2.02833097	0.00056903	1.00
1.5875317	2.03210000	2.03235807	-0.00025807	1.00
1.6933672	1.98150000	1.98145105	0.00004895	1.00
5.2917725	0.00000000	0.00000000	-0.00000000	1.00

SUM OF SQUARES OF ERRORS=0.1653D-05 AND THE STANDARD DEVIATION IS=0.0004

	OLD PARM	NEW PARM	DELTA PARM	ST. ERR.
r_e	0.154252039D+01	0.154252039D+01	0.73275D-14	0.36361D-03
C[3]	-0.760720466D+01	-0.760720466D+01	-0.13687D-11	0.39707D-01
C[4]	0.144373140D+01	0.144373140D+01	-0.11459D-10	0.29637D+00
C[5]	0.503123381D+01	0.503123381D+01	-0.23637D-10	0.63490D+00
C[6]	0.323049829D+01	0.323049829D+01	0.38392D-11	0.14319D+00
C[7]	-0.253805163D+01	-0.253805163D+01	0.32822D-10	0.88464D+00
V0	0.203734299D+01	0.203734299D+01	0.88818D-14	0.29700D-03

(a=0.5679, a1=0.016, a2=0.2165 fixed after a preliminary determination)

```
y=1-exp{a*(r-r_e)+a1*(r-r_e)+a2*(r-r_e)^2
EQMF=V0+C[3]*y^2+C[4]*y^3+C[5]*y^4+C[6]*y^5+C[7]*y^6
```

Table S4. Smoothing the electric quadrupole moment function EQMF of [Poll J.D., Wolniewicz L.: J.Chem.Phys. 1978,68,3053-3058].

r/ANG	AB INITIO	CALCULATED	AI-C	WEIGHT
0.1058354	0.011709	0.011742	-0.000033	33.09
0.1322943	0.018199	0.018202	-0.000003	33.02
0.1587532	0.026052	0.026035	0.000017	32.94
0.1852120	0.035231	0.035206	0.000025	32.84
0.2116709	0.045699	0.045680	0.000019	32.73
0.2381298	0.057417	0.057416	0.000001	32.60
0.2645886	0.070343	0.070370	-0.000027	32.46
0.2910475	0.084436	0.084497	-0.000061	32.32
0.3175063	0.099778	0.099751	0.000027	321.57
0.4233418	0.171026	0.171070	-0.000043	314.36
0.5291772	0.256540	0.256542	-0.000002	306.13
0.6350127	0.353023	0.352967	0.000056	297.35
0.7408481	0.456844	0.456829	0.000015	288.45
0.8466836	0.564004	0.564086	-0.000083	279.80
0.9525190	0.670111	0.670181	-0.000069	271.73

1.0583545	0.770409	0.770242	0.000166	264.52
1.1641899	0.859860	0.859308	0.000552	258.41
1.1641899	0.858830	0.859308	-0.000478	258.48
1.2700254	0.932286	0.932485	-0.000198	253.66
1.3758608	0.985130	0.985166	-0.000036	250.30
1.4816963	1.013575	1.013481	0.000094	248.54
1.5875317	1.015040	1.014985	0.000055	248.44
1.7992026	0.938830	0.938864	-0.000034	253.24
1.8521204	0.905587	0.905626	-0.000039	255.39
2.1167090	0.690610	0.690595	0.000015	270.23
2.2225444	0.596956	0.596947	0.000009	277.24
2.3283799	0.507086	0.507069	0.000017	284.33
2.4342153	0.424391	0.424386	0.000005	291.17
2.5400508	0.350787	0.350803	-0.000016	297.55
2.6458862	0.286972	0.286998	-0.000026	303.31
2.8575571	0.187510	0.187482	0.000027	312.74
3.0692280	0.119732	0.119740	-0.000008	319.52
3.1750635	0.095098	0.095285	-0.000187	0.10

SUM OF SQUARES OF ERRORS=0.1653D-05 AND THE STANDARD DEVIATION IS=0.0027

	OLD PARM	NEW PARM	DELTA PARM	ST. ERR.
r_e	0.154046602D+01	0.154046602D+01	-0.22204D-15	0.22007D-03
C[3]	0.208075473D+01	-0.208075473D+01	-0.35527D-14	0.23081D-02
C[4]	-0.391360790D+00	-0.391360790D+00	0.14599D-13	0.99237D-02
C[5]	0.124518162D+01	0.124518162D+01	0.23093D-13	0.13381D-01
C[6]	0.148823105D+01	0.148823105D+01	-0.89928D-13	0.56235D-01
C[7]	-0.444778269D+00	-0.444778269D+00	-0.44464D-13	0.23255D-01
C[8]	-0.153452925D+01	-0.153452925D+01	0.18741D-12	0.11337D+00
C[10]	0.747444894D+00	0.747444894D+00	-0.12945D-12	0.77380D-01
C[11]	-0.134977216D+00	-0.134977216D+00	0.40801D-13	0.19387D-01
V0	0.101770250D+01	0.101770250D+01	0.22204D-15	0.97662D-04

(a=0.7679117, a1=0.0, a2=0.2491833 fixed after a preliminary determination)

y=1-exp{a*(r-r_e)+a1*(r-r_e)+a2*(r-r_e)^2

EQMF=V0+C[3]*y^2+C[4]*y^3+C[5]*y^4+C[6]*y^5+C[7]*y^6+C[8]*y^7+C[10]*y^9+C[11]*y^10

Table S5. Smoothing the electric quadrupole moment function EQMF of [Miliordos E., Hunt K.L.C.: J.Chem.Phys. 2028,149,1.5066308].

r/ANG	AB INITIO	CALCULATED	AI-C	WEIGHT
0.3000435	0.179000	0.178995	0.000005	31.36
0.4984850	0.460400	0.460371	0.000029	28.82

```
  0.5879159      0.617200      0.617217     -0.000017   275.69
  0.6773469      0.786800      0.786782      0.000018   263.38
  0.7667778      0.964600      0.964596      0.000004   251.60
  0.9456397      1.324800      1.324822     -0.000022   230.69
  1.1245017      1.653200      1.653180      0.000020   214.45
  1.3033636      1.901400      1.901405     -0.000005   203.61
  1.4822255      2.025200      2.025175      0.000025   198.60
  1.5875317      2.027600      2.027670     -0.000070   198.51
  1.6933672      1.976200      1.976155      0.000045   200.55
  1.7992026      1.875000      1.875011     -0.000011   204.71
  1.9050381      1.733400      1.733378      0.000022   210.82
  2.0108735      1.563600      1.563546      0.000054   218.65
  2.1167090      1.378800      1.378883     -0.000083   227.85
  2.2490033      1.145800      1.145859     -0.000059   240.63
  2.3812976      0.927600      0.927493      0.000107   253.96
  2.6458862      0.572600      0.572627     -0.000027   279.13
  2.9104749      0.335000      0.335014     -0.000014   298.95
  3.1750635      0.189600      0.189612     -0.000012   312.54
  3.4396521      0.105400      0.105358      0.000042   320.99
  3.7042407      0.058200      0.058178      0.000022   325.92
  3.9688294      0.032200      0.032233     -0.000033   328.71
  4.2334180      0.018000      0.018057     -0.000057   330.25
  4.7625952      0.006200      0.006151      0.000049   331.54
  5.2917725      0.002400      0.002749     -0.000349     0.10
-----------------------------------------------------------------
 SUM OF SQUARES OF ERRORS=0.1653D-05  AND THE STANDARD DEVIATION
IS=0.0009

     -------------------------------------------------------------
     |    OLD PARM    |    NEW PARM    | DELTA PARM |  ST. ERR.  |
     ------------------------------------------------------------
 r_e | 0.153975178D+01| 0.153975178D+01|-0.11102D-14| 0.42423D-04|
C[3] |-0.399415400D+01|-0.399415400D+01| 0.32419D-13| 0.10963D-02|
C[4] |-0.747423601D+00|-0.747423601D+00| 0.67391D-13| 0.36854D-02|
C[5] | 0.219608291D+01| 0.219608291D+01|-0.22871D-12| 0.81991D-02|
C[6] | 0.257070709D+01| 0.257070709D+01|-0.29976D-12| 0.21673D-01|
C[7] |-0.472134973D+00|-0.472134973D+00| 0.51603D-12| 0.21300D-01|
C[8] |-0.244351143D+01|-0.244351143D+01| 0.49161D-12| 0.48215D-01|
C[9] |-0.296234866D+00|-0.296234866D+00|-0.36648D-12| 0.17726D-01|
C[10]| 0.122652040D+01| 0.122652040D+01|-0.33729D-12| 0.39142D-01|
C[14]|-0.715049324D-01|-0.715049324D-01| 0.12584D-12| 0.65249D-02|
 V0  | 0.203326744D+01| 0.203326744D+01|-0.44409D-15| 0.36270D-04|
------------------------------------------------------------------
(a=0.7837373, a1=0.0, a2=0.2567  fixed after a preliminary determination)
=====================================================================
  y=1-exp{a*(r-r_e)+a1*(r-r_e)+a2*(r-r_e)^2
EQMF=V0+C[3]*y^2+C[4]*y^3+C[5]*y^4+C[6]*y^5+C[7]*y^6+C[8]*y^7+C[10]*y^9+C
[14]*y^13
```

Table S6. Smoothing the electric quadrupole moment function EQMF

of [Jozwiak H., Cybulski H., Wcislo, P.: J. Quant. Spectrosc. Radiat. Transfer 2020,253,107186.]

r/ANG	AB INITIO	CALCULATED	AI-C	WEIGHT
0.0000000	0.00000000	-0.01794312	0.01794312	0.00
0.2000000	0.04092350	0.03926378	0.00165972	0.01
0.2500000	0.06307330	0.06244962	0.00062368	0.01
0.3000000	0.08942946	0.08932738	0.00010208	1.00
0.3100000	0.09519012	0.09514154	0.00004858	1.00
0.3200000	0.10110670	0.10110012	0.00000659	1.00
0.3300000	0.10717684	0.10720209	-0.00002525	1.00
0.3400000	0.11339815	0.11344635	-0.00004820	1.00
0.3500000	0.11976820	0.11983166	-0.00006346	1.00
0.3600000	0.12628453	0.12635667	-0.00007213	1.00
0.3700000	0.13294465	0.13301991	-0.00007526	1.00
0.3800000	0.13974604	0.13981983	-0.00007380	1.00
0.3900000	0.14668615	0.14675476	-0.00006861	1.00
0.4000000	0.15376243	0.15382293	-0.00006050	1.00
0.4100000	0.16097228	0.16102246	-0.00005018	1.00
0.4200000	0.16831309	0.16835139	-0.00003829	1.00
0.4300000	0.17578223	0.17580765	-0.00002542	1.00
0.4400000	0.18337703	0.18338909	-0.00001206	1.00
0.4500000	0.19109482	0.19109348	0.00000134	1.00
0.4600000	0.19893290	0.19891849	0.00001441	1.00
0.4700000	0.20688852	0.20686170	0.00002682	1.00
0.4800000	0.21495894	0.21492065	0.00003829	1.00
0.4900000	0.22314137	0.22309276	0.00004860	1.00
0.5000000	0.23143300	0.23137541	0.00005759	1.00
0.5100000	0.23983101	0.23976588	0.00006513	1.00
0.5200000	0.24833253	0.24826143	0.00007111	1.00
0.5300000	0.25693470	0.25685920	0.00007550	1.00
0.5400000	0.26563296	0.26555632	0.00007664	1.00
0.5500000	0.27442922	0.27434983	0.00007939	1.00
0.5600000	0.28331573	0.28323674	0.00007899	1.00
0.5700000	0.29229107	0.29221399	0.00007708	1.00
0.5800000	0.30135223	0.30127847	0.00007375	1.00
0.5900000	0.31049616	0.31042705	0.00006911	1.00
0.6000000	0.31971981	0.31965653	0.00006328	1.00
0.6100000	0.32902006	0.32896368	0.00005638	1.00
0.6200000	0.33839378	0.33834523	0.00004856	1.00
0.6300000	0.34783782	0.34779787	0.00003996	1.00
0.6400000	0.35734899	0.35731827	0.00003073	1.00
0.6500000	0.36692407	0.36690305	0.00002102	1.00
0.6600000	0.37655981	0.37654881	0.00001100	1.00
0.6700000	0.38625294	0.38625213	0.00000081	1.00
0.6800000	0.39600014	0.39600954	-0.00000940	1.00
0.6900000	0.40579807	0.40581756	-0.00001949	1.00
0.7000000	0.41564338	0.41567269	-0.00002931	1.00
0.7100000	0.42553266	0.42557140	-0.00003874	1.00
0.7200000	0.43546248	0.43551013	-0.00004766	1.00
0.7300000	0.44542938	0.44548532	-0.00005594	1.00

0.7400000	0.45542987	0.45549338	-0.00006350	1.00
0.7500000	0.46546044	0.46553068	-0.00007025	1.00
0.7600000	0.47551752	0.47559361	-0.00007609	1.00
0.7700000	0.48559754	0.48567852	-0.00008097	1.00
0.7800000	0.49569688	0.49578173	-0.00008485	1.00
0.7900000	0.50581189	0.50589956	-0.00008766	1.00
0.8000000	0.51593890	0.51602831	-0.00008941	1.00
0.8100000	0.52607419	0.52616426	-0.00009006	1.00
0.8200000	0.53621404	0.53630366	-0.00008963	1.00
0.8300000	0.54635466	0.54644277	-0.00008812	1.00
0.8400000	0.55649225	0.55657781	-0.00008555	1.00
0.8500000	0.56662299	0.56670497	-0.00008198	1.00
0.8600000	0.57674301	0.57682045	-0.00007744	1.00
0.8700000	0.58684842	0.58692041	-0.00007199	1.00
0.8800000	0.59693529	0.59700100	-0.00006570	1.00
0.8900000	0.60699969	0.60705834	-0.00005865	1.00
0.9000000	0.61703762	0.61708854	-0.00005091	1.00
0.9100000	0.62704509	0.62708768	-0.00004259	1.00
0.9200000	0.63701806	0.63705182	-0.00003376	1.00
0.9300000	0.64695246	0.64697700	-0.00002454	1.00
0.9400000	0.65684422	0.65685924	-0.00001502	1.00
0.9500000	0.66668923	0.66669454	-0.00000530	1.00
0.9600000	0.67648335	0.67647885	0.00000450	1.00
0.9700000	0.68622243	0.68620814	0.00001429	1.00
0.9800000	0.69590229	0.69587833	0.00002396	1.00
0.9900000	0.70551873	0.70548532	0.00003341	1.00
1.0000000	0.71506755	0.71502500	0.00004255	1.00
1.0100000	0.72454450	0.72449321	0.00005129	1.00
1.0200000	0.73394534	0.73388581	0.00005953	1.00
1.0300000	0.74326581	0.74319860	0.00006721	1.00
1.0400000	0.75250162	0.75242739	0.00007423	1.00
1.0500000	0.76164849	0.76156795	0.00008055	1.00
1.0600000	0.77070213	0.77061604	0.00008609	1.00
1.0700000	0.77965823	0.77956741	0.00009082	1.00
1.0800000	0.78851248	0.78841780	0.00009468	1.00
1.0900000	0.79726056	0.79716291	0.00009765	1.00
1.1000000	0.80589816	0.80579846	0.00009970	1.00
1.1100000	0.81442097	0.81432015	0.00010082	1.00
1.1200000	0.82282467	0.82272366	0.00010101	1.00
1.1300000	0.83110497	0.83100470	0.00010026	1.00
1.1400000	0.83925757	0.83915896	0.00009861	1.00
1.1500000	0.84727819	0.84718212	0.00009607	1.00
1.1600000	0.85516256	0.85506989	0.00009267	1.00
1.1700000	0.86290645	0.86281799	0.00008846	1.00
1.1800000	0.87050562	0.87042213	0.00008349	1.00
1.1900000	0.87795587	0.87787806	0.00007781	1.00
1.2000000	0.88525305	0.88518154	0.00007150	1.00
1.2100000	0.89239301	0.89232837	0.00006463	1.00
1.2200000	0.89937165	0.89931438	0.00005728	1.00
1.2300000	0.90618493	0.90613541	0.00004952	1.00
1.2400000	0.91282882	0.91278737	0.00004145	1.00
1.2500000	0.91929937	0.91926620	0.00003316	1.00

1.2600000	0.92559266	0.92556791	0.00002475	1.00
1.2700000	0.93170485	0.93168854	0.00001631	1.00
1.2800000	0.93763215	0.93762422	0.00000793	1.00
1.2900000	0.94337085	0.94337112	-0.00000028	1.00
1.3000000	0.94891728	0.94892552	-0.00000823	1.00
1.3100000	0.95426790	0.95428373	-0.00001583	1.00
1.3200000	0.95941920	0.95944219	-0.00002299	1.00
1.3300000	0.96436779	0.96439741	-0.00002962	1.00
1.3400000	0.96911036	0.96914600	-0.00003564	1.00
1.3500000	0.97364370	0.97368468	-0.00004098	1.00
1.3600000	0.97796470	0.97801027	-0.00004557	1.00
1.3700000	0.98207036	0.98211970	-0.00004935	1.00
1.3800000	0.98595780	0.98601005	-0.00005225	1.00
1.3900000	0.98962425	0.98967849	-0.00005424	1.00
1.4000000	0.99306706	0.99312234	-0.00005528	1.00
1.4100000	0.99628372	0.99633907	-0.00005535	1.00
1.4200000	0.99927185	0.99932626	-0.00005441	1.00
1.4300000	1.00202921	1.00208168	-0.00005248	1.00
1.4400000	1.00455369	1.00460323	-0.00004954	1.00
1.4500000	1.00684335	1.00688896	-0.00004561	1.00
1.4600000	1.00889640	1.00893711	-0.00004071	1.00
1.4700000	1.01071120	1.01074608	-0.00003488	1.00
1.4800000	1.01228627	1.01231443	-0.00002815	1.00
1.4900000	1.01362032	1.01364090	-0.00002058	1.00
1.5000000	1.01471221	1.01472444	-0.00001223	1.00
1.5100000	1.01556099	1.01556415	-0.00000315	1000.00
1.5200000	1.01616588	1.01615932	0.00000656	1.00
1.5300000	1.01652629	1.01650946	0.00001683	1.00
1.5400000	1.01664182	1.01661425	0.00002757	1.00
1.5500000	1.01651226	1.01647357	0.00003869	1.00
1.5600000	1.01613758	1.01608750	0.00005008	1.00
1.5700000	1.01551796	1.01545632	0.00006164	1.00
1.5800000	1.01465378	1.01458051	0.00007327	1.00
1.5900000	1.01354561	1.01346076	0.00008485	1.00
1.6000000	1.01219424	1.01209794	0.00009629	1.00
1.6100000	1.01060063	1.01049316	0.00010747	1.00
1.6200000	1.00876598	1.00864769	0.00011829	1.00
1.6300000	1.00669167	1.00656303	0.00012864	1.00
1.6400000	1.00437930	1.00424088	0.00013842	1.00
1.6500000	1.00183066	1.00168312	0.00014753	1.00
1.6600000	0.99904775	0.99889186	0.00015589	1.00
1.6700000	0.99603278	0.99586937	0.00016341	1.00
1.6800000	0.99278814	0.99261814	0.00017000	1.00
1.6900000	0.98931645	0.98914084	0.00017560	1.00
1.7000000	0.98562048	0.98544033	0.00018015	1.00
1.7100000	0.98170323	0.98151964	0.00018360	1.00
1.7200000	0.97756789	0.97738200	0.00018589	1.00
1.7300000	0.97321780	0.97303081	0.00018699	1.00
1.7400000	0.96865651	0.96846962	0.00018689	1.00
1.7500000	0.96388775	0.96370219	0.00018556	1.00
1.7600000	0.95891539	0.95873239	0.00018300	1.00
1.7700000	0.95374351	0.95356428	0.00017923	1.00

1.7800000	0.94837630	0.94820205	0.00017425	1.00
1.7900000	0.94281814	0.94265004	0.00016810	1.00
1.8000000	0.93707355	0.93691274	0.00016081	1.00
1.8100000	0.93114717	0.93099474	0.00015244	1.00
1.8200000	0.92504380	0.92490077	0.00014303	1.00
1.8300000	0.91876835	0.91863569	0.00013266	1.00
1.8400000	0.91232584	0.91220444	0.00012140	1.00
1.8500000	0.90572141	0.90561208	0.00010933	1.00
1.8600000	0.89896031	0.89886377	0.00009654	1.00
1.8700000	0.89204786	0.89196473	0.00008312	1.00
1.8800000	0.88498947	0.88492030	0.00006917	1.00
1.8900000	0.87779064	0.87773584	0.00005480	1.00
1.9000000	0.87045692	0.87041681	0.00004011	1.00
1.9100000	0.86299393	0.86296873	0.00002520	1.00
1.9200000	0.85540733	0.85539713	0.00001020	1.00
1.9300000	0.84770282	0.84770761	-0.00000479	1.00
1.9400000	0.83988613	0.83990580	-0.00001967	1.00
1.9500000	0.83196302	0.83199734	-0.00003431	1.00
1.9600000	0.82393926	0.82398789	-0.00004863	1.00
1.9700000	0.81582063	0.81588313	-0.00006250	1.00
1.9800000	0.80761288	0.80768872	-0.00007584	1.00
1.9900000	0.79932178	0.79941034	-0.00008855	1.00
2.0000000	0.79095307	0.79105362	-0.00010055	1.00
2.0100000	0.78251246	0.78262419	-0.00011174	1.00
2.0200000	0.77400561	0.77412766	-0.00012205	1.00
2.0300000	0.76543817	0.76556959	-0.00013142	1.00
2.0400000	0.75681571	0.75695549	-0.00013978	1.00
2.0500000	0.74814375	0.74829083	-0.00014709	1.00
2.0600000	0.73942775	0.73958103	-0.00015328	1.00
2.0700000	0.73067310	0.73083144	-0.00015834	1.00
2.0800000	0.72188510	0.72204734	-0.00016224	1.00
2.0900000	0.71306900	0.71323394	-0.00016495	1.00
2.1000000	0.70422992	0.70439638	-0.00016646	1.00
2.1100000	0.69537291	0.69553969	-0.00016678	1.00
2.1200000	0.68650292	0.68666884	-0.00016592	1.00
2.1300000	0.67762480	0.67778869	-0.00016388	1.00
2.1400000	0.66874329	0.66890400	-0.00016071	1.00
2.1500000	0.65986302	0.66001944	-0.00015642	1.00
2.1600000	0.65098850	0.65113957	-0.00015107	1.00
2.1700000	0.64212413	0.64226882	-0.00014470	1.00
2.1800000	0.63327419	0.63341154	-0.00013736	1.00
2.1900000	0.62444283	0.62457195	-0.00012912	1.00
2.2000000	0.61563410	0.61575414	-0.00012004	1.00
2.2100000	0.60685190	0.60696209	-0.00011019	1.00
2.2200000	0.59810001	0.59819967	-0.00009966	1.00
2.2300000	0.58938208	0.58947060	-0.00008852	1.00
2.2400000	0.58070163	0.58077848	-0.00007686	1.00
2.2500000	0.57206205	0.57212681	-0.00006475	1.00
2.2600000	0.56346662	0.56351892	-0.00005230	1.00
2.2700000	0.55491846	0.55495804	-0.00003958	1.00
2.2800000	0.54642057	0.54644727	-0.00002670	1.00
2.2900000	0.53797583	0.53798955	-0.00001372	1.00

2.3000000	0.52958698	0.52958774	-0.00000076	1.00
2.3100000	0.52125664	0.52124453	0.00001212	1.00
2.3200000	0.51298730	0.51296249	0.00002482	1.00
2.3300000	0.50478133	0.50474407	0.00003725	1.00
2.3400000	0.49664096	0.49659160	0.00004936	1.00
2.3500000	0.48856832	0.48850727	0.00006104	1.00
2.3600000	0.48056539	0.48049315	0.00007224	1.00
2.3700000	0.47263408	0.47255119	0.00008289	1.00
2.3800000	0.46477613	0.46468321	0.00009292	1.00
2.3900000	0.45699320	0.45689091	0.00010228	1.00
2.4000000	0.44928682	0.44917590	0.00011092	1.00
2.4100000	0.44165843	0.44153965	0.00011878	1.00
2.4200000	0.43410934	0.43398351	0.00012583	1.00
2.4300000	0.42664078	0.42650874	0.00013204	1.00
2.4400000	0.41925386	0.41911649	0.00013737	1.00
2.4500000	0.41194960	0.41180779	0.00014181	1.00
2.4600000	0.40472891	0.40458357	0.00014534	1.00
2.4700000	0.39759263	0.39744469	0.00014794	1.00
2.4800000	0.39054148	0.39039187	0.00014962	1.00
2.4900000	0.38357613	0.38342576	0.00015037	1.00
2.5000000	0.37669713	0.37654693	0.00015021	1.00
2.5100000	0.36990497	0.36975583	0.00014914	1.00
2.5200000	0.36320004	0.36305285	0.00014719	1.00
2.5300000	0.35658267	0.35643829	0.00014438	1.00
2.5400000	0.35005312	0.34991238	0.00014074	1.00
2.5500000	0.34361156	0.34347526	0.00013630	1.00
2.5600000	0.33725810	0.33712700	0.00013110	1.00
2.5700000	0.33099280	0.33086762	0.00012518	1.00
2.5800000	0.32481564	0.32469705	0.00011859	1.00
2.5900000	0.31872653	0.31861516	0.00011137	1.00
2.6000000	0.31272536	0.31262178	0.00010358	1.00
2.6100000	0.30681192	0.30671665	0.00009526	1.00
2.6200000	0.30098597	0.30089949	0.00008648	1.00
2.6300000	0.29524724	0.29516994	0.00007730	1.00
2.6400000	0.28959537	0.28952761	0.00006776	1.00
2.6500000	0.28402998	0.28397205	0.00005793	1.00
2.6600000	0.27855066	0.27850279	0.00004787	1.00
2.6700000	0.27315692	0.27311928	0.00003764	1.00
2.6800000	0.26784827	0.26782097	0.00002730	1.00
2.6900000	0.26262416	0.26260725	0.00001691	1.00
2.7000000	0.25748402	0.25747750	0.00000652	1.00
2.7100000	0.25242724	0.25243104	-0.00000380	1.00
2.7200000	0.24745318	0.24746718	-0.00001400	1.00
2.7300000	0.24256118	0.24258521	-0.00002403	1.00
2.7400000	0.23775054	0.23778438	-0.00003384	1.00
2.7500000	0.23302055	0.23306391	-0.00004336	1.00
2.7600000	0.22837046	0.22842303	-0.00005257	1.00
2.7700000	0.22379952	0.22386094	-0.00006141	1.00
2.7800000	0.21930695	0.21937680	-0.00006985	1.00
2.7900000	0.21489194	0.21496978	-0.00007784	1.00
2.8000000	0.21055368	0.21063903	-0.00008535	1.00
2.8100000	0.20629134	0.20638369	-0.00009235	1.00

2.8200000	0.20210408	0.20220289	-0.00009880	1.00
2.8300000	0.19799104	0.19809574	-0.00010470	1.00
2.8400000	0.19395136	0.19406136	-0.00011000	1.00
2.8500000	0.18998415	0.19009885	-0.00011470	1.00
2.8600000	0.18608853	0.18620731	-0.00011878	1.00
2.8700000	0.18226361	0.18238584	-0.00012223	1.00
2.8800000	0.17850849	0.17863353	-0.00012505	1.00
2.8900000	0.17482226	0.17494948	-0.00012722	1.00
2.9000000	0.17120401	0.17133276	-0.00012875	1.00
2.9100000	0.16765284	0.16778248	-0.00012964	1.00
2.9200000	0.16416782	0.16429771	-0.00012990	1.00
2.9300000	0.16074804	0.16087756	-0.00012953	1.00
2.9400000	0.15739257	0.15752112	-0.00012854	1.00
2.9500000	0.15410052	0.15422748	-0.00012696	1.00
2.9600000	0.15087094	0.15099574	-0.00012480	1.00
2.9700000	0.14770293	0.14782500	-0.00012207	1.00
2.9800000	0.14459558	0.14471438	-0.00011880	1.00
2.9900000	0.14154797	0.14166298	-0.00011501	1.00
3.0000000	0.13855919	0.13866992	-0.00011074	1.00
3.0100000	0.13562834	0.13573433	-0.00010599	1.00
3.0200000	0.13275451	0.13285533	-0.00010082	1.00
3.0300000	0.12993681	0.13003205	-0.00009524	1.00
3.0400000	0.12717435	0.12726364	-0.00008928	1.00
3.0500000	0.12446625	0.12454924	-0.00008299	1.00
3.0600000	0.12181162	0.12188801	-0.00007639	1.00
3.0700000	0.11920958	0.11927911	-0.00006953	1.00
3.0800000	0.11665928	0.11672171	-0.00006242	1.00
3.0900000	0.11415986	0.11421497	-0.00005512	1.00
3.1000000	0.11171045	0.11175810	-0.00004765	1.00
3.1100000	0.10931022	0.10935027	-0.00004005	1.00
3.1200000	0.10695832	0.10699068	-0.00003236	1.00
3.1300000	0.10465394	0.10467855	-0.00002461	1.00
3.1400000	0.10239624	0.10241307	-0.00001683	1.00
3.1500000	0.10018442	0.10019348	-0.00000907	1.00
3.1600000	0.09801767	0.09801901	-0.00000134	1.00
3.1700000	0.09589519	0.09588889	0.00000630	1.00
3.1800000	0.09381621	0.09380236	0.00001385	1.00
3.1900000	0.09177994	0.09175869	0.00002125	1.00
3.2000000	0.08978562	0.08975712	0.00002849	1.00
3.2100000	0.08783248	0.08779694	0.00003554	1.00
3.2200000	0.08591978	0.08587741	0.00004237	1.00
3.2300000	0.08404678	0.08399783	0.00004896	1.00
3.2400000	0.08221275	0.08215748	0.00005527	1.00
3.2500000	0.08041697	0.08035567	0.00006130	1.00
3.2600000	0.07865873	0.07859171	0.00006702	1.00
3.2700000	0.07693733	0.07686491	0.00007242	1.00
3.2800000	0.07525206	0.07517460	0.00007747	1.00
3.2900000	0.07360227	0.07352011	0.00008216	1.00
3.3000000	0.07198726	0.07190079	0.00008648	1.00
3.3100000	0.07040639	0.07031597	0.00009041	1.00
3.3200000	0.06885899	0.06876503	0.00009396	1.00
3.3300000	0.06734443	0.06724733	0.00009710	1.00

3.3400000	0.06586207	0.06576223	0.00009984	1.00
3.3500000	0.06441128	0.06430911	0.00010217	1.00
3.3600000	0.06299146	0.06288738	0.00010408	1.00
3.3700000	0.06160200	0.06149642	0.00010558	1.00
3.3800000	0.06024231	0.06013564	0.00010667	1.00
3.3900000	0.05891179	0.05880445	0.00010734	1.00
3.4000000	0.05760988	0.05750227	0.00010761	1.00
3.4100000	0.05633600	0.05622852	0.00010748	1.00
3.4200000	0.05508961	0.05498266	0.00010695	1.00
3.4300000	0.05387014	0.05376411	0.00010603	

1.00SM_H2_draft_a.odt

3.4400000	0.05267707	0.05257233	0.00010474	1.00
3.4500000	0.05150986	0.05140679	0.00010307	1.00
3.4600000	0.05036799	0.05026693	0.00010105	1.00
3.4700000	0.04925095	0.04915226	0.00009869	1.00
3.4800000	0.04815823	0.04806223	0.00009600	1.00
3.4900000	0.04708935	0.04699635	0.00009299	1.00
3.5000000	0.04604381	0.04595412	0.00008969	1.00
3.5100000	0.04502114	0.04493504	0.00008610	1.00
3.5200000	0.04402086	0.04393862	0.00008225	1.00
3.5300000	0.04304252	0.04296438	0.00007814	1.00
3.5400000	0.04208567	0.04201186	0.00007381	1.00
3.5500000	0.04114986	0.04108059	0.00006927	1.00
3.5600000	0.04023465	0.04017012	0.00006454	1.00
3.5700000	0.03933962	0.03927999	0.00005963	1.00
3.5800000	0.03846435	0.03840977	0.00005457	1.00
3.5900000	0.03760841	0.03755903	0.00004938	1.00
3.6000000	0.03677142	0.03672734	0.00004408	1.00
3.6100000	0.03595296	0.03591428	0.00003868	1.00
3.6200000	0.03515266	0.03511944	0.00003321	1.00
3.6300000	0.03437011	0.03434242	0.00002769	1.00
3.6400000	0.03360497	0.03358282	0.00002214	1.00
3.6500000	0.03285684	0.03284026	0.00001658	1.00
3.6600000	0.03212537	0.03211436	0.00001102	1.00
3.6700000	0.03141021	0.03140473	0.00000548	1.00
3.6800000	0.03071101	0.03071102	-0.00000001	1.00
3.6900000	0.03002743	0.03003286	-0.00000543	1.00
3.7000000	0.02935913	0.02936990	-0.00001078	1.00
3.7100000	0.02870578	0.02872180	-0.00001602	1.00
3.7200000	0.02806707	0.02808822	-0.00002115	1.00
3.7300000	0.02744268	0.02746883	-0.00002615	1.00
3.7400000	0.02683229	0.02686330	-0.00003101	1.00
3.7500000	0.02623562	0.02627131	-0.00003569	1.00
3.7600000	0.02565235	0.02569255	-0.00004021	1.00
3.7700000	0.02508220	0.02512672	-0.00004453	1.00
3.7800000	0.02452488	0.02457353	-0.00004864	1.00
3.7900000	0.02398012	0.02403266	-0.00005254	1.00
3.8000000	0.02344764	0.02350385	-0.00005621	1.00
3.8100000	0.02292717	0.02298681	-0.00005964	1.00
3.8200000	0.02241845	0.02248127	-0.00006282	1.00
3.8300000	0.02192122	0.02198696	-0.00006574	1.00
3.8400000	0.02143524	0.02150363	-0.00006839	1.00

3.8500000	0.02096024	0.02103100	-0.00007076	1.00
3.8600000	0.02049599	0.02056885	-0.00007285	1.00
3.8700000	0.02004226	0.02011691	-0.00007465	1.00
3.8800000	0.01959881	0.01967496	-0.00007616	1.00
3.8900000	0.01916541	0.01924277	-0.00007736	1.00
3.9000000	0.01874184	0.01882009	-0.00007825	1.00
3.9100000	0.01832789	0.01840672	-0.00007884	1.00
3.9200000	0.01792333	0.01800244	-0.00007911	1.00
3.9300000	0.01752797	0.01760703	-0.00007906	1.00
3.9400000	0.01714159	0.01722029	-0.00007871	1.00
3.9500000	0.01676400	0.01684202	-0.00007803	1.00
3.9600000	0.01639499	0.01647203	-0.00007703	1.00
3.9700000	0.01603438	0.01611011	-0.00007572	1.00
3.9800000	0.01568199	0.01575608	-0.00007409	1.00
3.9900000	0.01533761	0.01540977	-0.00007215	1.00
4.0000000	0.01500108	0.01507098	-0.00006990	1.00
4.0100000	0.01467223	0.01473956	-0.00006733	1.00
4.0200000	0.01435086	0.01441533	-0.00006446	1.00
4.0300000	0.01403683	0.01409812	-0.00006129	1.00
4.0400000	0.01372996	0.01378778	-0.00005782	1.00
4.0500000	0.01343008	0.01348414	-0.00005406	1.00
4.0600000	0.01313706	0.01318706	-0.00005001	1.00
4.0700000	0.01285071	0.01289639	-0.00004567	1.00
4.0800000	0.01257091	0.01261198	-0.00004107	1.00
4.0900000	0.01229749	0.01233368	-0.00003619	1.00
4.1000000	0.01203032	0.01206137	-0.00003105	1.00
4.1100000	0.01176925	0.01179490	-0.00002565	0.01
4.1200000	0.01151414	0.01153415	-0.00002000	0.01
4.1300000	0.01126486	0.01127898	-0.00001412	0.01
4.1400000	0.01102127	0.01102928	-0.00000800	0.01
4.1500000	0.01078325	0.01078491	-0.00000166	0.01
4.1600000	0.01055067	0.01054577	0.00000489	0.01
4.1700000	0.01032340	0.01031174	0.00001166	0.01
4.1800000	0.01010132	0.01008270	0.00001862	0.01
4.1900000	0.00988431	0.00985854	0.00002577	0.01
4.2000000	0.00967226	0.00963917	0.00003310	0.01
4.2100000	0.00946506	0.00942446	0.00004060	0.01
4.2200000	0.00926258	0.00921433	0.00004826	0.01
4.2300000	0.00906474	0.00900866	0.00005607	0.01
4.2400000	0.00887140	0.00880738	0.00006403	0.01
4.2500000	0.00868248	0.00861037	0.00007211	0.01
4.2600000	0.00849788	0.00841755	0.00008032	0.01
4.2700000	0.00831748	0.00822883	0.00008865	0.01
4.2800000	0.00814120	0.00804413	0.00009708	0.01
4.2900000	0.00796895	0.00786334	0.00010560	0.01
4.3000000	0.00780061	0.00768640	0.00011421	0.01
4.3100000	0.00763612	0.00751323	0.00012290	0.01
4.3200000	0.00747538	0.00734373	0.00013165	0.01
4.3300000	0.00731829	0.00717784	0.00014046	0.01
4.3400000	0.00716479	0.00701547	0.00014931	0.01
4.3500000	0.00701477	0.00685656	0.00015821	0.01
4.3600000	0.00686818	0.00670104	0.00016714	0.01

4.3700000	0.00672491	0.00654882	0.00017609	0.01
4.3800000	0.00658490	0.00639985	0.00018505	0.01
4.3900000	0.00644808	0.00625406	0.00019402	0.01
4.4000000	0.00631436	0.00611137	0.00020299	0.01
4.4100000	0.00618367	0.00597173	0.00021194	0.01
4.4200000	0.00605595	0.00583508	0.00022087	0.01
4.4300000	0.00593113	0.00570135	0.00022978	0.01
4.4400000	0.00580914	0.00557048	0.00023866	0.01
4.4500000	0.00568990	0.00544242	0.00024749	0.01
4.4600000	0.00557337	0.00531710	0.00025627	0.01
4.4700000	0.00545947	0.00519448	0.00026500	0.01
4.4800000	0.00534815	0.00507449	0.00027366	0.01
4.4900000	0.00523934	0.00495708	0.00028226	0.01
4.5000000	0.00513299	0.00484221	0.00029078	0.01
4.5100000	0.00502903	0.00472981	0.00029922	0.01
4.5200000	0.00492742	0.00461985	0.00030758	0.01
4.5300000	0.00482810	0.00451226	0.00031584	0.01
4.5400000	0.00473101	0.00440701	0.00032400	0.01
4.5500000	0.00463611	0.00430405	0.00033206	0.01
4.5600000	0.00454334	0.00420332	0.00034002	0.01
4.5700000	0.00445265	0.00410479	0.00034786	0.01
4.5800000	0.00436399	0.00400841	0.00035558	0.01
4.5900000	0.00427732	0.00391414	0.00036318	0.01
4.6000000	0.00419259	0.00382193	0.00037066	0.01
4.6100000	0.00410976	0.00373174	0.00037801	0.01
4.6200000	0.00402877	0.00364354	0.00038523	0.01
4.6300000	0.00394960	0.00355729	0.00039231	0.01
4.6400000	0.00387219	0.00347293	0.00039925	0.01
4.6500000	0.00379650	0.00339045	0.00040606	0.01
4.6600000	0.00372250	0.00330979	0.00041271	0.01
4.6700000	0.00365015	0.00323093	0.00041922	0.01
4.6800000	0.00357940	0.00315382	0.00042558	0.01
4.6900000	0.00351023	0.00307843	0.00043179	0.01
4.7000000	0.00344258	0.00300473	0.00043785	0.01
4.7100000	0.00337644	0.00293268	0.00044375	0.01
4.7200000	0.00331175	0.00286225	0.00044950	0.01
4.7300000	0.00324850	0.00279341	0.00045509	0.01
4.7400000	0.00318664	0.00272612	0.00046052	0.01
4.7500000	0.00312615	0.00266036	0.00046579	0.01
4.7600000	0.00306699	0.00259609	0.00047090	0.01
4.7700000	0.00300913	0.00253328	0.00047585	0.01
4.7800000	0.00295254	0.00247190	0.00048064	0.01
4.7900000	0.00289719	0.00241193	0.00048526	0.01
4.8000000	0.00284306	0.00235333	0.00048973	0.01
4.8100000	0.00279011	0.00229608	0.00049403	0.01
4.8200000	0.00273832	0.00224015	0.00049817	0.01
4.8300000	0.00268766	0.00218552	0.00050214	0.01
4.8400000	0.00263810	0.00213214	0.00050596	0.01
4.8500000	0.00258963	0.00208001	0.00050962	0.01
4.8600000	0.00254221	0.00202910	0.00051311	0.01
4.8700000	0.00249582	0.00197937	0.00051645	0.01
4.8800000	0.00245043	0.00193081	0.00051963	0.01

4.8900000	0.00240603	0.00188339	0.00052265	0.01
4.9000000	0.00236259	0.00183708	0.00052551	0.01
4.9100000	0.00232009	0.00179187	0.00052822	0.01
4.9200000	0.00227851	0.00174774	0.00053077	0.01
4.9300000	0.00223782	0.00170465	0.00053317	0.01
4.9400000	0.00219801	0.00166259	0.00053542	0.01
4.9500000	0.00215905	0.00162153	0.00053752	0.01
4.9600000	0.00212093	0.00158146	0.00053947	0.01
4.9700000	0.00208363	0.00154235	0.00054128	0.01
4.9800000	0.00204713	0.00150419	0.00054294	0.01
4.9900000	0.00201140	0.00146694	0.00054446	0.01
5.0000000	0.00197644	0.00143061	0.00054583	0.01
5.0100000	0.00194222	0.00139515	0.00054707	0.01
5.0200000	0.00190873	0.00136056	0.00054817	0.01
5.0300000	0.00187595	0.00132682	0.00054913	0.01
5.0400000	0.00184387	0.00129391	0.00054996	0.01
5.0500000	0.00181246	0.00126181	0.00055065	0.01
5.0600000	0.00178172	0.00123050	0.00055122	0.01
5.0700000	0.00175163	0.00119996	0.00055166	0.01
5.0800000	0.00172217	0.00117019	0.00055198	0.01
5.0900000	0.00169333	0.00114116	0.00055217	0.01
5.1000000	0.00166509	0.00111285	0.00055224	0.01
5.1100000	0.00163745	0.00108526	0.00055219	0.01
5.1200000	0.00161038	0.00105835	0.00055203	0.01
5.1300000	0.00158388	0.00103213	0.00055175	0.01
5.1400000	0.00155793	0.00100657	0.00055135	0.01
5.1500000	0.00153251	0.00098166	0.00055085	0.01
5.1600000	0.00150763	0.00095739	0.00055024	0.01
5.1700000	0.00148326	0.00093373	0.00054952	0.01
5.1800000	0.00145939	0.00091069	0.00054871	0.01
5.1900000	0.00143602	0.00088823	0.00054778	0.01
5.2000000	0.00141312	0.00086635	0.00054677	0.01
5.2100000	0.00139069	0.00084504	0.00054565	0.01
5.2200000	0.00136872	0.00082429	0.00054444	0.01
5.2300000	0.00134720	0.00080407	0.00054313	0.01
5.2400000	0.00132612	0.00078438	0.00054174	0.01
5.2500000	0.00130547	0.00076521	0.00054026	0.01
5.2600000	0.00128523	0.00074654	0.00053869	0.01
5.2700000	0.00126540	0.00072837	0.00053704	0.01
5.2800000	0.00124597	0.00071067	0.00053530	0.01
5.2900000	0.00122693	0.00069344	0.00053349	0.01
5.3000000	0.00120827	0.00067668	0.00053160	0.01
5.3100000	0.00118998	0.00066036	0.00052963	0.01
5.3200000	0.00117206	0.00064447	0.00052759	0.01
5.3300000	0.00115449	0.00062902	0.00052547	0.01
5.3400000	0.00113727	0.00061398	0.00052329	0.01
5.3500000	0.00112039	0.00059935	0.00052104	0.01
5.3600000	0.00110383	0.00058511	0.00051872	0.01
5.3700000	0.00108761	0.00057126	0.00051634	0.01
5.3800000	0.00107169	0.00055779	0.00051390	0.01
5.3900000	0.00105609	0.00054469	0.00051140	0.01
5.4000000	0.00104079	0.00053195	0.00050884	0.01

5.4100000	0.00102578	0.00051956	0.00050622	0.01
5.4200000	0.00101106	0.00050751	0.00050355	0.01
5.4300000	0.00099662	0.00049579	0.00050082	0.01
5.4400000	0.00098245	0.00048440	0.00049805	0.01
5.4500000	0.00096855	0.00047333	0.00049522	0.01
5.4600000	0.00095492	0.00046257	0.00049234	0.01
5.4700000	0.00094153	0.00045211	0.00048942	0.01
5.4800000	0.00092840	0.00044195	0.00048646	0.01
5.4900000	0.00091552	0.00043207	0.00048345	0.01
5.5000000	0.00090287	0.00042247	0.00048040	0.01
5.5100000	0.00089045	0.00041315	0.00047730	0.01
5.5200000	0.00087826	0.00040409	0.00047417	0.01
5.5300000	0.00086630	0.00039529	0.00047101	0.01
5.5400000	0.00085454	0.00038674	0.00046780	0.01
5.5500000	0.00084301	0.00037844	0.00046457	0.01
5.5600000	0.00083167	0.00037038	0.00046130	0.01
5.5700000	0.00082054	0.00036255	0.00045799	0.01
5.5800000	0.00080961	0.00035495	0.00045466	0.01
5.5900000	0.00079887	0.00034757	0.00045130	0.01
5.6000000	0.00078832	0.00034040	0.00044791	0.01
5.6100000	0.00077795	0.00033345	0.00044450	0.01
5.6200000	0.00076776	0.00032670	0.00044106	0.01
5.6300000	0.00075775	0.00032015	0.00043760	0.01
5.6400000	0.00074790	0.00031379	0.00043411	0.01
5.6500000	0.00073823	0.00030763	0.00043060	0.01
5.6600000	0.00072872	0.00030164	0.00042708	0.01
5.6700000	0.00071937	0.00029584	0.00042353	0.01
5.6800000	0.00071017	0.00029020	0.00041997	0.01
5.6900000	0.00070113	0.00028474	0.00041639	0.01
5.7000000	0.00069223	0.00027944	0.00041279	0.01
5.7100000	0.00068349	0.00027430	0.00040918	0.01
5.7200000	0.00067488	0.00026932	0.00040556	0.01
5.7300000	0.00066641	0.00026449	0.00040192	0.01
5.7400000	0.00065808	0.00025981	0.00039827	0.01
5.7500000	0.00064988	0.00025527	0.00039462	0.01
5.7600000	0.00064182	0.00025087	0.00039095	0.01
5.7700000	0.00063387	0.00024660	0.00038727	0.01
5.7800000	0.00062606	0.00024247	0.00038359	0.01
5.7900000	0.00061836	0.00023846	0.00037990	0.01
5.8000000	0.00061079	0.00023458	0.00037621	0.01
5.8100000	0.00060333	0.00023082	0.00037251	0.01
5.8200000	0.00059598	0.00022718	0.00036880	0.01
5.8300000	0.00058875	0.00022365	0.00036510	0.01
5.8400000	0.00058162	0.00022023	0.00036139	0.01
5.8500000	0.00057461	0.00021692	0.00035768	0.01
5.8600000	0.00056769	0.00021372	0.00035398	0.01
5.8700000	0.00056088	0.00021061	0.00035027	0.01
5.8800000	0.00055417	0.00020761	0.00034656	0.01
5.8900000	0.00054756	0.00020470	0.00034286	0.01
5.9000000	0.00054104	0.00020188	0.00033916	0.01
5.9100000	0.00053462	0.00019916	0.00033546	0.01
5.9200000	0.00052829	0.00019652	0.00033177	0.01

5.9300000	0.00052205	0.00019396	0.00032809	0.01
5.9400000	0.00051590	0.00019149	0.00032441	0.01
5.9500000	0.00050984	0.00018910	0.00032073	0.01
5.9600000	0.00050386	0.00018679	0.00031707	0.01
5.9700000	0.00049797	0.00018456	0.00031341	0.01
5.9800000	0.00049216	0.00018239	0.00030977	0.01
5.9900000	0.00048643	0.00018030	0.00030613	0.01
6.0000000	0.00048078	0.00017828	0.00030250	0.01
6.0100000	0.00047521	0.00017632	0.00029889	0.01
6.0200000	0.00046971	0.00017443	0.00029528	0.01
6.0300000	0.00046429	0.00017260	0.00029169	0.01
6.0400000	0.00045894	0.00017083	0.00028811	0.01
6.0500000	0.00045367	0.00016912	0.00028455	0.01
6.0600000	0.00044847	0.00016747	0.00028100	0.01
6.0700000	0.00044334	0.00016587	0.00027746	0.01
6.0800000	0.00043828	0.00016433	0.00027394	0.01
6.0900000	0.00043328	0.00016284	0.00027044	0.01
6.1000000	0.00042835	0.00016140	0.00026695	0.01
6.1100000	0.00042349	0.00016001	0.00026348	0.01
6.1200000	0.00041870	0.00015867	0.00026003	0.01
6.1300000	0.00041397	0.00015737	0.00025659	0.01
6.1400000	0.00040930	0.00015612	0.00025318	0.01
6.1500000	0.00040469	0.00015491	0.00024978	0.01
6.1600000	0.00040015	0.00015374	0.00024640	0.01
6.1700000	0.00039566	0.00015261	0.00024305	0.01
6.1800000	0.00039124	0.00015153	0.00023971	0.01
6.1900000	0.00038687	0.00015047	0.00023639	0.01
6.2000000	0.00038256	0.00014946	0.00023310	0.01
6.2100000	0.00037831	0.00014848	0.00022983	0.01
6.2200000	0.00037412	0.00014754	0.00022658	0.01
6.2300000	0.00036998	0.00014663	0.00022335	0.01
6.2400000	0.00036589	0.00014575	0.00022014	0.01
6.2500000	0.00036186	0.00014490	0.00021696	0.01
6.2600000	0.00035789	0.00014408	0.00021380	0.01
6.2700000	0.00035396	0.00014330	0.00021067	0.01
6.2800000	0.00035009	0.00014253	0.00020756	0.01
6.2900000	0.00034627	0.00014180	0.00020447	0.01
6.3000000	0.00034251	0.00014109	0.00020141	0.01
6.3100000	0.00033879	0.00014041	0.00019837	0.01
6.3200000	0.00033512	0.00013976	0.00019536	0.01
6.3300000	0.00033150	0.00013912	0.00019238	0.01
6.3400000	0.00032793	0.00013851	0.00018942	0.01
6.3500000	0.00032441	0.00013793	0.00018649	0.01
6.3600000	0.00032094	0.00013736	0.00018358	0.01
6.3700000	0.00031751	0.00013681	0.00018070	0.01
6.3800000	0.00031413	0.00013629	0.00017784	0.01
6.3900000	0.00031079	0.00013578	0.00017501	0.01
6.4000000	0.00030751	0.00013529	0.00017221	0.01
6.4100000	0.00030426	0.00013482	0.00016944	0.01
6.4200000	0.00030106	0.00013437	0.00016669	0.01
6.4300000	0.00029791	0.00013394	0.00016397	0.01
6.4400000	0.00029480	0.00013352	0.00016128	0.01

6.4500000	0.00029173	0.00013311	0.00015862	0.01
6.4600000	0.00028870	0.00013272	0.00015598	0.01
6.4700000	0.00028572	0.00013235	0.00015337	0.01
6.4800000	0.00028278	0.00013199	0.00015079	0.01
6.4900000	0.00027988	0.00013164	0.00014823	0.01
6.5000000	0.00027701	0.00013131	0.00014570	0.01
6.5100000	0.00027420	0.00013099	0.00014320	0.01
6.5200000	0.00027142	0.00013068	0.00014073	0.01
6.5300000	0.00026867	0.00013039	0.00013829	0.01
6.5400000	0.00026597	0.00013010	0.00013587	0.01
6.5500000	0.00026331	0.00012983	0.00013348	0.01
6.5600000	0.00026068	0.00012956	0.00013112	0.01
6.5700000	0.00025810	0.00012931	0.00012879	0.01
6.5800000	0.00025555	0.00012907	0.00012648	0.01
6.5900000	0.00025303	0.00012883	0.00012420	0.01
6.6000000	0.00025056	0.00012861	0.00012195	0.01
6.6100000	0.00024812	0.00012839	0.00011972	0.01
6.6200000	0.00024571	0.00012818	0.00011753	0.01
6.6300000	0.00024334	0.00012798	0.00011536	0.01
6.6400000	0.00024100	0.00012779	0.00011321	0.01
6.6500000	0.00023870	0.00012761	0.00011109	0.01
6.6600000	0.00023643	0.00012743	0.00010900	0.01
6.6700000	0.00023420	0.00012726	0.00010694	0.01
6.6800000	0.00023200	0.00012710	0.00010490	0.01
6.6900000	0.00022983	0.00012694	0.00010289	0.01
6.7000000	0.00022769	0.00012679	0.00010090	0.01
6.7100000	0.00022558	0.00012665	0.00009894	0.01
6.7200000	0.00022351	0.00012651	0.00009700	0.01
6.7300000	0.00022147	0.00012637	0.00009509	0.01
6.7400000	0.00021945	0.00012625	0.00009321	0.01
6.7500000	0.00021747	0.00012612	0.00009135	0.01
6.7600000	0.00021552	0.00012601	0.00008951	0.01
6.7700000	0.00021359	0.00012589	0.00008770	0.01
6.7800000	0.00021170	0.00012579	0.00008591	0.01
6.7900000	0.00020983	0.00012568	0.00008415	0.01
6.8000000	0.00020799	0.00012558	0.00008241	0.01
6.8100000	0.00020618	0.00012549	0.00008070	0.01
6.8200000	0.00020440	0.00012540	0.00007900	0.01
6.8300000	0.00020264	0.00012531	0.00007733	0.01
6.8400000	0.00020091	0.00012522	0.00007568	0.01
6.8500000	0.00019920	0.00012514	0.00007406	0.01
6.8600000	0.00019752	0.00012507	0.00007246	0.01
6.8700000	0.00019587	0.00012499	0.00007088	0.01
6.8800000	0.00019424	0.00012492	0.00006932	0.01
6.8900000	0.00019263	0.00012485	0.00006778	0.01
6.9000000	0.00019105	0.00012479	0.00006626	0.01
6.9100000	0.00018949	0.00012473	0.00006477	0.01
6.9200000	0.00018796	0.00012467	0.00006329	0.01
6.9300000	0.00018644	0.00012461	0.00006183	0.01
6.9400000	0.00018495	0.00012455	0.00006040	0.01
6.9500000	0.00018348	0.00012450	0.00005898	0.01
6.9600000	0.00018204	0.00012445	0.00005759	0.01

6.9700000	0.00018061	0.00012440	0.00005621	0.01
6.9800000	0.00017921	0.00012436	0.00005485	0.01
6.9900000	0.00017782	0.00012431	0.00005351	0.01
7.0000000	0.00017646	0.00012427	0.00005219	0.01
7.0100000	0.00017511	0.00012423	0.00005088	0.01
7.0200000	0.00017379	0.00012419	0.00004959	0.01
7.0300000	0.00017248	0.00012416	0.00004832	0.01
7.0400000	0.00017119	0.00012412	0.00004707	0.01
7.0500000	0.00016992	0.00012409	0.00004583	0.01
7.0600000	0.00016867	0.00012405	0.00004461	0.01
7.0700000	0.00016743	0.00012402	0.00004341	0.01
7.0800000	0.00016622	0.00012399	0.00004222	0.01
7.0900000	0.00016501	0.00012397	0.00004105	0.01
7.1000000	0.00016383	0.00012394	0.00003989	0.01
7.1100000	0.00016266	0.00012391	0.00003875	0.01
7.1200000	0.00016151	0.00012389	0.00003762	0.01
7.1300000	0.00016037	0.00012386	0.00003651	0.01
7.1400000	0.00015925	0.00012384	0.00003541	0.01
7.1500000	0.00015814	0.00012382	0.00003432	0.01
7.1600000	0.00015705	0.00012380	0.00003325	0.01
7.1700000	0.00015597	0.00012378	0.00003219	0.01
7.1800000	0.00015490	0.00012376	0.00003114	0.01
7.1900000	0.00015385	0.00012374	0.00003011	0.01
7.2000000	0.00015281	0.00012373	0.00002908	0.01
7.2100000	0.00015178	0.00012371	0.00002807	0.01
7.2200000	0.00015077	0.00012370	0.00002708	0.01
7.2300000	0.00014977	0.00012368	0.00002609	0.01
7.2400000	0.00014878	0.00012367	0.00002511	0.01
7.2500000	0.00014780	0.00012365	0.00002415	0.01
7.2600000	0.00014684	0.00012364	0.00002320	0.01
7.2700000	0.00014588	0.00012363	0.00002225	0.01
7.2800000	0.00014494	0.00012362	0.00002132	0.01
7.2900000	0.00014400	0.00012360	0.00002040	0.01
7.3000000	0.00014308	0.00012359	0.00001948	0.01
7.3100000	0.00014216	0.00012358	0.00001858	0.01
7.3200000	0.00014126	0.00012357	0.00001769	0.01
7.3300000	0.00014037	0.00012357	0.00001680	0.01
7.3400000	0.00013948	0.00012356	0.00001592	0.01
7.3500000	0.00013860	0.00012355	0.00001506	0.01
7.3600000	0.00013774	0.00012354	0.00001420	0.01
7.3700000	0.00013688	0.00012353	0.00001335	0.01
7.3800000	0.00013603	0.00012353	0.00001250	0.01
7.3900000	0.00013519	0.00012352	0.00001167	0.01
7.4000000	0.00013435	0.00012351	0.00001084	0.01
7.4100000	0.00013353	0.00012351	0.00001002	0.01
7.4600000	0.00012950	0.00012348	0.00000602	0.01
7.4700000	0.00012871	0.00012347	0.00000524	0.01
7.4800000	0.00012794	0.00012347	0.00000447	0.01
7.4900000	0.00012716	0.00012346	0.00000370	0.01
7.5000000	0.00012640	0.00012346	0.00000294	0.01
7.5100000	0.00012564	0.00012346	0.00000218	0.01
7.5600000	0.00012191	0.00012344	-0.00000153	0.01

7.5700000	0.00012118	0.00012344	-0.00000225	0.01
7.5800000	0.00012046	0.00012343	-0.00000298	0.01
7.5900000	0.00011974	0.00012343	-0.00000369	0.01
7.6000000	0.00011902	0.00012343	-0.00000441	0.01
7.6100000	0.00011831	0.00012343	-0.00000512	0.01
7.6200000	0.00011760	0.00012343	-0.00000582	0.01
7.6300000	0.00011690	0.00012342	-0.00000652	0.01
7.6400000	0.00011620	0.00012342	-0.00000722	0.01
7.6500000	0.00011551	0.00012342	-0.00000791	0.01
7.6600000	0.00011481	0.00012342	-0.00000860	0.01
7.6700000	0.00011413	0.00012342	-0.00000929	0.01
7.6800000	0.00011344	0.00012341	-0.00000997	0.01
7.6900000	0.00011276	0.00012341	-0.00001065	0.01
7.7000000	0.00011208	0.00012341	-0.00001133	0.01
7.7100000	0.00011141	0.00012341	-0.00001200	0.01
7.7200000	0.00011073	0.00012341	-0.00001267	0.01
7.7300000	0.00011007	0.00012341	-0.00001334	0.01
7.7400000	0.00010940	0.00012341	-0.00001401	0.01
7.7500000	0.00010874	0.00012341	-0.00001467	0.01
7.7600000	0.00010808	0.00012340	-0.00001533	0.01
7.7700000	0.00010742	0.00012340	-0.00001598	0.01
7.7800000	0.00010676	0.00012340	-0.00001664	0.01
7.7900000	0.00010611	0.00012340	-0.00001729	0.01
7.8000000	0.00010546	0.00012340	-0.00001794	0.01
7.8100000	0.00010482	0.00012340	-0.00001858	0.01
7.8200000	0.00010417	0.00012340	-0.00001923	0.01
7.8300000	0.00010353	0.00012340	-0.00001987	0.01
7.8400000	0.00010289	0.00012340	-0.00002051	0.01
7.8500000	0.00010225	0.00012340	-0.00002115	0.01
7.8600000	0.00010161	0.00012340	-0.00002178	0.01
7.8700000	0.00010098	0.00012340	-0.00002242	0.01
7.8800000	0.00010035	0.00012339	-0.00002305	0.01
7.8900000	0.00009972	0.00012339	-0.00002368	0.01
7.9000000	0.00009909	0.00012339	-0.00002430	0.01
7.9100000	0.00009847	0.00012339	-0.00002493	0.01
7.9200000	0.00009784	0.00012339	-0.00002555	0.01
7.9300000	0.00009722	0.00012339	-0.00002617	0.01
7.9400000	0.00009660	0.00012339	-0.00002679	0.01
7.9500000	0.00009598	0.00012339	-0.00002741	0.01
7.9700000	0.00009475	0.00012339	-0.00002864	0.01
7.9800000	0.00009414	0.00012339	-0.00002925	0.01
7.9900000	0.00009353	0.00012339	-0.00002986	0.01
8.0000000	0.00009292	0.00012339	-0.00003047	0.01
8.0100000	0.00009231	0.00012339	-0.00003108	0.01
8.0200000	0.00009171	0.00012339	-0.00003168	0.01
8.0300000	0.00009110	0.00012339	-0.00003229	0.01
8.0400000	0.00009050	0.00012339	-0.00003289	0.01
8.0500000	0.00008990	0.00012339	-0.00003349	0.01
8.0600000	0.00008930	0.00012339	-0.00003409	0.01
8.0700000	0.00008870	0.00012339	-0.00003469	0.01
8.0800000	0.00008810	0.00012339	-0.00003528	0.01
8.0900000	0.00008751	0.00012339	-0.00003588	0.01

8.1000000	0.00008692	0.00012339	-0.00003647	0.01
8.1100000	0.00008633	0.00012339	-0.00003706	0.01
8.1200000	0.00008574	0.00012339	-0.00003765	0.01
8.1300000	0.00008515	0.00012339	-0.00003824	0.01
8.1400000	0.00008456	0.00012339	-0.00003882	0.01
8.1500000	0.00008398	0.00012339	-0.00003941	0.01
8.1600000	0.00008340	0.00012339	-0.00003999	0.01
8.1700000	0.00008281	0.00012339	-0.00004057	0.01
8.1800000	0.00008223	0.00012339	-0.00004115	0.01
8.1900000	0.00008166	0.00012339	-0.00004173	0.01
8.2000000	0.00008108	0.00012339	-0.00004231	0.01
8.2100000	0.00008050	0.00012339	-0.00004288	0.01
8.2200000	0.00007993	0.00012339	-0.00004346	0.01
8.2300000	0.00007936	0.00012339	-0.00004403	0.01
8.2400000	0.00007879	0.00012339	-0.00004460	0.01
8.2500000	0.00007822	0.00012339	-0.00004517	0.01
8.2600000	0.00007765	0.00012339	-0.00004573	0.01
8.2700000	0.00007709	0.00012339	-0.00004630	0.01
8.2800000	0.00007653	0.00012339	-0.00004686	0.01
8.2900000	0.00007596	0.00012339	-0.00004742	0.01
8.3000000	0.00007541	0.00012339	-0.00004798	0.01
8.3100000	0.00007485	0.00012339	-0.00004854	0.01
8.3200000	0.00007429	0.00012339	-0.00004910	0.01
8.3300000	0.00007374	0.00012339	-0.00004965	0.01
8.3400000	0.00007318	0.00012339	-0.00005020	0.01
8.3500000	0.00007263	0.00012339	-0.00005075	0.01
8.3600000	0.00007208	0.00012339	-0.00005130	0.01
8.3700000	0.00007154	0.00012339	-0.00005185	0.01
8.3800000	0.00007099	0.00012339	-0.00005240	0.01
8.3900000	0.00007045	0.00012339	-0.00005294	0.01
8.4000000	0.00006990	0.00012339	-0.00005348	0.01
8.4100000	0.00006936	0.00012339	-0.00005402	0.01
8.4200000	0.00006883	0.00012339	-0.00005456	0.01
8.4300000	0.00006829	0.00012339	-0.00005510	0.01
8.4400000	0.00006776	0.00012339	-0.00005563	0.01
8.4500000	0.00006722	0.00012339	-0.00005616	0.01
8.4600000	0.00006669	0.00012339	-0.00005669	0.01
8.4700000	0.00006616	0.00012339	-0.00005722	0.01
8.4800000	0.00006564	0.00012339	-0.00005775	0.01
8.4900000	0.00006511	0.00012339	-0.00005827	0.01
8.5000000	0.00006459	0.00012339	-0.00005879	0.01
8.5100000	0.00006407	0.00012339	-0.00005932	0.01
8.5200000	0.00006355	0.00012339	-0.00005983	0.01
8.5300000	0.00006304	0.00012339	-0.00006035	0.01
8.5400000	0.00006252	0.00012339	-0.00006086	0.01
8.5500000	0.00006201	0.00012339	-0.00006138	0.01
8.5600000	0.00006150	0.00012339	-0.00006189	0.01
8.5700000	0.00006099	0.00012339	-0.00006239	0.01
8.5900000	0.00005998	0.00012339	-0.00006340	0.01
8.6000000	0.00005948	0.00012339	-0.00006390	0.01
8.6100000	0.00005898	0.00012339	-0.00006440	0.01
8.6200000	0.00005849	0.00012339	-0.00006490	0.01

8.6300000	0.00005799	0.00012339	-0.00006540	0.01
8.6400000	0.00005750	0.00012339	-0.00006589	0.01
8.6500000	0.00005701	0.00012339	-0.00006638	0.01
8.6600000	0.00005652	0.00012339	-0.00006687	0.01
8.6700000	0.00005603	0.00012339	-0.00006735	0.01
8.6800000	0.00005555	0.00012339	-0.00006783	0.01
8.6900000	0.00005507	0.00012339	-0.00006832	0.01
8.7000000	0.00005459	0.00012339	-0.00006879	0.01
8.7100000	0.00005411	0.00012339	-0.00006927	0.01
8.7200000	0.00005364	0.00012339	-0.00006975	0.01
8.7300000	0.00005317	0.00012339	-0.00007022	0.01
8.7400000	0.00005270	0.00012339	-0.00007069	0.01
8.7500000	0.00005223	0.00012339	-0.00007115	0.01
8.7600000	0.00005177	0.00012339	-0.00007162	0.01
8.7700000	0.00005131	0.00012339	-0.00007208	0.01
8.7800000	0.00005085	0.00012339	-0.00007254	0.01
8.7900000	0.00005039	0.00012339	-0.00007300	0.01
8.8000000	0.00004993	0.00012339	-0.00007345	0.01
8.8100000	0.00004948	0.00012339	-0.00007390	0.01
8.8200000	0.00004903	0.00012339	-0.00007435	0.01
8.8300000	0.00004858	0.00012339	-0.00007480	0.01
8.8400000	0.00004814	0.00012339	-0.00007525	0.01
8.8500000	0.00004770	0.00012339	-0.00007569	0.01
8.8600000	0.00004726	0.00012339	-0.00007613	0.01
8.8700000	0.00004682	0.00012339	-0.00007657	0.01
8.8800000	0.00004638	0.00012339	-0.00007700	0.01
8.8900000	0.00004595	0.00012339	-0.00007743	0.01
8.9000000	0.00004552	0.00012339	-0.00007786	0.01
8.9100000	0.00004509	0.00012339	-0.00007829	0.01
8.9200000	0.00004467	0.00012339	-0.00007872	0.01
8.9300000	0.00004425	0.00012339	-0.00007914	0.01
8.9400000	0.00004383	0.00012339	-0.00007956	0.01
8.9500000	0.00004341	0.00012339	-0.00007998	0.01
8.9600000	0.00004300	0.00012339	-0.00008039	0.01
8.9700000	0.00004258	0.00012339	-0.00008080	0.01
8.9800000	0.00004217	0.00012339	-0.00008121	0.01
8.9900000	0.00004177	0.00012339	-0.00008162	0.01
9.0000000	0.00004136	0.00012339	-0.00008202	0.01
9.0100000	0.00004096	0.00012339	-0.00008242	0.01
9.0200000	0.00004056	0.00012339	-0.00008282	0.01
9.0300000	0.00004017	0.00012339	-0.00008322	0.01
9.0400000	0.00003977	0.00012339	-0.00008361	0.01
9.0500000	0.00003938	0.00012339	-0.00008400	0.01
9.0600000	0.00003899	0.00012339	-0.00008439	0.01
9.0700000	0.00003861	0.00012339	-0.00008478	0.01
9.0800000	0.00003822	0.00012339	-0.00008516	0.01
9.0900000	0.00003784	0.00012339	-0.00008554	0.01
9.1000000	0.00003747	0.00012339	-0.00008592	0.01
9.1100000	0.00003709	0.00012339	-0.00008630	0.01
9.1200000	0.00003672	0.00012339	-0.00008667	0.01
9.1300000	0.00003635	0.00012339	-0.00008704	0.01
9.1400000	0.00003598	0.00012339	-0.00008741	0.01

9.1500000	0.00003561	0.00012339	-0.00008777	0.01
9.1600000	0.00003525	0.00012339	-0.00008813	0.01
9.1700000	0.00003489	0.00012339	-0.00008849	0.01
9.1800000	0.00003454	0.00012339	-0.00008885	0.01
9.1900000	0.00003418	0.00012339	-0.00008920	0.01
9.2000000	0.00003383	0.00012339	-0.00008956	0.01
9.2100000	0.00003348	0.00012339	-0.00008990	0.01
9.2200000	0.00003313	0.00012339	-0.00009025	0.01
9.2300000	0.00003279	0.00012339	-0.00009059	0.01
9.2400000	0.00003245	0.00012339	-0.00009094	0.01
9.2500000	0.00003211	0.00012339	-0.00009127	0.01
9.2600000	0.00003177	0.00012339	-0.00009161	0.01
9.2700000	0.00003144	0.00012339	-0.00009194	0.01
9.2800000	0.00003111	0.00012339	-0.00009227	0.01
9.2900000	0.00003078	0.00012339	-0.00009260	0.01
9.3000000	0.00003046	0.00012339	-0.00009293	0.01
9.3100000	0.00003013	0.00012339	-0.00009325	0.01
9.3200000	0.00002981	0.00012339	-0.00009357	0.01
9.3300000	0.00002950	0.00012339	-0.00009389	0.01
9.3400000	0.00002918	0.00012339	-0.00009421	0.01
9.3500000	0.00002887	0.00012339	-0.00009452	0.01
9.3600000	0.00002856	0.00012339	-0.00009483	0.01
9.3700000	0.00002825	0.00012339	-0.00009514	0.01
9.3800000	0.00002794	0.00012339	-0.00009544	0.01
9.3900000	0.00002764	0.00012339	-0.00009574	0.01
9.4000000	0.00002734	0.00012339	-0.00009604	0.01
9.4100000	0.00002705	0.00012339	-0.00009634	0.01
9.4200000	0.00002675	0.00012339	-0.00009664	0.01
9.4300000	0.00002646	0.00012339	-0.00009693	0.01
9.4400000	0.00002617	0.00012339	-0.00009722	0.01
9.4500000	0.00002588	0.00012339	-0.00009750	0.01
9.4600000	0.00002560	0.00012339	-0.00009779	0.01
9.4700000	0.00002531	0.00012339	-0.00009807	0.01
9.4800000	0.00002503	0.00012339	-0.00009835	0.01
9.4900000	0.00002476	0.00012339	-0.00009863	0.01
9.5000000	0.00002448	0.00012339	-0.00009890	0.01
9.5100000	0.00002421	0.00012339	-0.00009918	0.01
9.5200000	0.00002394	0.00012339	-0.00009945	0.01
9.5300000	0.00002367	0.00012339	-0.00009971	0.01
9.5400000	0.00002341	0.00012339	-0.00009998	0.01
9.5500000	0.00002314	0.00012339	-0.00010024	0.01
9.5600000	0.00002288	0.00012339	-0.00010050	0.01
9.5700000	0.00002263	0.00012339	-0.00010076	0.01
9.5800000	0.00002237	0.00012339	-0.00010102	0.01
9.5900000	0.00002212	0.00012339	-0.00010127	0.01
9.6000000	0.00002187	0.00012339	-0.00010152	0.01
9.6100000	0.00002162	0.00012339	-0.00010177	0.01
9.6200000	0.00002137	0.00012339	-0.00010201	0.01
9.6300000	0.00002113	0.00012339	-0.00010226	0.01
9.6400000	0.00002089	0.00012339	-0.00010250	0.01
9.6500000	0.00002065	0.00012339	-0.00010274	0.01
9.6600000	0.00002041	0.00012339	-0.00010298	0.01

9.6700000	0.00002017	0.00012339	-0.00010321	0.01
9.6800000	0.00001994	0.00012339	-0.00010344	0.01
9.6900000	0.00001971	0.00012339	-0.00010367	0.01
9.7000000	0.00001948	0.00012339	-0.00010390	0.01
9.7100000	0.00001926	0.00012339	-0.00010413	0.01
9.7200000	0.00001904	0.00012339	-0.00010435	0.01
9.7300000	0.00001881	0.00012339	-0.00010457	0.01
9.7400000	0.00001860	0.00012339	-0.00010479	0.01
9.7500000	0.00001838	0.00012339	-0.00010501	0.01
9.7600000	0.00001816	0.00012339	-0.00010522	0.01
9.7700000	0.00001795	0.00012339	-0.00010543	0.01
9.7800000	0.00001774	0.00012339	-0.00010564	0.01
9.7900000	0.00001753	0.00012339	-0.00010585	0.01
9.8000000	0.00001733	0.00012339	-0.00010606	0.01
9.8100000	0.00001712	0.00012339	-0.00010626	0.01
9.8200000	0.00001692	0.00012339	-0.00010646	0.01
9.8300000	0.00001672	0.00012339	-0.00010666	0.01
9.8400000	0.00001653	0.00012339	-0.00010686	0.01
9.8500000	0.00001633	0.00012339	-0.00010706	0.01
9.8600000	0.00001614	0.00012339	-0.00010725	0.01
9.8700000	0.00001595	0.00012339	-0.00010744	0.01
9.8800000	0.00001576	0.00012339	-0.00010763	0.01
9.8900000	0.00001557	0.00012339	-0.00010782	0.01
9.9000000	0.00001538	0.00012339	-0.00010800	0.01
9.9100000	0.00001520	0.00012339	-0.00010819	0.01
9.9200000	0.00001502	0.00012339	-0.00010837	0.01
9.9300000	0.00001484	0.00012339	-0.00010855	0.01
9.9400000	0.00001466	0.00012339	-0.00010872	0.01
9.9500000	0.00001449	0.00012339	-0.00010890	0.01
9.9600000	0.00001431	0.00012339	-0.00010907	0.01
9.9700000	0.00001414	0.00012339	-0.00010925	0.01
9.9800000	0.00001397	0.00012339	-0.00010942	0.01
9.9900000	0.00001380	0.00012339	-0.00010958	0.01
10.0000000	0.00001364	0.00012339	-0.00010975	0.01
10.0100000	0.00001347	0.00012339	-0.00010991	0.01
10.0200000	0.00001331	0.00012339	-0.00011008	0.01
10.0300000	0.00001315	0.00012339	-0.00011024	0.01
10.0400000	0.00001299	0.00012339	-0.00011040	0.01
10.0500000	0.00001283	0.00012339	-0.00011055	0.01
10.0600000	0.00001268	0.00012339	-0.00011071	0.01
10.0700000	0.00001252	0.00012339	-0.00011086	0.01
10.0800000	0.00001237	0.00012339	-0.00011101	0.01
10.0900000	0.00001222	0.00012339	-0.00011116	0.01
10.1000000	0.00001207	0.00012339	-0.00011131	0.01
10.1100000	0.00001193	0.00012339	-0.00011146	0.01
10.1200000	0.00001178	0.00012339	-0.00011160	0.01
10.1300000	0.00001164	0.00012339	-0.00011175	0.01
10.1400000	0.00001150	0.00012339	-0.00011189	0.01
10.1500000	0.00001136	0.00012339	-0.00011203	0.01
10.1600000	0.00001122	0.00012339	-0.00011217	0.01
10.1700000	0.00001108	0.00012339	-0.00011230	0.01
10.1800000	0.00001095	0.00012339	-0.00011244	0.01

10.1900000	0.00001081	0.00012339	-0.00011257	0.01
10.2000000	0.00001068	0.00012339	-0.00011270	0.01
10.2100000	0.00001055	0.00012339	-0.00011283	0.01
10.2200000	0.00001042	0.00012339	-0.00011296	0.01
10.2300000	0.00001029	0.00012339	-0.00011309	0.01
10.2400000	0.00001017	0.00012339	-0.00011322	0.01
10.2500000	0.00001004	0.00012339	-0.00011334	0.01
10.2600000	0.00000992	0.00012339	-0.00011346	0.01
10.2700000	0.00000980	0.00012339	-0.00011359	0.01
10.2800000	0.00000968	0.00012339	-0.00011371	0.01
10.2900000	0.00000956	0.00012339	-0.00011382	0.01
10.3000000	0.00000945	0.00012339	-0.00011394	0.01
10.3100000	0.00000933	0.00012339	-0.00011406	0.01
10.3200000	0.00000922	0.00012339	-0.00011417	0.01
10.3300000	0.00000910	0.00012339	-0.00011428	0.01
10.3400000	0.00000899	0.00012339	-0.00011439	0.01
10.3500000	0.00000888	0.00012339	-0.00011450	0.01
10.3600000	0.00000877	0.00012339	-0.00011461	0.01
10.3700000	0.00000867	0.00012339	-0.00011472	0.01
10.3800000	0.00000856	0.00012339	-0.00011483	0.01
10.3900000	0.00000845	0.00012339	-0.00011493	0.01
10.4000000	0.00000835	0.00012339	-0.00011503	0.01
10.4100000	0.00000825	0.00012339	-0.00011514	0.01
10.4200000	0.00000815	0.00012339	-0.00011524	0.01
10.4300000	0.00000805	0.00012339	-0.00011534	0.01
10.4400000	0.00000795	0.00012339	-0.00011543	0.01
10.4500000	0.00000785	0.00012339	-0.00011553	0.01
10.4600000	0.00000776	0.00012339	-0.00011563	0.01
10.4700000	0.00000766	0.00012339	-0.00011572	0.01
10.4800000	0.00000757	0.00012339	-0.00011581	0.01
10.4900000	0.00000748	0.00012339	-0.00011591	0.01
10.5000000	0.00000739	0.00012339	-0.00011600	0.01
10.5100000	0.00000730	0.00012339	-0.00011609	0.01
10.5200000	0.00000721	0.00012339	-0.00011618	0.01
10.5300000	0.00000712	0.00012339	-0.00011626	0.01
10.5400000	0.00000703	0.00012339	-0.00011635	0.01
10.5500000	0.00000695	0.00012339	-0.00011644	0.01
10.5600000	0.00000687	0.00012339	-0.00011652	0.01
10.5700000	0.00000678	0.00012339	-0.00011660	0.01
10.5800000	0.00000670	0.00012339	-0.00011669	0.01
10.5900000	0.00000662	0.00012339	-0.00011677	0.01
10.6000000	0.00000654	0.00012339	-0.00011685	0.01

SUM OF SQUARES OF ERRORS=0.3367D-05 AND THE STANDARD DEVIATION IS=0.0001

	OLD PARM	NEW PARM	DELTA PARM	ST. ERR.
r_e	0.153926910D+01	0.153926910D+01	0.00000D+00	0.16120D-04
C[3]	-0.362604669D+01	-0.362604669D+01	0.00000D+00	0.20761D-03
C[4]	0.629953356D+00	0.629953356D+00	0.34417D-14	0.97797D-03
C[5]	0.476010408D+01	0.476010408D+01	-0.17764D-14	0.24729D-02

C[6]	0.774229721D+00	0.774229721D+00	-0.20650D-13	0.56397D-02
C[7]	-0.606380043D+01	-0.606380043D+01	0.21316D-13	0.11542D-01
C[8]	-0.154742229D+01	-0.154742229D+01	0.27311D-13	0.71077D-02
C[9]	0.709821461D+01	0.709821461D+01	-0.46185D-13	0.19156D-01
C[10]	-0.304172389D+01	-0.304172389D+01	0.16431D-13	0.94504D-02
V0	0.101661491D+01	0.101661491D+01	0.00000D+00	0.25048D-05

(a=0.5818082, a1=0.0210674, a2=0.2 fixed after a preliminary determination)

```
  y=1-exp{a*(r-r_e)+a1*(r-r_e)+a2*(r-r_e)^2
EQMF=V0+C[3]*y^2+C[4]*y^3+C[5]*y^4+C[6]*y^5+C[7]*y^6+C[8]*y^7+C[9]*y^8+C[10]*y^9
```

Table S7. The reproduction of the reference transition moments <v,J|Θ|v',J'> (in a.u.) QTM of H_2 by the electric quadrupole moment function obtained by fitting to the most accurate experimental transition moments using the theoretical MRCI quadrupole moment function of HF evaluated by Harrison (private communication).
If not stated otherwise, the reference data are taken from [Campargue A. et al:PCCP 2012,14,802 (Table 3)]. Remaining "Kassi" and "Fleur" data are taken from [Kassi S. and Campargue A.:JMS 2014,300,55 (Table 1)] and [Fleurbaey et al:PCCP 2022,25,14749 (Table 2)], respectively.
Δ=(<v,J|Θ|v',J'>_ref - <v,J|Θ|v',J'>_calc)/<v,J|Θ|v',J'>_ref.

M	v"	J"	v`	J`	QTM-Ref	QTM-Calc	Ref-Calc	Δ
1	0	0	0	2	-0.484805D+00	-0.482589D+00	-0.00221641	0.0046
2	0	1	0	3	-0.486961D+00	-0.484703D+00	-0.00225812	0.0046
3	0	1	0	3	-0.408184D+00	-0.484703D+00	0.07651888	-0.1875
4	0	2	0	4	-0.490047D+00	-0.487864D+00	-0.00218283	0.0045
5	0	2	0	4	-0.518344D+00	-0.487864D+00	-0.03047983	0.0588
6	0	3	0	5	-0.494402D+00	-0.492060D+00	-0.00234151	0.0047
7	0	3	0	5	-0.490309D+00	-0.492060D+00	0.00175149	-0.0036
8	0	3	0	5	-0.493819D+00	-0.492060D+00	-0.00175851	0.0036
9	0	4	0	6	-0.499552D+00	-0.497276D+00	-0.00227582	0.0046
10	0	5	0	7	-0.505771D+00	-0.503492D+00	-0.00227877	0.0045
11	0	4	1	4	0.884649D-01	0.881612D-01	0.00030368	0.0034
12	0	3	1	3	0.881836D-01	0.878877D-01	0.00029592	0.0034
13	0	3	1	3	0.814211D-01	0.878877D-01	-0.00646658	-0.0794
14	0	3	1	3	0.917725D-01	0.878877D-01	0.00388482	0.0423
15	0	2	1	2	0.879714D-01	0.876777D-01	0.00029374	0.0033
16	0	2	1	2	0.829292D-01	0.876777D-01	-0.00474846	-0.0573
17	0	2	1	2	0.891959D-01	0.876777D-01	0.00151824	0.0170
18	0	1	1	1	0.878263D-01	0.875353D-01	0.00029101	0.0033
19	0	1	1	1	0.771491D-01	0.875353D-01	-0.01038619	-0.1346
20	0	1	1	1	0.896092D-01	0.875353D-01	0.00207391	0.0231
21	0	1	1	1	0.772308D-01	0.875353D-01	-0.01030449	-0.1334
22	0	0	1	2	0.782057D-01	0.779523D-01	0.00025335	0.0032
23	0	0	1	2	0.768782D-01	0.779523D-01	-0.00107415	-0.0140

24	0	0	1	2	0.842174D-01	0.779523D-01	0.00626505	0.0744	
25	0	0	1	2	0.714522D-01	0.779523D-01	-0.00650015	-0.0910	
26	0	1	1	3	0.719290D-01	0.717006D-01	0.00022842	0.0032	
27	0	1	1	3	0.737556D-01	0.717006D-01	0.00205502	0.0279	
28	0	1	1	3	0.762580D-01	0.717006D-01	0.00455742	0.0598	
29	0	1	1	3	0.676812D-01	0.717006D-01	-0.00401938	-0.0594	
30	0	2	1	4	0.657416D-01	0.655369D-01	0.00020475	0.0031	
31	0	2	1	4	0.701802D-01	0.655369D-01	0.00464335	0.0662	
32	0	2	1	4	0.830203D-01	0.655369D-01	0.01748345	0.2106	
33	0	3	1	5	0.596421D-01	0.594603D-01	0.00018183	0.0030	
34	0	3	1	5	0.611139D-01	0.594603D-01	0.00165363	0.0271	
35	0	3	1	5	0.600524D-01	0.594603D-01	0.00059213	0.0099	
36	0	5	2	3	0.885888D-02	0.890380D-02	-0.00004492	-0.0051	
37	0	5	2	3	0.849647D-02	0.890380D-02	-0.00040733	-0.0479	
38	0	4	2	2	0.948704D-02	0.952646D-02	-0.00003942	-0.0042	
39	0	4	2	2	0.914795D-02	0.952646D-02	-0.00037851	-0.0414	
40	0	3	2	1	0.100612D-01	0.100954D-01	-0.00003420	-0.0034	
41	0	3	2	1	0.100912D-01	0.100954D-01	-0.00000420	-0.0004	
42	0	3	2	1	0.918011D-02	0.100954D-01	-0.00091529	-0.0997	
43	0	2	2	0	0.105702D-01	0.106008D-01	-0.00003058	-0.0029	
44	0	2	2	0	0.105764D-01	0.106008D-01	-0.00002438	-0.0023	
45	0	2	2	0	0.999938D-02	0.106008D-01	-0.00060140	-0.0601	
46	0	5	2	5	0.117121D-01	0.117491D-01	-0.00003699	-0.0032	
47	0	5	2	5	0.118255D-01	0.117491D-01	0.00007641	0.0065	
48	0	4	2	4	0.115817D-01	0.115695D-01	0.00001216	0.0010	Kassi
49	0	4	2	4	0.116054D-01	0.115695D-01	0.00003586	0.0031	Fleur
50	0	3	2	3	0.113954D-01	0.114255D-01	-0.00003008	-0.0026	Kassi
51	0	3	2	3	0.111010D-01	0.114255D-01	-0.00032448	-0.0292	
52	0	3	2	3	0.114295D-01	0.114255D-01	0.00000402	0.0004	Kassi
53	0	3	2	3	0.114507D-01	0.114255D-01	0.00002522	0.0022	Fleur
54	0	2	2	2	0.112886D-01	0.113170D-01	-0.00002843	-0.0025	
55	0	2	2	2	0.111348D-01	0.113170D-01	-0.00018223	-0.0164	
56	0	2	2	2	0.113543D-01	0.113170D-01	0.00003727	0.0033	Kassi
57	0	2	2	2	0.113044D-01	0.113170D-01	-0.00001263	-0.0011	Fleur
58	0	1	2	1	0.112168D-01	0.112445D-01	-0.00002767	-0.0025	
59	0	1	2	1	0.118557D-01	0.112445D-01	0.00061123	0.0516	
60	0	1	2	1	0.110690D-01	0.112445D-01	-0.00017547	-0.0159	
61	0	1	2	1	0.114814D-01	0.112445D-01	0.00023693	0.0206	
62	0	1	2	1	0.112360D-01	0.112445D-01	-0.00000847	-0.0008	Kassi
63	0	1	2	1	0.112257D-01	0.112445D-01	-0.00001877	-0.0017	Fleur
64	0	0	2	2	0.116326D-01	0.116598D-01	-0.00002717	-0.0023	
65	0	0	2	2	0.115411D-01	0.116598D-01	-0.00011867	-0.0103	
66	0	0	2	2	0.116251D-01	0.116598D-01	-0.00003467	-0.0030	Fleur
67	0	1	2	3	0.118154D-01	0.118436D-01	-0.00002821	-0.0024	
68	0	1	2	3	0.117919D-01	0.118436D-01	-0.00005171	-0.0044	
69	0	1	2	3	0.117507D-01	0.118436D-01	-0.00009291	-0.0079	
70	0	1	2	3	0.117926D-01	0.118436D-01	-0.00005101	-0.0043	
71	0	1	2	3	0.118243D-01	0.118436D-01	-0.00001931	-0.0016	Fleur
72	0	2	2	4	0.119085D-01	0.119390D-01	-0.00003047	-0.0026	
73	0	3	3	3	0.164247D-02	0.163542D-02	0.00000705	0.0043	
74	0	3	3	3	0.138860D-02	0.163542D-02	-0.00024682	-0.1777	
75	0	2	3	2	0.161950D-02	0.161044D-02	0.00000906	0.0056	

76	0	2	3	2	0.139816D-02	0.161044D-02	-0.00021228	-0.1518
77	0	1	3	1	0.160404D-02	0.159329D-02	0.00001075	0.0067
78	0	1	3	1	0.131248D-02	0.159329D-02	-0.00028081	-0.2140
79	0	0	3	2	0.193176D-02	0.192239D-02	0.00000937	0.0048
80	0	0	3	2	0.174567D-02	0.192239D-02	-0.00017672	-0.1012
81	0	0	3	2	0.164918D-02	0.192239D-02	-0.00027321	-0.1657
82	0	1	3	3	0.213258D-02	0.212609D-02	0.00000649	0.0030
83	0	1	3	3	0.203621D-02	0.212609D-02	-0.00008988	-0.0441
84	0	1	3	3	0.204524D-02	0.212609D-02	-0.00008085	-0.0395
85	0	1	3	3	0.211174D-02	0.212609D-02	-0.00001435	-0.0068
86	0	2	3	4	0.231443D-02	0.231080D-02	0.00000363	0.0016
87	0	2	3	4	0.233435D-02	0.231080D-02	0.00002355	0.0101
88	0	2	3	4	0.224478D-02	0.231080D-02	-0.00006602	-0.0294
89	0	3	3	5	0.247511D-02	0.247401D-02	0.00000110	0.0004
90	0	3	3	5	0.232074D-02	0.247401D-02	-0.00015327	-0.0660
91	0	3	3	5	0.233799D-02	0.247401D-02	-0.00013602	-0.0582
92	0	0	4	2	0.430748D-03	0.420488D-03	0.00001026	0.0238
93	0	0	4	2	0.335671D-03	0.420488D-03	-0.00008482	-0.2527
94	0	0	4	2	0.344055D-03	0.420488D-03	-0.00007643	-0.2222
95	0	1	4	3	0.511498D-03	0.504835D-03	0.00000666	0.0130
96	0	1	4	3	0.481637D-03	0.504835D-03	-0.00002320	-0.0482
97	0	1	4	3	0.501742D-03	0.504835D-03	-0.00000309	-0.0062
98	0	1	4	3	0.393813D-03	0.504835D-03	-0.00011102	-0.2819
99	0	1	4	3	0.473356D-03	0.504835D-03	-0.00003148	-0.0665
100	0	2	4	4	0.589213D-03	0.586362D-03	0.00000285	0.0048
101	0	2	4	4	0.501817D-03	0.586362D-03	-0.00008455	-0.1685
102	0	3	4	5	0.662795D-03	0.662429D-03	0.00000037	0.0006
103	0	3	4	5	0.604505D-03	0.662429D-03	-0.00005792	-0.0958
104	0	1	5	3	0.154749D-03	0.158834D-03	-0.00000408	-0.0264
105	0	1	5	3	0.127863D-03	0.158834D-03	-0.00003097	-0.2422

SUM OF SQUARES OF ERRORS=0.69572523D-03 AND THE STANDARD DEVIATION IS=0.0073155537

CORRELATION MATRIX

CORR(1, 2)=-0.3214560
CORR(1, 3)=-0.8662714
CORR(1, 4)=-0.6026219
CORR(2, 3)=-0.1411585
CORR(2, 4)= 0.6292050
CORR(3, 4)= 0.1736690

Jenc-Dereduction used to fit PEC

	OLD PARM	NEW PARM	DELTA PARM	ST. ERR.
R-eq	0.160920185D+01	0.160920209D+01	0.24287E-06	0.94681E-02
RHO-ij	0.751977314D+00	0.751977009D+00	-0.30453E-06	0.53218E-02
Dis.Bar	-0.104672596D+01	-0.104672596D+01	0.54623E-08	0.10747E-01

Alpha	0.777864957D+00	0.777864763D+00	-0.19434E-06	0.38808E-02
	Beta fixed at 0.81			

Table S8. The reproduction of the quadrupole transition moments QTM-ai=<v"J"|Θ|v',J'>(in a.u.) evaluated using the ab initio quadrupole moment function of Somogyi et al.J. Chem. Phys. 2021,155,214303) by its approximant evaluated using exponential polynomials.
Δ=(QTM-ai - QTM-expon)/QTM-ai.

M	v"	J"	v'	J'	QTM-ai	QTM-expon	ai-expon	Δ
1	0	0	0	0	0.174728D+01	0.174729D+01	-0.00000998	-0.00001
2	0	0	0	1	0.174756D+01	0.174757D+01	-0.00000999	-0.00001
3	0	1	0	2	0.174841D+01	0.174842D+01	-0.00001002	-0.00001
4	0	2	0	3	0.174982D+01	0.174983D+01	-0.00001005	-0.00001
5	0	3	0	4	0.175180D+01	0.175181D+01	-0.00001011	-0.00001
6	0	4	0	5	0.175435D+01	0.175436D+01	-0.00001019	-0.00001
7	0	5	0	6	0.175746D+01	0.175747D+01	-0.00001028	-0.00001
8	0	6	0	7	0.176115D+01	0.176116D+01	-0.00001038	-0.00001
9	0	7	0	8	0.176542D+01	0.176543D+01	-0.00001048	-0.00001
10	0	8	0	9	0.177026D+01	0.177027D+01	-0.00001060	-0.00001
11	0	9	0	10	0.177569D+01	0.177570D+01	-0.00001073	-0.00001
12	0	1	0	0	0.174756D+01	0.174757D+01	-0.00000999	-0.00001
13	0	2	0	1	0.174841D+01	0.174842D+01	-0.00001002	-0.00001
14	0	3	0	2	0.174982D+01	0.174983D+01	-0.00001005	-0.00001
15	0	4	0	3	0.175180D+01	0.175181D+01	-0.00001011	-0.00001
16	0	5	0	4	0.175435D+01	0.175436D+01	-0.00001019	-0.00001
17	0	6	0	5	0.175746D+01	0.175747D+01	-0.00001028	-0.00001
18	0	7	0	6	0.176115D+01	0.176116D+01	-0.00001038	-0.00001
19	0	8	0	7	0.176542D+01	0.176543D+01	-0.00001048	-0.00001
20	0	9	0	8	0.177026D+01	0.177027D+01	-0.00001060	-0.00001
21	0	8	0	9	0.177026D+01	0.177027D+01	-0.00001060	-0.00001
22	0	10	0	10	0.177875D+01	0.177877D+01	-0.00001078	-0.00001
23	0	0	1	0	0.195280D+00	0.195285D+00	-0.00000481	-0.00002
24	0	0	1	1	0.192733D+00	0.192738D+00	-0.00000478	-0.00002
25	0	1	1	2	0.190244D+00	0.190249D+00	-0.00000470	-0.00002
26	0	2	1	3	0.187811D+00	0.187816D+00	-0.00000459	-0.00002
27	0	3	1	4	0.185432D+00	0.185437D+00	-0.00000443	-0.00002
28	0	4	1	5	0.183105D+00	0.183109D+00	-0.00000423	-0.00002
29	0	5	1	6	0.180826D+00	0.180830D+00	-0.00000397	-0.00002
30	0	6	1	7	0.178593D+00	0.178597D+00	-0.00000364	-0.00002
31	0	7	1	8	0.176405D+00	0.176408D+00	-0.00000325	-0.00002
32	0	8	1	9	0.174257D+00	0.174260D+00	-0.00000277	-0.00002
33	0	9	1	10	0.172148D+00	0.172150D+00	-0.00000219	-0.00001
34	0	1	1	0	0.197887D+00	0.197892D+00	-0.00000481	-0.00002
35	0	2	1	1	0.200557D+00	0.200562D+00	-0.00000477	-0.00002
36	0	3	1	2	0.203292D+00	0.203297D+00	-0.00000469	-0.00002
37	0	4	1	3	0.206094D+00	0.206099D+00	-0.00000456	-0.00002
38	0	5	1	4	0.208965D+00	0.208970D+00	-0.00000439	-0.00002

39	0	6	1	5	0.211908D+00	0.211912D+00	-0.00000416	-0.00002
40	0	7	1	6	0.214924D+00	0.214927D+00	-0.00000387	-0.00002
41	0	8	1	7	0.218015D+00	0.218019D+00	-0.00000350	-0.00002
42	0	9	1	8	0.221184D+00	0.221187D+00	-0.00000306	-0.00001
43	0	8	1	9	0.174257D+00	0.174260D+00	-0.00000277	-0.00002
44	0	10	1	10	0.198574D+00	0.198576D+00	-0.00000204	-0.00001
45	0	0	2	0	0.169354D-01	0.169483D-01	-0.00001287	-0.00076
46	0	0	2	1	0.169985D-01	0.170114D-01	-0.00001292	-0.00076
47	0	1	2	2	0.170706D-01	0.170836D-01	-0.00001308	-0.00077
48	0	2	2	3	0.171516D-01	0.171649D-01	-0.00001333	-0.00078
49	0	3	2	4	0.172417D-01	0.172554D-01	-0.00001369	-0.00079
50	0	4	2	5	0.173409D-01	0.173551D-01	-0.00001415	-0.00082
51	0	5	2	6	0.174494D-01	0.174641D-01	-0.00001470	-0.00084
52	0	6	2	7	0.175671D-01	0.175825D-01	-0.00001535	-0.00087
53	0	7	2	8	0.176943D-01	0.177104D-01	-0.00001609	-0.00091
54	0	8	2	9	0.178311D-01	0.178480D-01	-0.00001691	-0.00095
55	0	9	2	10	0.179777D-01	0.179955D-01	-0.00001781	-0.00099
56	0	1	2	0	0.168813D-01	0.168943D-01	-0.00001292	-0.00077
57	0	2	2	1	0.168362D-01	0.168493D-01	-0.00001308	-0.00078
58	0	3	2	2	0.168001D-01	0.168135D-01	-0.00001334	-0.00079
59	0	4	2	3	0.167731D-01	0.167868D-01	-0.00001371	-0.00082
60	0	5	2	4	0.167553D-01	0.167695D-01	-0.00001418	-0.00085
61	0	6	2	5	0.167468D-01	0.167616D-01	-0.00001475	-0.00088
62	0	7	2	6	0.167478D-01	0.167632D-01	-0.00001542	-0.00092
63	0	8	2	7	0.167585D-01	0.167746D-01	-0.00001618	-0.00097
64	0	9	2	8	0.167790D-01	0.167960D-01	-0.00001705	-0.00102
65	0	8	2	9	0.178311D-01	0.178480D-01	-0.00001691	-0.00095
66	0	10	2	10	0.177370D-01	0.177554D-01	-0.00001838	-0.00104
67	0	0	3	0	0.143774D-02	0.142306D-02	0.00001468	0.01021
68	0	0	3	1	0.146986D-02	0.145526D-02	0.00001460	0.00994
69	0	1	3	2	0.150323D-02	0.148876D-02	0.00001447	0.00962
70	0	2	3	3	0.153792D-02	0.152365D-02	0.00001427	0.00928
71	0	3	3	4	0.157401D-02	0.156001D-02	0.00001400	0.00890
72	0	4	3	5	0.161157D-02	0.159791D-02	0.00001366	0.00848
73	0	5	3	6	0.165069D-02	0.163745D-02	0.00001324	0.00802
74	0	6	3	7	0.169145D-02	0.167871D-02	0.00001273	0.00753
75	0	7	3	8	0.173393D-02	0.172181D-02	0.00001213	0.00699
76	0	8	3	9	0.177825D-02	0.176684D-02	0.00001141	0.00642
77	0	9	3	10	0.182448D-02	0.181391D-02	0.00001057	0.00579
78	0	1	3	0	0.140681D-02	0.139211D-02	0.00001470	0.01045
79	0	2	3	1	0.137700D-02	0.136233D-02	0.00001466	0.01065
80	0	3	3	2	0.134824D-02	0.133368D-02	0.00001456	0.01080
81	0	4	3	3	0.132048D-02	0.130608D-02	0.00001440	0.01090
82	0	5	3	4	0.129366D-02	0.127949D-02	0.00001416	0.01095
83	0	6	3	5	0.126772D-02	0.125387D-02	0.00001386	0.01093
84	0	7	3	6	0.124262D-02	0.122916D-02	0.00001346	0.01084
85	0	8	3	7	0.121831D-02	0.120533D-02	0.00001298	0.01065
86	0	9	3	8	0.119472D-02	0.118233D-02	0.00001239	0.01037
87	0	8	3	9	0.177825D-02	0.176684D-02	0.00001141	0.00642
88	0	10	3	10	0.154456D-02	0.153385D-02	0.00001071	0.00693
89	0	0	4	0	0.119778D-03	0.103789D-03	0.00001599	0.13349
90	0	0	4	1	0.126614D-03	0.110584D-03	0.00001603	0.12661

91	0	1	4	2	0.133747D-03	0.117622D-03	0.00001612	0.12056
92	0	2	4	3	0.141198D-03	0.124929D-03	0.00001627	0.11522
93	0	3	4	4	0.148992D-03	0.132533D-03	0.00001646	0.11047
94	0	4	4	5	0.157153D-03	0.140464D-03	0.00001669	0.10620
95	0	5	4	6	0.165707D-03	0.148755D-03	0.00001695	0.10230
96	0	6	4	7	0.174679D-03	0.157442D-03	0.00001724	0.09868
97	0	7	4	8	0.184098D-03	0.166562D-03	0.00001754	0.09525
98	0	8	4	9	0.193995D-03	0.176157D-03	0.00001784	0.09195
99	0	9	4	10	0.204399D-03	0.186270D-03	0.00001813	0.08869
100	0	1	4	0	0.113217D-03	0.972147D-04	0.00001600	0.14134
101	0	2	4	1	0.106910D-03	0.908388D-04	0.00001607	0.15032
102	0	3	4	2	0.100836D-03	0.846423D-04	0.00001619	0.16059
103	0	4	4	3	0.949746D-04	0.786082D-04	0.00001637	0.17232
104	0	5	4	4	0.893073D-04	0.727211D-04	0.00001659	0.18572
105	0	6	4	5	0.838150D-04	0.669676D-04	0.00001685	0.20101
106	0	7	4	6	0.784793D-04	0.613360D-04	0.00001714	0.21844
107	0	8	4	7	0.732824D-04	0.558164D-04	0.00001747	0.23834
108	0	9	4	8	0.682070D-04	0.504003D-04	0.00001781	0.26107
109	0	8	4	9	0.193995D-03	0.176157D-03	0.00001784	0.09195
110	0	10	4	10	0.140838D-03	0.122533D-03	0.00001830	0.12997
111	0	0	5	0	0.139993D-04	0.150387D-04	-0.00000104	-0.07425
112	0	0	5	1	0.126479D-04	0.137841D-04	-0.00000114	-0.08983
113	0	1	5	2	0.112117D-04	0.125255D-04	-0.00000131	-0.11719
114	0	2	5	3	0.968332D-05	0.112544D-04	-0.00000157	-0.16225
115	0	3	5	4	0.805542D-05	0.996102D-05	-0.00000191	-0.23656
116	0	4	5	5	0.632017D-05	0.863457D-05	-0.00000231	-0.36619
117	0	5	5	6	0.446928D-05	0.726308D-05	-0.00000279	-0.62511
118	0	6	5	7	0.249367D-05	0.583324D-05	-0.00000334	-1.33922
119	0	7	5	8	0.383242D-06	0.433034D-05	-0.00000395	-10.2992
120	0	8	5	9	0.187342D-05	0.273812D-05	-0.00000086	-0.46157
121	0	9	5	10	0.428931D-05	0.103866D-05	0.00000325	0.75785
122	0	1	5	0	0.152732D-04	0.162972D-04	-0.00000102	-0.06705
123	0	2	5	1	0.164767D-04	0.175663D-04	-0.00000109	-0.06613
124	0	3	5	2	0.176171D-04	0.188520D-04	-0.00000123	-0.07010
125	0	4	5	3	0.187014D-04	0.201594D-04	-0.00000146	-0.07796
126	0	5	5	4	0.197366D-04	0.214926D-04	-0.00000176	-0.08897
127	0	6	5	5	0.207294D-04	0.228553D-04	-0.00000213	-0.10255
128	0	7	5	6	0.216859D-04	0.242499D-04	-0.00000256	-0.11823
129	0	8	5	7	0.226113D-04	0.256781D-04	-0.00000307	-0.13563
130	0	9	5	8	0.235102D-04	0.271408D-04	-0.00000363	-0.15442
131	0	8	5	9	0.187342D-05	0.273812D-05	-0.00000086	-0.46157
132	0	10	5	10	0.847034D-05	0.136134D-04	-0.00000514	-0.60719
133	0	0	6	0	0.194158D-04	0.115920D-04	0.00000782	0.40296
134	0	0	6	1	0.192259D-04	0.114659D-04	0.00000776	0.40362
135	0	1	6	2	0.190119D-04	0.113765D-04	0.00000764	0.40161
136	0	2	6	3	0.187725D-04	0.113216D-04	0.00000745	0.39691
137	0	3	6	4	0.185073D-04	0.112985D-04	0.00000721	0.38951
138	0	4	6	5	0.182165D-04	0.113047D-04	0.00000691	0.37943
139	0	5	6	6	0.179007D-04	0.113369D-04	0.00000656	0.36668
140	0	6	6	7	0.175607D-04	0.113919D-04	0.00000617	0.35129
141	0	7	6	8	0.171972D-04	0.114656D-04	0.00000573	0.33329
142	0	8	6	9	0.168102D-04	0.115536D-04	0.00000526	0.31270

143	0	9	6	10	0.163987D-04	0.116508D-04	0.00000475	0.28953
144	0	1	6	0	0.195839D-04	0.117571D-04	0.00000783	0.39965
145	0	2	6	1	0.197332D-04	0.119633D-04	0.00000777	0.39375
146	0	3	6	2	0.198681D-04	0.122126D-04	0.00000766	0.38531
147	0	4	6	3	0.199934D-04	0.125069D-04	0.00000749	0.37445
148	0	5	6	4	0.201150D-04	0.128481D-04	0.00000727	0.36127
149	0	6	6	5	0.202394D-04	0.132379D-04	0.00000700	0.34593
150	0	7	6	6	0.203731D-04	0.136782D-04	0.00000669	0.32862
151	0	8	6	7	0.205231D-04	0.141703D-04	0.00000635	0.30954
152	0	9	6	8	0.206957D-04	0.147158D-04	0.00000598	0.28895
153	0	8	6	9	0.168102D-04	0.115536D-04	0.00000526	0.31270
154	0	10	6	10	0.181887D-04	0.132500D-04	0.00000494	0.27153
155	0	0	7	0	0.117503D-04	0.289267D-05	0.00000886	0.75382
156	0	0	7	1	0.117818D-04	0.294907D-05	0.00000883	0.74969
157	0	1	7	2	0.117831D-04	0.302992D-05	0.00000875	0.74286
158	0	2	7	3	0.117559D-04	0.313513D-05	0.00000862	0.73331
159	0	3	7	4	0.117030D-04	0.326471D-05	0.00000844	0.72104
160	0	4	7	5	0.116291D-04	0.341884D-05	0.00000821	0.70601
161	0	5	7	6	0.115397D-04	0.359778D-05	0.00000794	0.68823
162	0	6	7	7	0.114420D-04	0.380186D-05	0.00000764	0.66773
163	0	7	7	8	0.113436D-04	0.403142D-05	0.00000731	0.64461
164	0	8	7	9	0.112531D-04	0.428678D-05	0.00000697	0.61906
165	0	9	7	10	0.111792D-04	0.456817D-05	0.00000661	0.59137
166	0	1	7	0	0.116888D-04	0.286103D-05	0.00000883	0.75523
167	0	2	7	1	0.115990D-04	0.285461D-05	0.00000874	0.75389
168	0	3	7	2	0.114843D-04	0.287406D-05	0.00000861	0.74974
169	0	4	7	3	0.113493D-04	0.292026D-05	0.00000843	0.74269
170	0	5	7	4	0.112005D-04	0.299427D-05	0.00000821	0.73267
171	0	6	7	5	0.110454D-04	0.309734D-05	0.00000795	0.71958
172	0	7	7	6	0.108931D-04	0.323095D-05	0.00000766	0.70340
173	0	8	7	7	0.107534D-04	0.339672D-05	0.00000736	0.68413
174	0	9	7	8	0.106370D-04	0.359647D-05	0.00000704	0.66189
175	0	8	7	9	0.112531D-04	0.428678D-05	0.00000697	0.61906
176	0	10	7	10	0.106386D-04	0.412366D-05	0.00000651	0.61239
177	0	0	8	0	0.768930D-05	0.491806D-06	0.00000720	0.93604
178	0	0	8	1	0.772606D-05	0.440807D-06	0.00000729	0.94295
179	0	1	8	2	0.770739D-05	0.381357D-06	0.00000733	0.95052
180	0	2	8	3	0.763523D-05	0.313070D-06	0.00000732	0.95900
181	0	3	8	4	0.751298D-05	0.235370D-06	0.00000728	0.96867
182	0	4	8	5	0.734551D-05	0.147497D-06	0.00000720	0.97992
183	0	5	8	6	0.713911D-05	0.485069D-07	0.00000709	0.99321
184	0	6	8	7	0.690151D-05	0.627201D-07	0.00000684	0.99091
185	0	7	8	8	0.664178D-05	0.187474D-06	0.00000645	0.97177
186	0	8	8	9	0.637035D-05	0.327204D-06	0.00000604	0.94864
187	0	9	8	10	0.609894D-05	0.483502D-06	0.00000562	0.92072
188	0	1	8	0	0.759664D-05	0.534551D-06	0.00000706	0.92963
189	0	2	8	1	0.744909D-05	0.569047D-06	0.00000688	0.92361
190	0	3	8	2	0.724908D-05	0.595105D-06	0.00000665	0.91791
191	0	4	8	3	0.700048D-05	0.612340D-06	0.00000639	0.91253
192	0	5	8	4	0.670850D-05	0.620165D-06	0.00000609	0.90756
193	0	6	8	5	0.637970D-05	0.617795D-06	0.00000576	0.90316
194	0	7	8	6	0.602194D-05	0.604233D-06	0.00000542	0.89966

195	0	8	8	7	0.564436D-05	0.578276D-06	0.00000507	0.89755
196	0	9	8	8	0.525735D-05	0.538506D-06	0.00000472	0.89757
197	0	8	8	9	0.637035D-05	0.327204D-06	0.00000604	0.94864
198	0	10	8	10	0.520932D-05	0.694838D-07	0.00000514	0.98666
199	0	0	9	0	0.759132D-05	0.923152D-06	0.00000667	0.87839
200	0	0	9	1	0.760222D-05	0.897097D-06	0.00000671	0.88200
201	0	1	9	2	0.754536D-05	0.871466D-06	0.00000667	0.88450
202	0	2	9	3	0.742185D-05	0.845950D-06	0.00000658	0.88602
203	0	3	9	4	0.723372D-05	0.820104D-06	0.00000641	0.88663
204	0	4	9	5	0.698394D-05	0.793357D-06	0.00000619	0.88640
205	0	5	9	6	0.667643D-05	0.765003D-06	0.00000591	0.88542
206	0	6	9	7	0.631606D-05	0.734180D-06	0.00000558	0.88376
207	0	7	9	8	0.590866D-05	0.699875D-06	0.00000521	0.88155
208	0	8	9	9	0.546110D-05	0.660908D-06	0.00000480	0.87898
209	0	9	9	10	0.498138D-05	0.615929D-06	0.00000437	0.87635
210	0	1	9	0	0.751254D-05	0.949824D-06	0.00000656	0.87357
211	0	2	9	1	0.736662D-05	0.977192D-06	0.00000639	0.86735
212	0	3	9	2	0.715520D-05	0.100521D-05	0.00000615	0.85951
213	0	4	9	3	0.688078D-05	0.103372D-05	0.00000585	0.84977
214	0	5	9	4	0.654661D-05	0.106244D-05	0.00000548	0.83771
215	0	6	9	5	0.615667D-05	0.109098D-05	0.00000507	0.82280
216	0	7	9	6	0.571565D-05	0.111880D-05	0.00000460	0.80426
217	0	8	9	7	0.522892D-05	0.114523D-05	0.00000408	0.78098
218	0	9	9	8	0.470254D-05	0.116945D-05	0.00000353	0.75131
219	0	8	9	9	0.546110D-05	0.660908D-06	0.00000480	0.87898
220	0	10	9	10	0.422301D-05	0.973268D-06	0.00000325	0.76953

Table S9. The reproduction of the quadrupole transition moments QTM-ai=<v"J"|Θ|v',J'> in a.u.) evaluated using the ab initio quadrupole moment function of Somogyi et al.J. Chem. Phys. 2021,155,214303) by the ab initio quadrupole moment function of Piecuch et al. J. Chem. Phys. 1996,104,4699--4715.
Δ=(QTM-ai - QTM-expon)/QTM-ai.

M	v"	J"	v'	J'	QTM-ai	QTM-expon	ai-expon	Δ
1	0	0	0	0	0.174728D+01	0.176981D+01	-0.02253350	-0.01290
2	0	0	0	1	0.174756D+01	0.177012D+01	-0.02255211	-0.01290
3	0	1	0	2	0.174841D+01	0.177102D+01	-0.02260795	-0.01293
4	0	2	0	3	0.174982D+01	0.177252D+01	-0.02270111	-0.01297
5	0	3	0	4	0.175180D+01	0.177463D+01	-0.02283172	-0.01303
6	0	4	0	5	0.175435D+01	0.177735D+01	-0.02299997	-0.01311
7	0	5	0	6	0.175746D+01	0.178067D+01	-0.02320613	-0.01320
8	0	6	0	7	0.176115D+01	0.178460D+01	-0.02345049	-0.01332
9	0	7	0	8	0.176542D+01	0.178915D+01	-0.02373346	-0.01344
10	0	8	0	9	0.177026D+01	0.179432D+01	-0.02405548	-0.01359
11	0	9	0	10	0.177569D+01	0.180010D+01	-0.02441709	-0.01375
12	0	1	0	0	0.174756D+01	0.177012D+01	-0.02255211	-0.01290
13	0	2	0	1	0.174841D+01	0.177102D+01	-0.02260795	-0.01293
14	0	3	0	2	0.174982D+01	0.177252D+01	-0.02270111	-0.01297

15	0	4	0	3	0.175180D+01	0.177463D+01	-0.02283172	-0.01303
16	0	5	0	4	0.175435D+01	0.177735D+01	-0.02299997	-0.01311
17	0	6	0	5	0.175746D+01	0.178067D+01	-0.02320613	-0.01320
18	0	7	0	6	0.176115D+01	0.178460D+01	-0.02345049	-0.01332
19	0	8	0	7	0.176542D+01	0.178915D+01	-0.02373346	-0.01344
20	0	9	0	8	0.177026D+01	0.179432D+01	-0.02405548	-0.01359
21	0	8	0	9	0.177026D+01	0.179432D+01	-0.02405548	-0.01359
22	0	10	0	10	0.177875D+01	0.180337D+01	-0.02461107	-0.01384
23	0	0	1	0	0.195280D+00	0.208185D+00	-0.01290467	-0.06608
24	0	0	1	1	0.192733D+00	0.205605D+00	-0.01287152	-0.06678
25	0	1	1	2	0.190244D+00	0.203090D+00	-0.01284535	-0.06752
26	0	2	1	3	0.187812D+00	0.200638D+00	-0.01282609	-0.06829
27	0	3	1	4	0.185433D+00	0.198246D+00	-0.01281370	-0.06910
28	0	4	1	5	0.183105D+00	0.195913D+00	-0.01280823	-0.06995
29	0	5	1	6	0.180826D+00	0.193636D+00	-0.01280975	-0.07084
30	0	6	1	7	0.178593D+00	0.191412D+00	-0.01281843	-0.07177
31	0	7	1	8	0.176405D+00	0.189239D+00	-0.01283446	-0.07276
32	0	8	1	9	0.174257D+00	0.187116D+00	-0.01285812	-0.07379
33	0	9	1	10	0.172148D+00	0.185038D+00	-0.01288977	-0.07488
34	0	1	1	0	0.197887D+00	0.210832D+00	-0.01294495	-0.06542
35	0	2	1	1	0.200558D+00	0.213550D+00	-0.01299254	-0.06478
36	0	3	1	2	0.203292D+00	0.216340D+00	-0.01304769	-0.06418
37	0	4	1	3	0.206094D+00	0.219205D+00	-0.01311072	-0.06362
38	0	5	1	4	0.208966D+00	0.222148D+00	-0.01318198	-0.06308
39	0	6	1	5	0.211908D+00	0.225170D+00	-0.01326191	-0.06258
40	0	7	1	6	0.214924D+00	0.228275D+00	-0.01335100	-0.06212
41	0	8	1	7	0.218015D+00	0.231465D+00	-0.01344981	-0.06169
42	0	9	1	8	0.221185D+00	0.234744D+00	-0.01355897	-0.06130
43	0	8	1	9	0.174257D+00	0.187116D+00	-0.01285812	-0.07379
44	0	10	1	10	0.198574D+00	0.211900D+00	-0.01332615	-0.06711
45	0	0	2	0	0.169360D-01	0.171784D-01	-0.00024237	-0.01431
46	0	0	2	1	0.169991D-01	0.172616D-01	-0.00026253	-0.01544
47	0	1	2	2	0.170711D-01	0.173504D-01	-0.00027932	-0.01636
48	0	2	2	3	0.171521D-01	0.174449D-01	-0.00029274	-0.01707
49	0	3	2	4	0.172422D-01	0.175450D-01	-0.00030278	-0.01756
50	0	4	2	5	0.173414D-01	0.176508D-01	-0.00030942	-0.01784
51	0	5	2	6	0.174498D-01	0.177624D-01	-0.00031261	-0.01792
52	0	6	2	7	0.175675D-01	0.178798D-01	-0.00031232	-0.01778
53	0	7	2	8	0.176947D-01	0.180031D-01	-0.00030849	-0.01743
54	0	8	2	9	0.178314D-01	0.181325D-01	-0.00030106	-0.01688
55	0	9	2	10	0.179780D-01	0.182679D-01	-0.00028996	-0.01613
56	0	1	2	0	0.168819D-01	0.171007D-01	-0.00021882	-0.01296
57	0	2	2	1	0.168368D-01	0.170286D-01	-0.00019184	-0.01139
58	0	3	2	2	0.168006D-01	0.169620D-01	-0.00016139	-0.00961
59	0	4	2	3	0.167736D-01	0.169010D-01	-0.00012739	-0.00759
60	0	5	2	4	0.167558D-01	0.168455D-01	-0.00008977	-0.00536
61	0	6	2	5	0.167472D-01	0.167957D-01	-0.00004841	-0.00289
62	0	7	2	6	0.167482D-01	0.167514D-01	-0.00000320	-0.00019
63	0	8	2	7	0.167588D-01	0.167128D-01	0.00004599	0.00274
64	0	9	2	8	0.167793D-01	0.166800D-01	0.00009931	0.00592
65	0	8	2	9	0.178314D-01	0.181325D-01	-0.00030106	-0.01688
66	0	10	2	10	0.177372D-01	0.177907D-01	-0.00005346	-0.00301

67	0	0	3	0	0.143897D-02	0.210844D-02	-0.00066947	-0.46524
68	0	0	3	1	0.147108D-02	0.214370D-02	-0.00067262	-0.45723
69	0	1	3	2	0.150443D-02	0.218022D-02	-0.00067579	-0.44920
70	0	2	3	3	0.153909D-02	0.221802D-02	-0.00067893	-0.44112
71	0	3	3	4	0.157513D-02	0.225712D-02	-0.00068199	-0.43297
72	0	4	3	5	0.161264D-02	0.229753D-02	-0.00068489	-0.42470
73	0	5	3	6	0.165169D-02	0.233925D-02	-0.00068756	-0.41627
74	0	6	3	7	0.169238D-02	0.238227D-02	-0.00068989	-0.40764
75	0	7	3	8	0.173479D-02	0.242657D-02	-0.00069177	-0.39877
76	0	8	3	9	0.177902D-02	0.247211D-02	-0.00069309	-0.38959
77	0	9	3	10	0.182518D-02	0.251885D-02	-0.00069368	-0.38006
78	0	1	3	0	0.140803D-02	0.207438D-02	-0.00066635	-0.47325
79	0	2	3	1	0.137820D-02	0.204150D-02	-0.00066330	-0.48128
80	0	3	3	2	0.134940D-02	0.200972D-02	-0.00066032	-0.48934
81	0	4	3	3	0.132160D-02	0.197898D-02	-0.00065738	-0.49742
82	0	5	3	4	0.129473D-02	0.194921D-02	-0.00065448	-0.50550
83	0	6	3	5	0.126873D-02	0.192030D-02	-0.00065158	-0.51357
84	0	7	3	6	0.124356D-02	0.189217D-02	-0.00064861	-0.52158
85	0	8	3	7	0.121916D-02	0.186469D-02	-0.00064553	-0.52949
86	0	9	3	8	0.119550D-02	0.183774D-02	-0.00064224	-0.53721
87	0	8	3	9	0.177902D-02	0.247211D-02	-0.00069309	-0.38959
88	0	10	3	10	0.154521D-02	0.220827D-02	-0.00066306	-0.42911
89	0	0	4	0	0.121665D-03	0.360480D-03	-0.00023882	-1.96290
90	0	0	4	1	0.128492D-03	0.370148D-03	-0.00024166	-1.88071
91	0	1	4	2	0.135597D-03	0.380811D-03	-0.00024521	-1.80841
92	0	2	4	3	0.143002D-03	0.392486D-03	-0.00024948	-1.74462
93	0	3	4	4	0.150733D-03	0.405185D-03	-0.00025445	-1.68810
94	0	4	4	5	0.158815D-03	0.418923D-03	-0.00026011	-1.63780
95	0	5	4	6	0.167278D-03	0.433711D-03	-0.00026643	-1.59276
96	0	6	4	7	0.176149D-03	0.449556D-03	-0.00027341	-1.55214
97	0	7	4	8	0.185459D-03	0.466462D-03	-0.00028100	-1.51517
98	0	8	4	9	0.195242D-03	0.484423D-03	-0.00028918	-1.48114
99	0	9	4	10	0.205531D-03	0.503427D-03	-0.00029790	-1.44940
100	0	1	4	0	0.115093D-03	0.351793D-03	-0.00023670	-2.05661
101	0	2	4	1	0.108755D-03	0.344074D-03	-0.00023532	-2.16376
102	0	3	4	2	0.102632D-03	0.337309D-03	-0.00023468	-2.28658
103	0	4	4	3	0.967061D-04	0.331484D-03	-0.00023478	-2.42775
104	0	5	4	4	0.909588D-04	0.326586D-03	-0.00023563	-2.59048
105	0	6	4	5	0.853741D-04	0.322600D-03	-0.00023723	-2.77866
106	0	7	4	6	0.799363D-04	0.319509D-03	-0.00023957	-2.99704
107	0	8	4	7	0.746304D-04	0.317292D-03	-0.00024266	-3.25151
108	0	9	4	8	0.694419D-04	0.315922D-03	-0.00024648	-3.54945
109	0	8	4	9	0.195242D-03	0.484423D-03	-0.00028918	-1.48114
110	0	10	4	10	0.141908D-03	0.419201D-03	-0.00027729	-1.95403
111	1	0	1	0	0.185073D+01	0.188145D+01	-0.03072171	-0.01660
112	1	0	1	1	0.185102D+01	0.188177D+01	-0.03074548	-0.01661
113	1	1	1	2	0.185190D+01	0.188272D+01	-0.03081686	-0.01664
114	1	2	1	3	0.185336D+01	0.188430D+01	-0.03093603	-0.01669
115	1	3	1	4	0.185541D+01	0.188651D+01	-0.03110327	-0.01676
116	1	4	1	5	0.185804D+01	0.188936D+01	-0.03131900	-0.01686
117	1	5	1	6	0.186126D+01	0.189285D+01	-0.03158376	-0.01697
118	1	6	1	7	0.186508D+01	0.189697D+01	-0.03189821	-0.01710

119	1	7	1	8	0.186948D+01	0.190175D+01	-0.03226314	-0.01726
120	1	8	1	9	0.187449D+01	0.190717D+01	-0.03267950	-0.01743
121	1	9	1	10	0.188009D+01	0.191324D+01	-0.03314836	-0.01763
122	1	1	1	0	0.185102D+01	0.188177D+01	-0.03074548	-0.01661
123	1	2	1	1	0.185190D+01	0.188272D+01	-0.03081686	-0.01664
124	1	3	1	2	0.185336D+01	0.188430D+01	-0.03093603	-0.01669
125	1	4	1	3	0.185541D+01	0.188651D+01	-0.03110327	-0.01676
126	1	5	1	4	0.185804D+01	0.188936D+01	-0.03131900	-0.01686
127	1	6	1	5	0.186126D+01	0.189285D+01	-0.03158376	-0.01697
128	1	7	1	6	0.186508D+01	0.189697D+01	-0.03189821	-0.01710
129	1	8	1	7	0.186948D+01	0.190175D+01	-0.03226314	-0.01726
130	1	9	1	8	0.187449D+01	0.190717D+01	-0.03267950	-0.01743
131	1	8	1	9	0.187449D+01	0.190717D+01	-0.03267950	-0.01743
132	1	10	1	10	0.188369D+01	0.191710D+01	-0.03340710	-0.01773
133	1	0	2	0	0.279936D+00	0.300859D+00	-0.02092244	-0.07474
134	1	0	2	1	0.276138D+00	0.297001D+00	-0.02086297	-0.07555
135	1	1	2	2	0.272415D+00	0.293234D+00	-0.02081916	-0.07642
136	1	2	2	3	0.268765D+00	0.289556D+00	-0.02079085	-0.07736
137	1	3	2	4	0.265183D+00	0.285961D+00	-0.02077796	-0.07835
138	1	4	2	5	0.261667D+00	0.282448D+00	-0.02078052	-0.07942
139	1	5	2	6	0.258213D+00	0.279011D+00	-0.02079860	-0.08055
140	1	6	2	7	0.254816D+00	0.275648D+00	-0.02083238	-0.08175
141	1	7	2	8	0.251472D+00	0.272354D+00	-0.02088212	-0.08304
142	1	8	2	9	0.248178D+00	0.269126D+00	-0.02094815	-0.08441
143	1	9	2	10	0.244929D+00	0.265960D+00	-0.02103090	-0.08587
144	1	1	2	0	0.283814D+00	0.304812D+00	-0.02099782	-0.07398
145	1	2	2	1	0.287774D+00	0.308864D+00	-0.02108941	-0.07328
146	1	3	2	2	0.291820D+00	0.313018D+00	-0.02119765	-0.07264
147	1	4	2	3	0.295955D+00	0.317278D+00	-0.02132301	-0.07205
148	1	5	2	4	0.300181D+00	0.321648D+00	-0.02146611	-0.07151
149	1	6	2	5	0.304503D+00	0.326131D+00	-0.02162761	-0.07103
150	1	7	2	6	0.308922D+00	0.330731D+00	-0.02180828	-0.07059
151	1	8	2	7	0.313443D+00	0.335452D+00	-0.02200902	-0.07022
152	1	9	2	8	0.318067D+00	0.340297D+00	-0.02223078	-0.06989
153	1	8	2	9	0.248178D+00	0.269126D+00	-0.02094815	-0.08441
154	1	10	2	10	0.284203D+00	0.306046D+00	-0.02184267	-0.07686
155	1	0	3	0	0.317153D-01	0.313023D-01	0.00041305	0.01302
156	1	0	3	1	0.318099D-01	0.314362D-01	0.00037369	0.01175
157	1	1	3	2	0.319215D-01	0.315819D-01	0.00033953	0.01064
158	1	2	3	3	0.320500D-01	0.317395D-01	0.00031049	0.00969
159	1	3	3	4	0.321956D-01	0.319091D-01	0.00028653	0.00890
160	1	4	3	5	0.323582D-01	0.320907D-01	0.00026755	0.00827
161	1	5	3	6	0.325381D-01	0.322845D-01	0.00025352	0.00779
162	1	6	3	7	0.327353D-01	0.324909D-01	0.00024436	0.00746
163	1	7	3	8	0.329500D-01	0.327100D-01	0.00024000	0.00728
164	1	8	3	9	0.331824D-01	0.329421D-01	0.00024039	0.00724
165	1	9	3	10	0.334329D-01	0.331875D-01	0.00024545	0.00734
166	1	1	3	0	0.316378D-01	0.311801D-01	0.00045769	0.01447
167	1	2	3	1	0.315775D-01	0.310698D-01	0.00050770	0.01608
168	1	3	3	2	0.315345D-01	0.309713D-01	0.00056315	0.01786
169	1	4	3	3	0.315090D-01	0.308849D-01	0.00062416	0.01981
170	1	5	3	4	0.315014D-01	0.308106D-01	0.00069082	0.02193

171	1	6	3	5	0.315119D-01	0.307487D-01	0.00076326	0.02422
172	1	7	3	6	0.315410D-01	0.306994D-01	0.00084160	0.02668
173	1	8	3	7	0.315891D-01	0.306631D-01	0.00092598	0.02931
174	1	9	3	8	0.316566D-01	0.306401D-01	0.00101655	0.03211
175	1	8	3	9	0.331824D-01	0.329421D-01	0.00024039	0.00724
176	1	10	3	10	0.332142D-01	0.325193D-01	0.00069492	0.02092
177	1	0	4	0	0.323157D-02	0.420328D-02	-0.00097171	-0.30069
178	1	0	4	1	0.329900D-02	0.427474D-02	-0.00097575	-0.29577
179	1	1	4	2	0.336908D-02	0.434784D-02	-0.00097876	-0.29051
180	1	2	4	3	0.344197D-02	0.442266D-02	-0.00098069	-0.28492
181	1	3	4	4	0.351782D-02	0.449930D-02	-0.00098148	-0.27900
182	1	4	4	5	0.359679D-02	0.457785D-02	-0.00098106	-0.27276
183	1	5	4	6	0.367905D-02	0.465842D-02	-0.00097936	-0.26620
184	1	6	4	7	0.376480D-02	0.474110D-02	-0.00097629	-0.25932
185	1	7	4	8	0.385423D-02	0.482600D-02	-0.00097177	-0.25213
186	1	8	4	9	0.394754D-02	0.491323D-02	-0.00096569	-0.24463
187	1	9	4	10	0.404497D-02	0.500293D-02	-0.00095797	-0.23683
188	1	1	4	0	0.316667D-02	0.413334D-02	-0.00096668	-0.30527
189	1	2	4	1	0.310416D-02	0.406484D-02	-0.00096068	-0.30948
190	1	3	4	2	0.304391D-02	0.399766D-02	-0.00095375	-0.31333
191	1	4	4	3	0.298583D-02	0.393170D-02	-0.00094588	-0.31679
192	1	5	4	4	0.292979D-02	0.386686D-02	-0.00093707	-0.31984
193	1	6	4	5	0.287570D-02	0.380301D-02	-0.00092731	-0.32247
194	1	7	4	6	0.282347D-02	0.374006D-02	-0.00091659	-0.32463
195	1	8	4	7	0.277300D-02	0.367788D-02	-0.00090487	-0.32631
196	1	9	4	8	0.272424D-02	0.361636D-02	-0.00089212	-0.32748
197	1	8	4	9	0.394754D-02	0.491323D-02	-0.00096569	-0.24463
198	1	10	4	10	0.346069D-02	0.436846D-02	-0.00090776	-0.26231
199	1	0	5	0	0.355698D-03	0.910327D-03	-0.00055463	-1.55927
200	1	0	5	1	0.372358D-03	0.931173D-03	-0.00055881	-1.50075
201	1	1	5	2	0.389641D-03	0.953026D-03	-0.00056338	-1.44591
202	1	2	5	3	0.407598D-03	0.975901D-03	-0.00056830	-1.39427
203	1	3	5	4	0.426280D-03	0.999805D-03	-0.00057352	-1.34542
204	1	4	5	5	0.445741D-03	0.102474D-02	-0.00057899	-1.29895
205	1	5	5	6	0.466034D-03	0.105068D-02	-0.00058465	-1.25451
206	1	6	5	7	0.487216D-03	0.107762D-02	-0.00059040	-1.21179
207	1	7	5	8	0.509344D-03	0.110552D-02	-0.00059618	-1.17049
208	1	8	5	9	0.532478D-03	0.113436D-02	-0.00060188	-1.13035
209	1	9	5	10	0.556680D-03	0.116409D-02	-0.00060741	-1.09112
210	1	1	5	0	0.339610D-03	0.890463D-03	-0.00055085	-1.62201
211	1	2	5	1	0.324046D-03	0.871547D-03	-0.00054750	-1.68957
212	1	3	5	2	0.308958D-03	0.853536D-03	-0.00054458	-1.76263
213	1	4	5	3	0.294296D-03	0.836378D-03	-0.00054208	-1.84196
214	1	5	5	4	0.280013D-03	0.820009D-03	-0.00054000	-1.92847
215	1	6	5	5	0.266060D-03	0.804360D-03	-0.00053830	-2.02322
216	1	7	5	6	0.252388D-03	0.789347D-03	-0.00053696	-2.12751
217	1	8	5	7	0.238947D-03	0.774880D-03	-0.00053593	-2.24290
218	1	9	5	8	0.225685D-03	0.760859D-03	-0.00053517	-2.37133
219	1	8	5	9	0.532478D-03	0.113436D-02	-0.00060188	-1.13035
220	1	10	5	10	0.400647D-03	0.971723D-03	-0.00057108	-1.42538
221	2	0	2	0	0.195552D+01	0.199713D+01	-0.04161127	-0.02128
222	2	0	2	1	0.195582D+01	0.199746D+01	-0.04164161	-0.02129

223	2	1	2	2	0.195671D+01	0.199845D+01	-0.04173268	-0.02133
224	2	2	2	3	0.195821D+01	0.200009D+01	-0.04188474	-0.02139
225	2	3	2	4	0.196030D+01	0.200240D+01	-0.04209818	-0.02148
226	2	4	2	5	0.196300D+01	0.200538D+01	-0.04237357	-0.02159
227	2	5	2	6	0.196630D+01	0.200901D+01	-0.04271164	-0.02172
228	2	6	2	7	0.197020D+01	0.201332D+01	-0.04311331	-0.02188
229	2	7	2	8	0.197471D+01	0.201829D+01	-0.04357965	-0.02207
230	2	8	2	9	0.197983D+01	0.202394D+01	-0.04411194	-0.02228
231	2	9	2	10	0.198556D+01	0.203027D+01	-0.04471167	-0.02252
232	2	1	2	0	0.195582D+01	0.199746D+01	-0.04164161	-0.02129
233	2	2	2	1	0.195671D+01	0.199845D+01	-0.04173268	-0.02133
234	2	3	2	2	0.195821D+01	0.200009D+01	-0.04188474	-0.02139
235	2	4	2	3	0.196030D+01	0.200240D+01	-0.04209818	-0.02148
236	2	5	2	4	0.196300D+01	0.200538D+01	-0.04237357	-0.02159
237	2	6	2	5	0.196630D+01	0.200901D+01	-0.04271164	-0.02172
238	2	7	2	6	0.197020D+01	0.201332D+01	-0.04311331	-0.02188
239	2	8	2	7	0.197471D+01	0.201829D+01	-0.04357965	-0.02207
240	2	9	2	8	0.197983D+01	0.202394D+01	-0.04411194	-0.02228
241	2	8	2	9	0.197983D+01	0.202394D+01	-0.04411194	-0.02228
242	2	10	2	10	0.198970D+01	0.203475D+01	-0.04505104	-0.02264
243	2	0	3	0	0.345763D+00	0.375366D+00	-0.02960218	-0.08561
244	2	0	3	1	0.340861D+00	0.370370D+00	-0.02950862	-0.08657
245	2	1	3	2	0.336042D+00	0.365482D+00	-0.02943990	-0.08761
246	2	2	3	3	0.331300D+00	0.360696D+00	-0.02939575	-0.08873
247	2	3	3	4	0.326631D+00	0.356007D+00	-0.02937604	-0.08994
248	2	4	3	5	0.322030D+00	0.351411D+00	-0.02938073	-0.09124
249	2	5	3	6	0.317493D+00	0.346903D+00	-0.02940990	-0.09263
250	2	6	3	7	0.313014D+00	0.342477D+00	-0.02946374	-0.09413
251	2	7	3	8	0.308587D+00	0.338129D+00	-0.02954257	-0.09574
252	2	8	3	9	0.304206D+00	0.333853D+00	-0.02964681	-0.09746
253	2	9	3	10	0.299866D+00	0.329643D+00	-0.02977701	-0.09930
254	2	1	3	0	0.350752D+00	0.380473D+00	-0.02972093	-0.08473
255	2	2	3	1	0.355831D+00	0.385697D+00	-0.02986537	-0.08393
256	2	3	3	2	0.361005D+00	0.391042D+00	-0.03003610	-0.08320
257	2	4	3	3	0.366278D+00	0.396512D+00	-0.03023383	-0.08254
258	2	5	3	4	0.371654D+00	0.402113D+00	-0.03045942	-0.08196
259	2	6	3	5	0.377135D+00	0.407849D+00	-0.03071384	-0.08144
260	2	7	3	6	0.382727D+00	0.413725D+00	-0.03099820	-0.08099
261	2	8	3	7	0.388431D+00	0.419745D+00	-0.03131375	-0.08062
262	2	9	3	8	0.394252D+00	0.425914D+00	-0.03166190	-0.08031
263	2	8	3	9	0.304206D+00	0.333853D+00	-0.02964681	-0.09746
264	2	10	3	10	0.350353D+00	0.381406D+00	-0.03105267	-0.08863
265	2	0	4	0	0.484926D-01	0.471408D-01	0.00135177	0.02788
266	2	0	4	1	0.485999D-01	0.473114D-01	0.00128849	0.02651
267	2	1	4	2	0.487335D-01	0.475015D-01	0.00123203	0.02528
268	2	2	4	3	0.488934D-01	0.477112D-01	0.00118227	0.02418
269	2	3	4	4	0.490795D-01	0.479404D-01	0.00113908	0.02321
270	2	4	4	5	0.492917D-01	0.481894D-01	0.00110234	0.02236
271	2	5	4	6	0.495301D-01	0.484582D-01	0.00107195	0.02164
272	2	6	4	7	0.497948D-01	0.487470D-01	0.00104780	0.02104
273	2	7	4	8	0.500860D-01	0.490562D-01	0.00102979	0.02056
274	2	8	4	9	0.504038D-01	0.493860D-01	0.00101782	0.02019

275	2	9	4	10	0.507485D-01	0.497367D-01	0.00101178	0.01994
276	2	1	4	0	0.484119D-01	0.469899D-01	0.00142204	0.02937
277	2	2	4	1	0.483581D-01	0.468587D-01	0.00149944	0.03101
278	2	3	4	2	0.483316D-01	0.467475D-01	0.00158414	0.03278
279	2	4	4	3	0.483328D-01	0.466564D-01	0.00167635	0.03468
280	2	5	4	4	0.483622D-01	0.465859D-01	0.00177626	0.03673
281	2	6	4	5	0.484205D-01	0.465363D-01	0.00188411	0.03891
282	2	7	4	6	0.485083D-01	0.465082D-01	0.00200014	0.04123
283	2	8	4	7	0.486266D-01	0.465020D-01	0.00212462	0.04369
284	2	9	4	8	0.487763D-01	0.465184D-01	0.00225784	0.04629
285	2	8	4	9	0.504038D-01	0.493860D-01	0.00101782	0.02019
286	2	10	4	10	0.507840D-01	0.490624D-01	0.00172161	0.03390
287	2	0	5	0	0.573065D-02	0.685030D-02	-0.00111966	-0.19538
288	2	0	5	1	0.584225D-02	0.696684D-02	-0.00112459	-0.19249
289	2	1	5	2	0.595871D-02	0.708666D-02	-0.00112795	-0.18929
290	2	2	5	3	0.608027D-02	0.720995D-02	-0.00112967	-0.18579
291	2	3	5	4	0.620718D-02	0.733689D-02	-0.00112971	-0.18200
292	2	4	5	5	0.633973D-02	0.746770D-02	-0.00112798	-0.17792
293	2	5	5	6	0.647821D-02	0.760260D-02	-0.00112440	-0.17357
294	2	6	5	7	0.662294D-02	0.774183D-02	-0.00111889	-0.16894
295	2	7	5	8	0.677427D-02	0.788563D-02	-0.00111135	-0.16405
296	2	8	5	9	0.693258D-02	0.803427D-02	-0.00110169	-0.15892
297	2	9	5	10	0.709825D-02	0.818806D-02	-0.00108981	-0.15353
298	2	1	5	0	0.562368D-02	0.673687D-02	-0.00111319	-0.19795
299	2	2	5	1	0.552114D-02	0.662638D-02	-0.00110524	-0.20018
300	2	3	5	2	0.542286D-02	0.651867D-02	-0.00109581	-0.20207
301	2	4	5	3	0.532866D-02	0.641359D-02	-0.00108493	-0.20360
302	2	5	5	4	0.523841D-02	0.631101D-02	-0.00107261	-0.20476
303	2	6	5	5	0.515196D-02	0.621079D-02	-0.00105883	-0.20552
304	2	7	5	6	0.506920D-02	0.611279D-02	-0.00104359	-0.20587
305	2	8	5	7	0.499005D-02	0.601691D-02	-0.00102687	-0.20578
306	2	9	5	8	0.491441D-02	0.592304D-02	-0.00100863	-0.20524
307	2	8	5	9	0.693258D-02	0.803427D-02	-0.00110169	-0.15892
308	2	10	5	10	0.614227D-02	0.716520D-02	-0.00102292	-0.16654
309	2	0	6	0	0.731988D-03	0.153868D-02	-0.00080669	-1.10205
310	2	0	6	1	0.763375D-03	0.157514D-02	-0.00081176	-1.06338
311	2	1	6	2	0.795853D-03	0.161272D-02	-0.00081687	-1.02641
312	2	2	6	3	0.829521D-03	0.165149D-02	-0.00082197	-0.99090
313	2	3	6	4	0.864487D-03	0.169150D-02	-0.00082701	-0.95665
314	2	4	6	5	0.900864D-03	0.173277D-02	-0.00083191	-0.92346
315	2	5	6	6	0.938774D-03	0.177537D-02	-0.00083660	-0.89116
316	2	6	6	7	0.978348D-03	0.181932D-02	-0.00084097	-0.85958
317	2	7	6	8	0.101973D-02	0.186466D-02	-0.00084493	-0.82859
318	2	8	6	9	0.106306D-02	0.191142D-02	-0.00084836	-0.79803
319	2	9	6	10	0.110852D-02	0.195964D-02	-0.00085113	-0.76780
320	2	1	6	0	0.701597D-03	0.150329D-02	-0.00080169	-1.14266
321	2	2	6	1	0.672114D-03	0.146890D-02	-0.00079679	-1.18550
322	2	3	6	2	0.643454D-03	0.143546D-02	-0.00079200	-1.23086
323	2	4	6	3	0.615539D-03	0.140287D-02	-0.00078733	-1.27909
324	2	5	6	4	0.588293D-03	0.137106D-02	-0.00078277	-1.33058
325	2	6	6	5	0.561647D-03	0.133994D-02	-0.00077830	-1.38574
326	2	7	6	6	0.535536D-03	0.130942D-02	-0.00077388	-1.44505

327	2	8	6	7	0.509900D-03	0.127937D-02	-0.00076947	-1.50907
328	2	9	6	8	0.484681D-03	0.124971D-02	-0.00076503	-1.57841
329	2	8	6	9	0.106306D-02	0.191142D-02	-0.00084836	-0.79803
330	2	10	6	10	0.813877D-03	0.161723D-02	-0.00080335	-0.98707
331	3	0	3	0	0.206061D+01	0.211583D+01	-0.05521884	-0.02680
332	3	0	3	1	0.206091D+01	0.211617D+01	-0.05525717	-0.02681
333	3	1	3	2	0.206182D+01	0.211719D+01	-0.05537228	-0.02686
334	3	2	3	3	0.206333D+01	0.211890D+01	-0.05556452	-0.02693
335	3	3	3	4	0.206545D+01	0.212129D+01	-0.05583443	-0.02703
336	3	4	3	5	0.206818D+01	0.212436D+01	-0.05618284	-0.02717
337	3	5	3	6	0.207152D+01	0.212813D+01	-0.05661076	-0.02733
338	3	6	3	7	0.207546D+01	0.213258D+01	-0.05711949	-0.02752
339	3	7	3	8	0.208002D+01	0.213773D+01	-0.05771055	-0.02775
340	3	8	3	9	0.208519D+01	0.214357D+01	-0.05838576	-0.02800
341	3	9	3	10	0.209097D+01	0.215012D+01	-0.05914720	-0.02829
342	3	1	3	0	0.206091D+01	0.211617D+01	-0.05525717	-0.02681
343	3	2	3	1	0.206182D+01	0.211719D+01	-0.05537228	-0.02686
344	3	3	3	2	0.206333D+01	0.211890D+01	-0.05556452	-0.02693
345	3	4	3	3	0.206545D+01	0.212129D+01	-0.05583443	-0.02703
346	3	5	3	4	0.206818D+01	0.212436D+01	-0.05618284	-0.02717
347	3	6	3	5	0.207152D+01	0.212813D+01	-0.05661076	-0.02733
348	3	7	3	6	0.207546D+01	0.213258D+01	-0.05711949	-0.02752
349	3	8	3	7	0.208002D+01	0.213773D+01	-0.05771055	-0.02775
350	3	9	3	8	0.208519D+01	0.214357D+01	-0.05838576	-0.02800
351	3	8	3	9	0.208519D+01	0.214357D+01	-0.05838576	-0.02800
352	3	10	3	10	0.209566D+01	0.215524D+01	-0.05958859	-0.02843
353	3	0	4	0	0.400215D+00	0.439790D+00	-0.03957475	-0.09888
354	3	0	4	1	0.394257D+00	0.433694D+00	-0.03943723	-0.10003
355	3	1	4	2	0.388379D+00	0.427715D+00	-0.03933586	-0.10128
356	3	2	4	3	0.382574D+00	0.421844D+00	-0.03927028	-0.10265
357	3	3	4	4	0.376838D+00	0.416078D+00	-0.03924026	-0.10413
358	3	4	4	5	0.371163D+00	0.410409D+00	-0.03924572	-0.10574
359	3	5	4	6	0.365544D+00	0.404831D+00	-0.03928673	-0.10747
360	3	6	4	7	0.359975D+00	0.399338D+00	-0.03936352	-0.10935
361	3	7	4	8	0.354447D+00	0.393923D+00	-0.03947646	-0.11137
362	3	8	4	9	0.348953D+00	0.388579D+00	-0.03962609	-0.11356
363	3	9	4	10	0.343486D+00	0.383299D+00	-0.03981310	-0.11591
364	3	1	4	0	0.406258D+00	0.446007D+00	-0.03974898	-0.09784
365	3	2	4	1	0.412390D+00	0.452351D+00	-0.03996058	-0.09690
366	3	3	4	2	0.418617D+00	0.458828D+00	-0.04021040	-0.09606
367	3	4	4	3	0.424943D+00	0.465443D+00	-0.04049947	-0.09531
368	3	5	4	4	0.431373D+00	0.472201D+00	-0.04082895	-0.09465
369	3	6	4	5	0.437909D+00	0.479110D+00	-0.04120021	-0.09408
370	3	7	4	6	0.444557D+00	0.486172D+00	-0.04161479	-0.09361
371	3	8	4	7	0.451321D+00	0.493395D+00	-0.04207443	-0.09323
372	3	9	4	8	0.458203D+00	0.500784D+00	-0.04258107	-0.09293
373	3	8	4	9	0.348953D+00	0.388579D+00	-0.03962609	-0.11356
374	3	10	4	10	0.404589D+00	0.446271D+00	-0.04168210	-0.10302
375	3	0	5	0	0.676135D-01	0.649830D-01	0.00263048	0.03890
376	3	0	5	1	0.677090D-01	0.651716D-01	0.00253732	0.03747
377	3	1	5	2	0.678415D-01	0.653887D-01	0.00245281	0.03616
378	3	2	5	3	0.680106D-01	0.656339D-01	0.00237672	0.03495

379	3	3	5	4	0.682162D-01	0.659073D-01	0.00230884	0.03385
380	3	4	5	5	0.684579D-01	0.662089D-01	0.00224897	0.03285
381	3	5	5	6	0.687357D-01	0.665388D-01	0.00219693	0.03196
382	3	6	5	7	0.690495D-01	0.668970D-01	0.00215254	0.03117
383	3	7	5	8	0.693994D-01	0.672838D-01	0.00211566	0.03049
384	3	8	5	9	0.697856D-01	0.676995D-01	0.00208611	0.02989
385	3	9	5	10	0.702081D-01	0.681443D-01	0.00206377	0.02940
386	3	1	5	0	0.675555D-01	0.648230D-01	0.00273252	0.04045
387	3	2	5	1	0.675356D-01	0.646919D-01	0.00284370	0.04211
388	3	3	5	2	0.675544D-01	0.645901D-01	0.00296432	0.04388
389	3	4	5	3	0.676128D-01	0.645181D-01	0.00309467	0.04577
390	3	5	5	4	0.677117D-01	0.644766D-01	0.00323509	0.04778
391	3	6	5	5	0.678521D-01	0.644662D-01	0.00338595	0.04990
392	3	7	5	6	0.680354D-01	0.644878D-01	0.00354764	0.05214
393	3	8	5	7	0.682628D-01	0.645422D-01	0.00372060	0.05450
394	3	9	5	8	0.685359D-01	0.646306D-01	0.00390531	0.05698
395	3	8	5	9	0.697856D-01	0.676995D-01	0.00208611	0.02989
396	3	10	5	10	0.707944D-01	0.676979D-01	0.00309650	0.04374
397	3	0	6	0	0.912855D-02	0.102711D-01	-0.00114253	-0.12516
398	3	0	6	1	0.929278D-02	0.104411D-01	-0.00114832	-0.12357
399	3	1	6	2	0.946481D-02	0.106169D-01	-0.00115210	-0.12172
400	3	2	6	3	0.964498D-02	0.107988D-01	-0.00115381	-0.11963
401	3	3	6	4	0.983365D-02	0.109871D-01	-0.00115341	-0.11729
402	3	4	6	5	0.100312D-01	0.111821D-01	-0.00115085	-0.11473
403	3	5	6	6	0.102381D-01	0.113841D-01	-0.00114607	-0.11194
404	3	6	6	7	0.104547D-01	0.115937D-01	-0.00113901	-0.10895
405	3	7	6	8	0.106816D-01	0.118112D-01	-0.00112963	-0.10576
406	3	8	6	9	0.109192D-01	0.120371D-01	-0.00111787	-0.10238
407	3	9	6	10	0.111682D-01	0.122719D-01	-0.00110367	-0.09882
408	3	1	6	0	0.897181D-02	0.101066D-01	-0.00113477	-0.12648
409	3	2	6	1	0.882230D-02	0.994738D-02	-0.00112508	-0.12753
410	3	3	6	2	0.867976D-02	0.979326D-02	-0.00111350	-0.12829
411	3	4	6	3	0.854396D-02	0.964402D-02	-0.00110007	-0.12875
412	3	5	6	4	0.841469D-02	0.949951D-02	-0.00108481	-0.12892
413	3	6	6	5	0.829180D-02	0.935956D-02	-0.00106776	-0.12877
414	3	7	6	6	0.817512D-02	0.922406D-02	-0.00104894	-0.12831
415	3	8	6	7	0.806454D-02	0.909291D-02	-0.00102837	-0.12752
416	3	9	6	8	0.795997D-02	0.896604D-02	-0.00100607	-0.12639
417	3	8	6	9	0.109192D-01	0.120371D-01	-0.00111787	-0.10238
418	3	10	6	10	0.978029D-02	0.108006D-01	-0.00102031	-0.10432
419	3	0	7	0	0.134715D-02	0.233951D-02	-0.00099236	-0.73664
420	3	0	7	1	0.139937D-02	0.239719D-02	-0.00099783	-0.71306
421	3	1	7	2	0.145364D-02	0.245642D-02	-0.00100278	-0.68984
422	3	2	7	3	0.151013D-02	0.251730D-02	-0.00100717	-0.66694
423	3	3	7	4	0.156901D-02	0.257994D-02	-0.00101093	-0.64431
424	3	4	7	5	0.163045D-02	0.264446D-02	-0.00101401	-0.62192
425	3	5	7	6	0.169464D-02	0.271098D-02	-0.00101634	-0.59974
426	3	6	7	7	0.176178D-02	0.277963D-02	-0.00101785	-0.57774
427	3	7	7	8	0.183208D-02	0.285054D-02	-0.00101846	-0.55591
428	3	8	7	9	0.190575D-02	0.292386D-02	-0.00101812	-0.53424
429	3	9	7	10	0.198301D-02	0.299976D-02	-0.00101674	-0.51273
430	3	1	7	0	0.129683D-02	0.228325D-02	-0.00098641	-0.76063

431	3	2	7	1	0.124828D-02	0.222830D-02	-0.00098002	-0.78510
432	3	3	7	2	0.120135D-02	0.217457D-02	-0.00097322	-0.81011
433	3	4	7	3	0.115591D-02	0.212192D-02	-0.00096602	-0.83572
434	3	5	7	4	0.111183D-02	0.207026D-02	-0.00095843	-0.86202
435	3	6	7	5	0.106900D-02	0.201946D-02	-0.00095046	-0.88911
436	3	7	7	6	0.102729D-02	0.196940D-02	-0.00094211	-0.91708
437	3	8	7	7	0.986584D-03	0.191996D-02	-0.00093338	-0.94607
438	3	9	7	8	0.946762D-03	0.187103D-02	-0.00092427	-0.97624
439	3	8	7	9	0.190575D-02	0.292386D-02	-0.00101812	-0.53424
440	3	10	7	10	0.149549D-02	0.245553D-02	-0.00096004	-0.64196
441	4	0	4	0	0.216480D+01	0.223688D+01	-0.07207020	-0.03329
442	4	0	4	1	0.216511D+01	0.223723D+01	-0.07211858	-0.03331
443	4	1	4	2	0.216601D+01	0.223828D+01	-0.07226391	-0.03336
444	4	2	4	3	0.216752D+01	0.224003D+01	-0.07250661	-0.03345
445	4	3	4	4	0.216964D+01	0.224249D+01	-0.07284750	-0.03358
446	4	4	4	5	0.217236D+01	0.224565D+01	-0.07328764	-0.03374
447	4	5	4	6	0.217569D+01	0.224952D+01	-0.07382847	-0.03393
448	4	6	4	7	0.217962D+01	0.225409D+01	-0.07447176	-0.03417
449	4	7	4	8	0.218416D+01	0.225938D+01	-0.07521959	-0.03444
450	4	8	4	9	0.218930D+01	0.226538D+01	-0.07607448	-0.03475
451	4	9	4	10	0.219505D+01	0.227209D+01	-0.07703930	-0.03510
452	4	1	4	0	0.216511D+01	0.223723D+01	-0.07211858	-0.03331
453	4	2	4	1	0.216601D+01	0.223828D+01	-0.07226391	-0.03336
454	4	3	4	2	0.216752D+01	0.224003D+01	-0.07250661	-0.03345
455	4	4	4	3	0.216964D+01	0.224249D+01	-0.07284750	-0.03358
456	4	5	4	4	0.217236D+01	0.224565D+01	-0.07328764	-0.03374
457	4	6	4	5	0.217569D+01	0.224952D+01	-0.07382847	-0.03393
458	4	7	4	6	0.217962D+01	0.225409D+01	-0.07447176	-0.03417
459	4	8	4	7	0.218416D+01	0.225938D+01	-0.07521959	-0.03444
460	4	9	4	8	0.218930D+01	0.226538D+01	-0.07607448	-0.03475
461	4	8	4	9	0.218930D+01	0.226538D+01	-0.07607448	-0.03475
462	4	10	4	10	0.220029D+01	0.227790D+01	-0.07761137	-0.03527
463	4	0	5	0	0.445379D+00	0.496759D+00	-0.05138058	-0.11536
464	4	0	5	1	0.438375D+00	0.489562D+00	-0.05118635	-0.11676
465	4	1	5	2	0.431439D+00	0.482481D+00	-0.05104234	-0.11831
466	4	2	5	3	0.424563D+00	0.475511D+00	-0.05094805	-0.12000
467	4	3	5	4	0.417741D+00	0.468644D+00	-0.05090316	-0.12185
468	4	4	5	5	0.410964D+00	0.461872D+00	-0.05090757	-0.12387
469	4	5	5	6	0.404226D+00	0.455188D+00	-0.05096139	-0.12607
470	4	6	5	7	0.397518D+00	0.448583D+00	-0.05106494	-0.12846
471	4	7	5	8	0.390831D+00	0.442050D+00	-0.05121878	-0.13105
472	4	8	5	9	0.384155D+00	0.435579D+00	-0.05142366	-0.13386
473	4	9	5	10	0.377481D+00	0.429162D+00	-0.05168056	-0.13691
474	4	1	5	0	0.452456D+00	0.504081D+00	-0.05162578	-0.11410
475	4	2	5	1	0.459611D+00	0.511534D+00	-0.05192292	-0.11297
476	4	3	5	2	0.466851D+00	0.519124D+00	-0.05227315	-0.11197
477	4	4	5	3	0.474180D+00	0.526858D+00	-0.05267791	-0.11109
478	4	5	5	4	0.481604D+00	0.534742D+00	-0.05313885	-0.11034
479	4	6	5	5	0.489125D+00	0.542783D+00	-0.05365787	-0.10970
480	4	7	5	6	0.496749D+00	0.550987D+00	-0.05423715	-0.10918
481	4	8	5	7	0.504479D+00	0.559359D+00	-0.05487914	-0.10878
482	4	9	5	8	0.512319D+00	0.567905D+00	-0.05558659	-0.10850

483	4	8	5	9	0.384155D+00	0.435579D+00	-0.05142366	-0.13386
484	4	10	5	10	0.448967D+00	0.503278D+00	-0.05431102	-0.12097
485	4	0	6	0	0.893489D-01	0.850572D-01	0.00429167	0.04803
486	4	0	6	1	0.894009D-01	0.852396D-01	0.00416128	0.04655
487	4	1	6	2	0.895025D-01	0.854604D-01	0.00404209	0.04516
488	4	2	6	3	0.896528D-01	0.857190D-01	0.00393375	0.04388
489	4	3	6	4	0.898513D-01	0.860153D-01	0.00383594	0.04269
490	4	4	6	5	0.900974D-01	0.863491D-01	0.00374835	0.04160
491	4	5	6	6	0.903908D-01	0.867201D-01	0.00367071	0.04061
492	4	6	6	7	0.907312D-01	0.871285D-01	0.00360274	0.03971
493	4	7	6	8	0.911183D-01	0.875742D-01	0.00354417	0.03890
494	4	8	6	9	0.915521D-01	0.880573D-01	0.00349473	0.03817
495	4	9	6	10	0.920323D-01	0.885781D-01	0.00345414	0.03753
496	4	1	6	0	0.893472D-01	0.849136D-01	0.00443363	0.04962
497	4	2	6	1	0.893969D-01	0.848094D-01	0.00458755	0.05132
498	4	3	6	2	0.894991D-01	0.847453D-01	0.00475387	0.05312
499	4	4	6	3	0.896552D-01	0.847222D-01	0.00493306	0.05502
500	4	5	6	4	0.898666D-01	0.847410D-01	0.00512562	0.05704
501	4	6	6	5	0.901351D-01	0.848030D-01	0.00533212	0.05916
502	4	7	6	6	0.904624D-01	0.849093D-01	0.00555313	0.06139
503	4	8	6	7	0.908507D-01	0.850614D-01	0.00578931	0.06372
504	4	9	6	8	0.913024D-01	0.852610D-01	0.00604134	0.06617
505	4	8	6	9	0.915521D-01	0.880573D-01	0.00349473	0.03817
506	4	10	6	10	0.935500D-01	0.886574D-01	0.00489265	0.05230
507	4	0	7	0	0.135755D-01	0.146661D-01	-0.00109054	-0.08033
508	4	0	7	1	0.138003D-01	0.148974D-01	-0.00109715	-0.07950
509	4	1	7	2	0.140369D-01	0.151381D-01	-0.00110124	-0.07845
510	4	2	7	3	0.142859D-01	0.153886D-01	-0.00110273	-0.07719
511	4	3	7	4	0.145478D-01	0.156493D-01	-0.00110153	-0.07572
512	4	4	7	5	0.148231D-01	0.159206D-01	-0.00109756	-0.07404
513	4	5	7	6	0.151124D-01	0.162032D-01	-0.00109071	-0.07217
514	4	6	7	7	0.154165D-01	0.164974D-01	-0.00108084	-0.07011
515	4	7	7	8	0.157361D-01	0.168040D-01	-0.00106783	-0.06786
516	4	8	7	9	0.160720D-01	0.171235D-01	-0.00105152	-0.06543
517	4	9	7	10	0.164251D-01	0.174568D-01	-0.00103173	-0.06281
518	4	1	7	0	0.133623D-01	0.144438D-01	-0.00108144	-0.08093
519	4	2	7	1	0.131603D-01	0.142302D-01	-0.00106989	-0.08130
520	4	3	7	2	0.129692D-01	0.140251D-01	-0.00105593	-0.08142
521	4	4	7	3	0.127886D-01	0.138282D-01	-0.00103956	-0.08129
522	4	5	7	4	0.126186D-01	0.136393D-01	-0.00102076	-0.08089
523	4	6	7	5	0.124588D-01	0.134583D-01	-0.00099952	-0.08023
524	4	7	7	6	0.123093D-01	0.132851D-01	-0.00097579	-0.07927
525	4	8	7	7	0.121700D-01	0.131195D-01	-0.00094949	-0.07802
526	4	9	7	8	0.120409D-01	0.129615D-01	-0.00092055	-0.07645
527	4	8	7	9	0.160720D-01	0.171235D-01	-0.00105152	-0.06543
528	4	10	7	10	0.145610D-01	0.154891D-01	-0.00092818	-0.06374
529	4	0	8	0	0.225737D-02	0.340654D-02	-0.00114918	-0.50908
530	4	0	8	1	0.233725D-02	0.349231D-02	-0.00115506	-0.49420
531	4	1	8	2	0.242021D-02	0.358068D-02	-0.00116047	-0.47949
532	4	2	8	3	0.250649D-02	0.367185D-02	-0.00116536	-0.46494
533	4	3	8	4	0.259635D-02	0.376601D-02	-0.00116967	-0.45051
534	4	4	8	5	0.269006D-02	0.386340D-02	-0.00117334	-0.43618

535	4	5	8	6	0.278793D-02	0.396425D-02	-0.00117632	-0.42193
536	4	6	8	7	0.289028D-02	0.406882D-02	-0.00117854	-0.40776
537	4	7	8	8	0.299744D-02	0.417738D-02	-0.00117993	-0.39365
538	4	8	8	9	0.310980D-02	0.429023D-02	-0.00118043	-0.37958
539	4	9	8	10	0.322776D-02	0.440772D-02	-0.00117997	-0.36557
540	4	1	8	0	0.218032D-02	0.332319D-02	-0.00114287	-0.52417
541	4	2	8	1	0.210591D-02	0.324209D-02	-0.00113618	-0.53952
542	4	3	8	2	0.203391D-02	0.316305D-02	-0.00112915	-0.55516
543	4	4	8	3	0.196413D-02	0.308594D-02	-0.00112181	-0.57115
544	4	5	8	4	0.189639D-02	0.301060D-02	-0.00111420	-0.58754
545	4	6	8	5	0.183052D-02	0.293688D-02	-0.00110636	-0.60440
546	4	7	8	6	0.176634D-02	0.286465D-02	-0.00109830	-0.62179
547	4	8	8	7	0.170371D-02	0.279377D-02	-0.00109006	-0.63982
548	4	9	8	8	0.164246D-02	0.272413D-02	-0.00108166	-0.65856
549	4	8	8	9	0.310980D-02	0.429023D-02	-0.00118043	-0.37958
550	4	10	8	10	0.248267D-02	0.360264D-02	-0.00111997	-0.45112
551	5	0	5	0	0.226664D+01	0.235952D+01	-0.09287646	-0.04098
552	5	0	5	1	0.226694D+01	0.235987D+01	-0.09293787	-0.04100
553	5	1	5	2	0.226782D+01	0.236095D+01	-0.09312231	-0.04106
554	5	2	5	3	0.226930D+01	0.236274D+01	-0.09343039	-0.04117
555	5	3	5	4	0.227138D+01	0.236524D+01	-0.09386321	-0.04132
556	5	4	5	5	0.227404D+01	0.236846D+01	-0.09442225	-0.04152
557	5	5	5	6	0.227729D+01	0.237240D+01	-0.09510948	-0.04176
558	5	6	5	7	0.228114D+01	0.237706D+01	-0.09592728	-0.04205
559	5	7	5	8	0.228557D+01	0.238245D+01	-0.09687856	-0.04239
560	5	8	5	9	0.229059D+01	0.238855D+01	-0.09796671	-0.04277
561	5	9	5	10	0.229619D+01	0.239539D+01	-0.09919562	-0.04320
562	5	1	5	0	0.226694D+01	0.235987D+01	-0.09293787	-0.04100
563	5	2	5	1	0.226782D+01	0.236095D+01	-0.09312231	-0.04106
564	5	3	5	2	0.226930D+01	0.236274D+01	-0.09343039	-0.04117
565	5	4	5	3	0.227138D+01	0.236524D+01	-0.09386321	-0.04132
566	5	5	5	4	0.227404D+01	0.236846D+01	-0.09442225	-0.04152
567	5	6	5	5	0.227729D+01	0.237240D+01	-0.09510948	-0.04176
568	5	7	5	6	0.228114D+01	0.237706D+01	-0.09592728	-0.04205
569	5	8	5	7	0.228557D+01	0.238245D+01	-0.09687856	-0.04239
570	5	9	5	8	0.229059D+01	0.238855D+01	-0.09796671	-0.04277
571	5	8	5	9	0.229059D+01	0.238855D+01	-0.09796671	-0.04277
572	5	10	5	10	0.230197D+01	0.240191D+01	-0.09993914	-0.04341
573	5	0	6	0	0.481440D+00	0.547104D+00	-0.06566377	-0.13639
574	5	0	6	1	0.473384D+00	0.538781D+00	-0.06539665	-0.13815
575	5	1	6	2	0.465372D+00	0.530570D+00	-0.06519761	-0.14010
576	5	2	6	3	0.457397D+00	0.522462D+00	-0.06506591	-0.14225
577	5	3	6	4	0.449449D+00	0.514450D+00	-0.06500107	-0.14462
578	5	4	6	5	0.441520D+00	0.506523D+00	-0.06500290	-0.14723
579	5	5	6	6	0.433601D+00	0.498672D+00	-0.06507144	-0.15007
580	5	6	6	7	0.425681D+00	0.490888D+00	-0.06520705	-0.15318
581	5	7	6	8	0.417750D+00	0.483161D+00	-0.06541032	-0.15658
582	5	8	6	9	0.409797D+00	0.475479D+00	-0.06568211	-0.16028
583	5	9	6	10	0.401808D+00	0.467832D+00	-0.06602359	-0.16432
584	5	1	6	0	0.489546D+00	0.555546D+00	-0.06599999	-0.13482
585	5	2	6	1	0.497709D+00	0.564116D+00	-0.06640662	-0.13342
586	5	3	6	2	0.505936D+00	0.572821D+00	-0.06688525	-0.13220

587	5	4	6	3	0.514230D+00	0.581668D+00	-0.06743779	-0.13114
588	5	5	6	4	0.522599D+00	0.590665D+00	-0.06806645	-0.13025
589	5	6	6	5	0.531044D+00	0.599818D+00	-0.06877377	-0.12951
590	5	7	6	6	0.539572D+00	0.609134D+00	-0.06956265	-0.12892
591	5	8	6	7	0.548184D+00	0.618620D+00	-0.07043632	-0.12849
592	5	9	6	8	0.556884D+00	0.628282D+00	-0.07139845	-0.12821
593	5	8	6	9	0.409797D+00	0.475479D+00	-0.06568211	-0.16028
594	5	10	6	10	0.483600D+00	0.553231D+00	-0.06963093	-0.14398
595	5	0	7	0	0.113934D+00	0.107551D+00	0.00638276	0.05602
596	5	0	7	1	0.113901D+00	0.107696D+00	0.00620499	0.05448
597	5	1	7	2	0.113931D+00	0.107891D+00	0.00604070	0.05302
598	5	2	7	3	0.114024D+00	0.108134D+00	0.00588936	0.05165
599	5	3	7	4	0.114176D+00	0.108426D+00	0.00575044	0.05036
600	5	4	7	5	0.114389D+00	0.108766D+00	0.00562346	0.04916
601	5	5	7	6	0.114660D+00	0.109152D+00	0.00550790	0.04804
602	5	6	7	7	0.114990D+00	0.109586D+00	0.00540326	0.04699
603	5	7	7	8	0.115376D+00	0.110067D+00	0.00530902	0.04601
604	5	8	7	9	0.115820D+00	0.110595D+00	0.00522467	0.04511
605	5	9	7	10	0.116319D+00	0.111169D+00	0.00514963	0.04427
606	5	1	7	0	0.114031D+00	0.107457D+00	0.00657456	0.05766
607	5	2	7	1	0.114195D+00	0.107414D+00	0.00678097	0.05938
608	5	3	7	2	0.114427D+00	0.107424D+00	0.00700259	0.06120
609	5	4	7	3	0.114728D+00	0.107488D+00	0.00724006	0.06311
610	5	5	7	4	0.115102D+00	0.107608D+00	0.00749406	0.06511
611	5	6	7	5	0.115550D+00	0.107785D+00	0.00776532	0.06720
612	5	7	7	6	0.116076D+00	0.108021D+00	0.00805458	0.06939
613	5	8	7	7	0.116682D+00	0.108319D+00	0.00836267	0.07167
614	5	9	7	8	0.117372D+00	0.108681D+00	0.00869043	0.07404
615	5	8	7	9	0.115820D+00	0.110595D+00	0.00522467	0.04511
616	5	10	7	10	0.119247D+00	0.112153D+00	0.00709352	0.05949
617	5	0	8	0	0.194023D-01	0.202257D-01	-0.00082337	-0.04244
618	5	0	8	1	0.196943D-01	0.205250D-01	-0.00083073	-0.04218
619	5	1	8	2	0.200042D-01	0.208384D-01	-0.00083424	-0.04170
620	5	2	8	3	0.203326D-01	0.211664D-01	-0.00083383	-0.04101
621	5	3	8	4	0.206800D-01	0.215094D-01	-0.00082940	-0.04011
622	5	4	8	5	0.210473D-01	0.218681D-01	-0.00082082	-0.03900
623	5	5	8	6	0.214353D-01	0.222432D-01	-0.00080792	-0.03769
624	5	6	8	7	0.218448D-01	0.226353D-01	-0.00079053	-0.03619
625	5	7	8	8	0.222768D-01	0.230453D-01	-0.00076843	-0.03449
626	5	8	8	9	0.227326D-01	0.234740D-01	-0.00074136	-0.03261
627	5	9	8	10	0.232133D-01	0.239223D-01	-0.00070905	-0.03055
628	5	1	8	0	0.191277D-01	0.199399D-01	-0.00081221	-0.04246
629	5	2	8	1	0.188702D-01	0.196674D-01	-0.00079728	-0.04225
630	5	3	8	2	0.186293D-01	0.194079D-01	-0.00077857	-0.04179
631	5	4	8	3	0.184050D-01	0.191610D-01	-0.00075605	-0.04108
632	5	5	8	4	0.181970D-01	0.189266D-01	-0.00072965	-0.04010
633	5	6	8	5	0.180053D-01	0.187046D-01	-0.00069926	-0.03884
634	5	7	8	6	0.178300D-01	0.184947D-01	-0.00066476	-0.03728
635	5	8	8	7	0.176711D-01	0.182970D-01	-0.00062595	-0.03542
636	5	9	8	8	0.175290D-01	0.181116D-01	-0.00058261	-0.03324
637	5	8	8	9	0.227326D-01	0.234740D-01	-0.00074136	-0.03261
638	5	10	8	10	0.208530D-01	0.214270D-01	-0.00057405	-0.02753

639	5	0	9	0	0.354968D-02	0.488143D-02	-0.00133175	-0.37518
640	5	0	9	1	0.366577D-02	0.500315D-02	-0.00133738	-0.36483
641	5	1	9	2	0.378668D-02	0.512933D-02	-0.00134265	-0.35457
642	5	2	9	3	0.391277D-02	0.526027D-02	-0.00134750	-0.34439
643	5	3	9	4	0.404443D-02	0.539631D-02	-0.00135188	-0.33426
644	5	4	9	5	0.418207D-02	0.553779D-02	-0.00135572	-0.32417
645	5	5	9	6	0.432614D-02	0.568510D-02	-0.00135895	-0.31413
646	5	6	9	7	0.447712D-02	0.583865D-02	-0.00136153	-0.30411
647	5	7	9	8	0.463551D-02	0.599890D-02	-0.00136339	-0.29412
648	5	8	9	9	0.480188D-02	0.616635D-02	-0.00136447	-0.28415
649	5	9	9	10	0.497683D-02	0.634153D-02	-0.00136470	-0.27421
650	5	1	9	0	0.343807D-02	0.476391D-02	-0.00132584	-0.38563
651	5	2	9	1	0.333063D-02	0.465032D-02	-0.00131969	-0.39623
652	5	3	9	2	0.322706D-02	0.454044D-02	-0.00131337	-0.40699
653	5	4	9	3	0.312709D-02	0.443404D-02	-0.00130695	-0.41795
654	5	5	9	4	0.303044D-02	0.433094D-02	-0.00130049	-0.42914
655	5	6	9	5	0.293688D-02	0.423094D-02	-0.00129406	-0.44062
656	5	7	9	6	0.284617D-02	0.413390D-02	-0.00128773	-0.45244
657	5	8	9	7	0.275809D-02	0.403966D-02	-0.00128157	-0.46466
658	5	9	9	8	0.267243D-02	0.394810D-02	-0.00127567	-0.47735
659	5	8	9	9	0.480188D-02	0.616635D-02	-0.00136447	-0.28415
660	5	10	9	10	0.390024D-02	0.521008D-02	-0.00130984	-0.33584
